# α-Glucosidase Inhibition and Molecular Docking Studies of Natural Brominated Metabolites from Marine Macro Brown Alga *Dictyopteris hoytii*

**DOI:** 10.3390/md17120666

**Published:** 2019-11-26

**Authors:** Najeeb Ur Rehman, Kashif Rafiq, Ajmal Khan, Sobia Ahsan Halim, Liaqat Ali, Nadiya Al-Saady, Abdullah Hilal Al-Balushi, Haitham Khamis Al-Busaidi, Ahmed Al-Harrasi

**Affiliations:** 1 Natural & Medical Sciences Research Center, University of Nizwa, P.O Box 33, Birkat Al Mauz, 616 Nizwa, Sultanate of Oman; najeeb@unizwa.edu.om (N.U.R.); kashifrafiq609@gmail.com (K.R.); ajmalkhan@unizwa.edu.om (A.K.); sobia_halim@unizwa.edu.om (S.A.H.); malikhejric@gmail.com (L.A.); 2 Department of Chemistry, Abdul Wali Khan University Mardan, Mardan 23200, Pakistan; 3 Department of Chemistry, University of Mianwali, Mianwali 42200, Pakistan; 4 Oman Animal and Plant Genetic Resources Center, P.O Box 92, 123 Muscat, Oman; nadiya@oapgrc.gov.om (N.A.-S.); abdullah.albalushi@oapgrc.gov.om (A.H.A.-B.);

**Keywords:** α-glucosidase inhibition, *Dictyopteris hoytii*, NMR spectroscopy, molecular docking

## Abstract

Bioassay guided isolation of the methanolic extract of marine macro brown alga *Dictyopteris hoytii* afforded one new metabolite (ethyl methyl 2-bromobenzene 1,4-dioate, **1**), one new natural metabolite (diethyl-2-bromobenzene 1,4-dioate, **2**) along with six known metabolites (**3**–**8**) reported for the first time from this source. The structure elucidation of all these compounds was achieved by extensive spectroscopic techniques including 1D (^1^H and ^13^C) and 2D (NOESY, COSY, HMBC and HSQC) NMR and mass spectrometry and comparison of the spectral data of known compounds with those reported in literature. The in vitro α-glucosidase inhibition studies confirmed compound **7** to be the most active against α-glucosidase enzyme with IC_50_ value of 30.5 ± 0.41 μM. Compounds **2** and **3** demonstrated good inhibition with IC_50_ values of 234.2 ± 4.18 and 289.4 ± 4.91 μM, respectively, while compounds **1**, **5**, and **6** showed moderate to low inhibition. Furthermore, the molecular docking studies of the active compounds were performed to examine their mode of inhibition in the binding site of the α-glucosidase enzyme.

## 1. Introduction

According to World Health Organization (WHO) estimates, about 90% of the world’s diabetic people have type 2, and from 2012 to 2014 about 1.5 million peoples died from complications of this disease [1]. Type 2 diabetes mellitus (T2DM) patients still suffer hyperglycemia and serious complications in spite of having clinically approved anti-diabetic drugs. α-Glucosidase enzyme (EC 3.2.1.20) breaks large non-absorbable macro oligosaccharides into small glucose molecules to make them absorbable in the small intestine. Diabetic disorders can be controlled by reducing the absorption of glucose level via inhibiting α-glucosidase enzyme [2]. Prevention of α-glucosidase enzyme activity suppresses the enhancement of sugar level in the blood after carbohydrate-rich diet intake [3]. However, the therapeutic task of T2DM makes it essential to report new anti-diabetic agents. The clinically used α-glucosidase inhibitors (AGIs), such as acarbose, voglibose and miglitol, successfully decrease the post-prandial glucose levels in T2DM patients [4]. However, these AGIs are associated with a number of problems such as diarrhea, flatulence and abdominal discomfort as well as have high IC_50_ values and low efficacy against α-glucosidase enzyme [4,5]. Due to the significant and vital role of this enzyme in T2DM and side effects of the available drugs, the basic requisite is to discover safe and effective enzyme inhibitors as an approach to effectively control the diabetic disorders.

Recently, bromophenols (BPs) from marine algae were reported to have potential anti-diabetic agents and act as both protein tyrosine phosphatase (PTP1B) and α-glucosidase inhibitors [6]. Some bromophenol derivatives isolated from red algae *Rhodomela confervoides* inhibit PTP1B activity which could decrease the blood glucose level in diabetic rats [7]. Some BPs isolated from the red algae *Symphyocladia latiuscula* are reported to have aldose reductase inhibitory activity and could be used against complications of diabetes, such as eye and nerve damage in T2DM patients [8].

Marine organisms are a rich source of structurally diverse natural products. The search for novel compounds from marine organisms in recent years has produced a number of novel secondary metabolites with pharmaceutical, cosmetics, traditional, and industrial applications [9]. Among these natural products of marine origin, the isolation of halogenated derivatives, especially the bromophenols from seaweeds and macro-algae has been exhaustively reported [10,11]. Recent studies showed that the marine BPs demonstrated a wide range of biological activities [12,13,14,15,16], and therefore the novel BPs have attracted much attention in the field of pharmaceutical agents and functional foods [11].

*Dictyopteris hoytii* W. R. Taylor belongs to family *Dictyotaceae* and is known by its characteristic ocean smell [17]. The genus *Dictyopteris* comprises 35 taxonomically accepted species [18] and has attracted great attention from phytochemists in the middle of the 20th century. This genus has been proved to be an outstanding source of structurally diverse secondary metabolites [19]. Several sesquiterpenes, steroids, sulfated polysaccharides, disulphides, C11-hydrocarbons (sex pheromones) and halogenated compounds are reported from *Dictyopteris* species with potent biological activities [20,21,22,23,24,25,26,27].

In our ongoing efforts to evaluate the phytochemical potential of the Omani marine macroalgae [28,29,30], the present study was conducted to search for effective enzyme inhibitors to effectively control the diabetic disorders. To the best of our knowledge, this is the first report on phytochemical investigation, which resulted in the purification and characterization of two bromobenzene 1,4-dioates (**1** and **2**) along with six known metabolites (**3**–**8**) from the methanol extract of the brown alga *D. hoytii* collected from coastal region of Oman. The structures of compounds (**1** and **2**) were confirmed through combined spectroscopic techniques and mass spectrometry. The known compounds (**3**–**8**) were confirmed through comparison of their spectral values with those reported in literature. These secondary metabolites were further screened against α-glucosidase enzyme, and molecular docking studies were employed to get an insight into the mode of binding of these inhibitors with the enzyme. 

## 2. Results and Discussion

### 2.1. Structure Elucidation of Compounds 1–8

**Figure 1:**
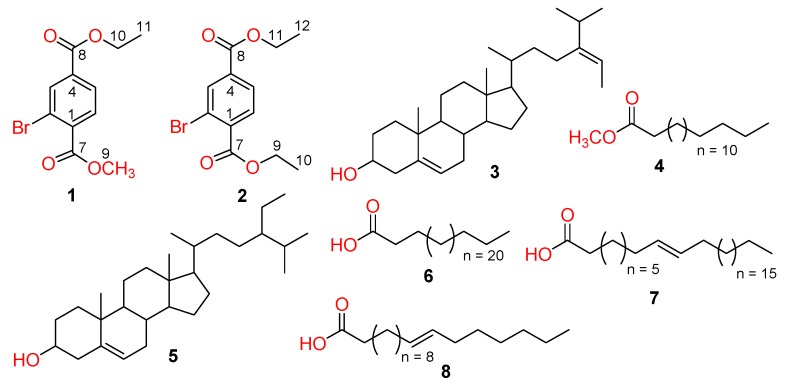
Compounds **1**–**8** isolated from the seaweeds of *D. hoytii.*

Compound **1** was isolated as colorless amorphous powder with the molecular formula C_11_H_11_BrO_4_ as determined on the basis of high-resolution electrospray ionization mass spectrometry (HR-ESI-MS) (Figure 1). The infrared (IR) absorption bands at υ*_max_* 1739, 1725, 1602 and 1433 cm^−1^ indicated the presence of methyl and ethyl esters and aromatic functionalities in the molecule. The ^1^H NMR spectrum of **1** exhibits signals assigned to three aromatic protons at δ 8.28 (1H, d, *J* = 1.2 Hz, H-3), 7.99 (1H, dd, *J* = 8.4, 1.2 Hz, H-5), and 7.79 (1H, d, *J* = 8.4 Hz, H-6) and this is further confirmed from ^13^C NMR spectra (δ 135.1 (C-3), 128.0 (C-5), and 130.9 (C-6)). Moreover, ^1^H-NMR spectrum of **1** also displayed one methyl singlet, one methoxy, one methylene and three aromatic methines. 

The BB ^13^C-NMR spectrum (DEPT-135 and 90) revealed the presence of one methyl, one methylene, three aromatic methines, one methoxy, three aromatic and two carbonyl quaternary carbons. The NMR signals (Table 1) at δC 166.1 (C), 164.5 (C), 61.7 (CH_2_), 52.7 (OCH_3_), 14.2 (CH_3_) and δH 4.39-4.39 (2H, m), 3.94 (3H, s, OCH_3_), 1.40 (3H, t, *J* = 7.2 Hz) showed the presence of ethyl and methyl esters. The HMBC correlations of H-3 with C-2, C-4, and C-5; H-5 with C-4, C-1 and C-6; H-6 with C-5, C-4, C-2 and C-1 further confirmed the presence of ethyl ester at the para position to that of methyl ester. H-5 was ortho to H-6 according to their large coupling constants (J > 7 Hz), while the low coupling constant of 1.2 Hz between H-3 and H-5 indicated that H-3 was meta to H-5. The above links were further confirmed by the ^1^H–^1^H COSY correlations between H-5/H-6 and H-10/11. Therefore, compound **1** was assigned as 2-bromoethylmethylbenzene-1,4-dioate.

Compound **2** was obtained as a colorless amorphous powder with the molecular formula C_12_H_13_BrO_4_, deduced from the HR-ESI-MS and ^13^C-NMR data (Figure 1). The ^1^H-NMR spectrum exhibits signals assigned to three aromatic protons at δ 8.30 (1H, d, *J* = 1.2 Hz, H-3), 8.01 (1H, dd, *J* = 8.4, 1.2 Hz, H-5), and 7.80 (1H, d, *J* = 8.4 Hz, H-6) and this is further confirmed from^13^C-NMR spectra (δ 135.0 (C-3), 128.0 (C-5) and 130.8 (C-6)). Moreover, ^1^H-NMR spectrum also displayed signals for two methyls at δ_H_ 14.2 (C-10) and 14.1 (C-12), and two methylenes at δc 62.0 (C-9) and 61.7 (C-11) and their HMBC correlations with C-7 (δc 165.8) and C-8 (δc 164.5) confirmed the presence of diethyl esters.

**Figure 2:**
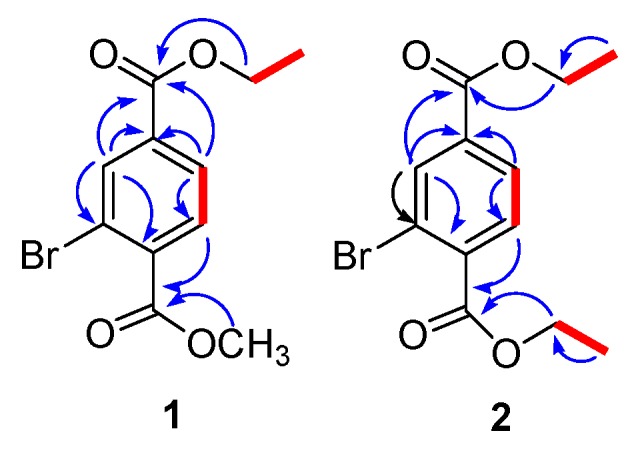
Key HMBC (blue arrow) and H–H COSY (red line) correlations of **1** and **2**.

The ^13^C-NMR spectra indicated the presence of two methyls, two methylenes, three aromatic methines, three aromatic and two carbonyl quaternary carbons. The ^1^H–^1^H and ^1^H–^13^C connectivities were confirmed from the ^1^H–^1^H COSY, HMQC, and HMBC correlations (Figure 2). The HMBC correlations of Me-10 (δ 1.43) with C-7/C-9 and Me-12 (δ 1.40) with C-8/C-11 indicated the presence of diethyl ester groups which were further confirmed through HMBC correlations among H-3 to C-2, C-4, C-1, and C-8; H-5 to C-6, C-4, C-8, and C-1; H-6 to C-5, C-1, C-7, and C-4 (Figure 2). On the basis of above discussion and combined NMR and mass data, the structure of compound **1** was established to be 2-bromodimethylbenzene-1,4-dioate. To the best of our knowledge, both compounds **1** and **2** have not been described earlier as natural products, while compound **2** has been synthesized previously by many researchers [31,32,33]. 

The structures of the known compounds (Figure 1) were determined as fucosterol (**3**) [34,35,36], *n*-hexadecanoic acid, methyl ester (**4**) [37,38], *β*-sitosterol (**5**) [35,39], cerotic acid (**6**) [40,41], *n*-octacos-9-enoic acid (**7**) [42,43], and 11-eicosenoic acid (**8**) [44,45], on the basis of their spectral data and the comparison of their spectral data with those reported in literature. 

### 2.2. α-Glucosidase Inhibition 

The α-glucosidase enzyme inhibition by compounds **1**–**8** were determined up to 1.0 mM concentration to assess their biological potential (Table 1). In the preliminary screening, six compounds (**1**–**3** and **6**–**8**) demonstrated notable in vitro α-glucosidase inhibitory properties with IC_50_ values ranging from 30.5 ± 0.41–659.78 ± 2.15 μM, while compounds having less than 50% inhibition were not tested for IC_50_. Comparing brominated compounds, compound **2** (234.2 ± 4.18 µM) showed higher inhibition than **1** (522.0 ± 0.51µM), which may be due to the replacement of methoxy with ethyl group. Compound **2** was found more active than standard acarbose (IC_50_ = 942 ± 0.74 μM). 

Among isolated fatty acids, compound **7**, carrying COOH group, exhibited higher actions (IC_50_ = 30.5 ± 0.41 μM) **6** (659.78 ± 2.15) carrying double bonds at C-9, indicating that the higher activity of compound **7** may be due to presence of double bond. Similarly, comparing compound **7** with **4** (inactive), loss of activity in compound **4** may be due to the replacement of hydroxyl group with -OCH_3_ at C-1 position and absence of double bond. These results further indicated the presence of carboxyl function in the saturated fatty acids as the key feature for α-glucosidase activity. 

### 2.3. Molecular Docking of α-Glucosidase Inhibitors

**Figure 3:**
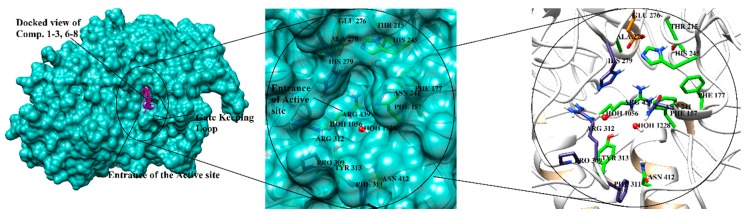
Structural topology of *S. cerevisiae* α-glucosidase is displayed in 3D-form. The entrance of the groove is highlighted. The catalytic triad (Asp214, Glu276, Asp349) are depicted in orange sticks, while active site residues (that surround catalytic triad) are displayed in green stick model. The gate keeping residues are shown in blue stick model. The docking mode of compounds **1**–**3** and **6**–**8** are shown in magenta stick model within the binding cavity in the left panel.

Compounds **1**–**3** and **6**–**8** exhibited in vitro α-glucosidase enzyme inhibitory activities, and hence, their mode of binding was predicted by molecular docking within the active site of *S. cerevisiae* α-glucosidase enzyme. Because of the unavailability of *S. cerevisiae* α-glucosidase structure, docking was carried out on the homology model of *S. cerevisiae* α-glucosidase. In our previous studies [2], three-dimensional coordinates of *S. cerevisiae* α-glucosidase was developed by homology modeling, which is used in this study for structure based binding studies. The model is composed of a total of 579 residues. The Ramachandran plot showed that 444 (86.7%), 63 (12.3%), 3 (0.6%), and two (0.4%) residues lied in the most favored, additional allowed, generously allowed, and disallowed regions, respectively. ERRAT showed 93.52 quality factor, while Verify3D depicted that 95.5% residues showed average 3D–1D score of 0.7. Thus, the model can be used in structural studies because of its good quality. The active site of the enzyme was predicted by aligning model on its closest homologue *S. cerevisiae* isomaltase (72% identity) in complex with isomaltose (PDB code: 3AXH). In the active site of *S. cerevisiae* α-glucosidase, Asp214, Glu276 and Asp349 formed catalytic triad. For substrate molecules, Asp214 act as nucleophile and Glu276 work as proton donor, while Asp349 stabilize the transition state of substrate. The lining of active site is constituted by both hydrophobic and hydrophilic residues including Ala278, Phe300, His348, Gln350, Asp408, Arg439, and Arg443 which are mainly involve in the stabilization of substrate by providing hydrophilic interactions. Moreover, enzyme-substrate is stabilizing by water-mediated bridging. Particularly Wat1021, Wat1026, Wat1056, Wat1058, Wat1061, Wat1087, Wat1102, Wat1122, Wat1174, and Wat1228 are important molecules. The entrance of the active site gorge has gate keeping residues (Phe231, His239, Asn241, His279, Glu304, and Arg312) that regulate the entry and exit of substrate in the active site. The structural topology of the *S. cerevisiae* α-glucosidase is shown in Figure 3. 

**Figure 4:**
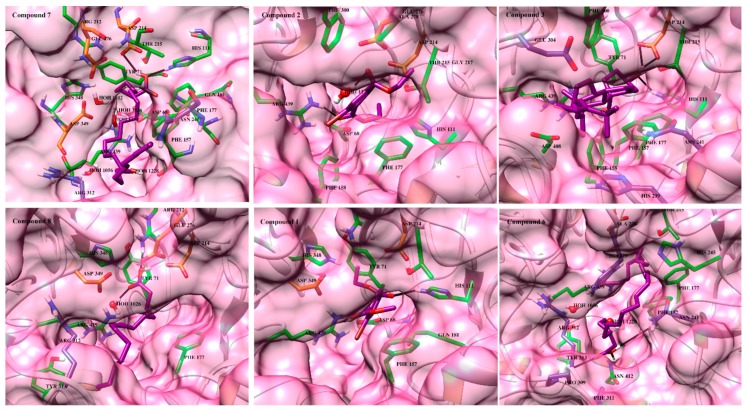
Docked view of the isolated compounds are shown in the active site of α-glucosidase. Catalytic residues, active site residues and gate keeping residues are displayed in orange, green and blue stick model, respectively. Compounds **1**–**3** and **6**–**8** are shown in magenta stick model. Hydrogen bonds are depicted in black lines. Hydrogen bond distances are tabulated in Table 2.

Compound **7** exhibited the highest activity against α-glucosidase followed by compounds **2** and **3**. The binding mode of **7** depict that the acetate moiety of this compound is essential for binding with the catalytic residues. The acetate –OH group acts as hydrogen bond donor to the side chain of Asp214, thus destabilize the catalytic triad for further action. Additionally, –OH accepts H-bond from the side chain of His111 that further strengthen binding of this ligand with in the active site. While the long carbon chain neatly fits at the entrance of the binding cavity via hydrophobic interactions, thus blocked the access of catalytic residues. The docked view of this compound is presented in Figure 4. The docking score and binding interactions are tabulated in Table 2. 

Compounds **2** and **3** showed biological activities in the range of 234 µM and 289 µM, respectively. The ethyl ester moieties of diethyl 2-bromobenzene 1,4-dioate (**2**) interacts with Arg439 anda water molecule (Wat1174) via hydrogen bonding. Similar to the acetate moiety of compound **1**, the –OH of fucosterol (**3**) mediates bidentate interactions with the side chains of Asp214 and accepts H-bond through the side chain of His111**,** while its sterol skeleton is fixed at the lining of active site via hydrophobic interactions provided by Phe157, Phe158, Phe177, Phe300, His239 and His279. Compound **8** exhibited an IC_50_ value of 480µM. The acetate moiety of this compound is titled towards Arg212 instead of Asp214. The compound mediates H-bonding with the side chain of Arg212 and accepts H-bond from surrounding water molecule 1026. Water molecule 1026 bridges the ligand with Asp68 and Arg443. The long carbon chain is aligned with the lining of active site gorge. The ethyl acetate moiety of ethyl methyl 2-bromobenzene 1,4-dioate (**1**) interacts with the side chain of His111, while methyl acetate does not interact with the surrounding residues. The loss of interaction of this compound with Arg439 and water molecule may be the reason of lower activity of this compound as compared to compound **2**. The docked view of the least active compound **6** depicts that this compound does not enter deep into the active site cavity, however resided at the entrance loop and interact with the side chains of Phe157 and Tyr313. Since the acetate moiety does not interact with the catalytic residue, this compound has shown the least inhibition as compare to compounds **7** and **8**. It indicates that the increase in the flexibility of structure leads to decrease activity of this compound due to steric hindrance. Moreover, a known inhibitor, Acarbose, was also docked into the *S. cerevisiae* α-glucosidase model to predict its binding mechanism and to compare its binding with the binding of compounds **1**–**3** and **6**–**8**. Acarbose showed high interactions within the active site due to its high number of –OH moieties; however, the binding score of this compound is very low (−1.24). This suggests that acarbose is highly selective for human α-glucosidase as compare to *S. cerevisiae* α-glucosidase. The docked view of acarbose in the active site of *S. cerevisiae* α-glucosidase model is shown in supporting information Figure S1. The docking score and the binding interactions of these compounds are in complete agreement with the experimental findings (Table 2). 

Additionally, compound **1**–**3** and **6**–**8** were docked at human *α*-glucosidase active site to predict their binding mechanism on human *α*-glucosidase. The active site of human *α*-glucosidase is composed of mainly acidic (Asp404, Asp518, Glu521 and Asp616) and basic residues (Arg600 and His674). Before docking of our inhibitors, acarbose was re-docked into the active site. Acarbose showed similar interactions and orientations as it is present in its crystallographic form. 

The structure of human α-glucosidase is similar to the structures of human glycoside hydrolase family GH311 homologues, maltase glucoamylase and sucrase-isomaltase. A N-terminal trefoil Type-P domain is linked with a domain composed of β-sheet which is followed by the catalytic (β/α)8 barrel bearing two inserts β3 (insert I) and β4 (insert II), and proximal and distal β-sheet domains at the C-terminus. The substrate-binding pocket is narrow and located near the C-terminal ends of β-strands of the catalytic (β/α)8 domain and shaped by a loop from the N-terminal β-sheet domain and both inserts I and II. Asp518 and Asp616 serves as the catalytic nucleophile and catalytic acid/base, respectively. Studies showed that a pharmacological chaperone 1-deoxynojirimycin and its derivative N-hydroxyethyl-deoxynojirimycin binds with Asp404, Asp518, Arg600, Asp616 and His674 by H-bonds. Moreover, Trp376, Ile441, Trp516, Met519, Trp613, and Phe649 provides hydrophobic interactions to these molecules. The inhibitor acarbose also binds with Asp616 by H-bond, while its 2-OH and 3-OH interact with conserved Arg600 and Asp282. The maltose of acarbose interacts with side chain Trp618 via water mediated bridging. Several studies showed that the mutation of Arg600 to Cys or His leads to loss of activity of this enzyme [46,47,48].

The docked view of compounds **1**–**3** and **6**–**8** showed that all these compounds showed good binding interactions. Compounds **1**–**3** interact with Arg600 by H-bonding. Compound **3** also mediates H-bonding with Asp616. Compounds **6** and **8** were found to be H-bonded with Asp518. Additionally, compound **8** also formed H-bond with the side chain of His674. The docked view of compound **7** showed that its carboxylate moiety interacts with the side chain of Asp404 and a water molecule (Wat11060). By docking score, we assume that compound **7** (−9.08) will show high activity on human *α*-glucosidase, followed by compounds **8** (−7.23) and **3** (−7.07), while compounds **2** (−6.44) and **6** (−6.26) would be moderately active and compound **1** (−6.41) will be least active. The docking scores and predicted binding interactions of compounds **1**–**3** and **6**–**8** are tabulated in Table 2. The binding interactions of these compounds in the active site of human *α*-glucosidase are shown in supporting information Figure S2.

Moreover, structure-based pharmacophore modeling was applied using the *S. cerevisiae* in complex with its substrate (isomaltose) and acarbose. Two pharmacophore models were generated, namely Ph-1 (created by isomaltose) and Ph-2 (created by acarbose). Ph-1 was composed of seven hydrogen bond donors/acceptors (Don/Acc), one h-bond acceptor (Acc), and two hydrophobic (Hyd) features. The Don/Acc features are required in the compound to bind Asp214, Glu276, Asp349, His111, Arg212, Arg312, His348, Asp408, and five water molecules. Ph-2 was composed of five Don/Acc, three Hyd, and one Acc features which are necessary in acarbose to bind with Asp214, Arg212, Glu276, His348, Asp408, few water molecules, Phe177, Phe157, Thr215, Phe300, Phe310, and His239. The docked conformation of each compound (**1**–**3** and **6**–**8**) was superimposed on both Ph-1 and Ph-2. Compound **1** was superimposed on two Don/Acc, and one Hyd on Ph-1 and one Don/Acc, and one Acc on Ph-2. Compound **2** was aligned on one Don/Acc and one Hyd on Ph-1 and one Don/Acc on Ph-2. On Ph-1 and Ph-2, compound 3 was aligned on one Don/Acc, one Hyd, and three Hyd, respectively. Compound **6** adapt inverted position and its carboxylate group is oriented towards the opening channel of active site, thus its long chain alkyl moeity was superimposed on two Hyd of Ph-1 and three Hyd of Ph-2. Compound **7** is the most active molecule in this study. Its superimposed view on Ph-1 depicts that this compound was aligned on one Don/Acc, and two Hyd of Ph-1. This Don/Acc is essential for **7** to bind with His111 and Arg212. However, this compound was superimposed on two Hyd of Ph-2. Compound **8** was aligned on two Don/Acc and two Hyd on Ph-1, and one Don/Acc, and two Hyd of Ph-2.

Both pharmacophore models are shown in supporting information Figure S3 and S4. The alignment of each ligand on Ph-1 and Ph-2 are presented in Figures S5–S16. The pharmcaphore alignment is well correlated with the docking interactions. It suggests that our new inhibitors possess some of the important pharmacophoric features which are responsible for binding, in turn producing inhibitory effects.

### 2.4. Pharmakinetic Prediction of α-Glucosidase Inhibitors

The pharmacokinetic behavior of the compounds was predicted in silico by three different parameters including human intestinal absorption, human oral bioavailability and absorption in human epithelial colorectal adenocarcinoma cell lines (Caco-2). The predicted human intestinal absorption (HIA) of compounds **1**, **2**, and **8** are high while compounds **3**, **6** and **7** showed low HIA. Compounds **1**–**3**, and **8** demonstrated good Caco-2 absorption. Compounds **1**–**2** exhibited high human oral bioavailability, while the rest showed no bioavailability. All the compounds depicted non-carcinogenic behavior. Their AMES mutagenesis and hepatotoxicity score also suggests that these compounds are non-mutagenic and non-hepatotoxic in nature. Moreover, none of the compound acts as a substrate or inhibitor of P-glycoprotein. All the compounds except **2**, **3** and **6** are biodegradable. Their calculated acute oral toxicity depicts that these compounds are not toxic. Furthermore, their metabolic profile shows that these compounds does not have cytochrome P450 inhibitory promiscuity. Similarly, all the compounds follow Lipinski rule of five, which suggests that these compounds possess good drug likeness properties. The predicted ADMET properties of compounds **1**–**3** and **6**–**8** are tabulated in Table 3.

## 3. Material and Methods

### 3.1. General

ESI-HRMS (positive) spectra were documented on Agilent Technologies (6530, Accurate Mass Q-TOF LC/MS). ATR-FTIR (solid) spectra were recorded on a Bruker, ATR-Tensor 37 spectrophotometer with wave numbers (ν) in cm^−1^. Optical rotations were recorded on a KRUSS P3000 polarimeter purchased from A. Kruss Optronic, Germany. The ^1^H (600 MHz) and ^13^C (150 MHz) NMR spectra were measured on BRUKER AVANCE NMR spectrometers (600 MHz) using solvent peaks (CDCl_3_, δ_H_: 7.26; δ_C_: 77.0), (CD_3_OD, δ_H_: 4.87; δ_C_: 48.5) as internal reference. Data were reported in the following order: multiplicities are indicated as s = singlet, m = multiplet, t = triplet, dd = doublet of doublet, d = doublet; chemical shift (δ) in ppm; coupling constants (J) are in hertz (Hz). Column chromatography was applied by using 100–200 mesh silica gel. For thin layer chromatography, pre-coated aluminum sheets (silica gel 60F-254, E. Merck) were used. The compounds were visualization with UV-light (254 and 366 nm) or I_2_ stain and also by spraying with the ceric sulfate reagent.

### 3.2. Sample Collection and Identification 

The seaweed samples (*D. hoytii*) were collected in April, 2017 from various locations along the coastal region of Raysut (16°59′10.04″ N, 54°1′58.59″ E), Dhofar, Oman, and were provided by Oman Animal and Plant Genetic Resource Center. The voucher number (DHS-04/2017) was deposited in the herbarium unit at Natural and Medical Sciences Research Center, University of Nizwa, Nizwa, Oman. The fresh samples were rinsed with distilled water (DW) and transported in cool boxes to the laboratory. The cleaned material was freeze dried and ground to make powder and stored at −20 °C until further analysis.

### 3.3. Extraction, Isolation and Purification 

The freeze-dried samples of *D. hoytii* (2.3 kg) were extracted exhaustively in 80% methanol (6 L) at room temperature (3 × 10 days) and evaporated under low pressure to yield a crude MeOH extract (500 g). The obtained MeOH extract was directly loaded over the silica gel (70–230 mesh; Merck) column chromatography (CC) using *n*-hexane, *n*-hexane/ethyl acetate, and ethyl acetate/MeOH with 10% increasing polarity to afford fourteen fractions (DHF_1_–DHF_14_). After taking TLC, three fractions (DHF_1_–DHF_3_; 10–20% *n*-hexane/ethyl acetate) were combined and subjected to further CC using *n*-hexane/ethyl acetate with increasing polarity (1:9, 2:8, 4:6, and 6:4) to afford four sub-fractions (HF_A_–HF_D_). HF_B_ (10% *n*-hexane/ethyl acetate) was further subjected to CC and preparative TLC to afford compound **1** (8.0 mg) and **2** (6.5 mg) along with known compounds **4** (14.5 mg), **6** (22.5 mg), **7** (12 mg), and **8** (10.5 mg). Similarly, fraction HF_D_ was further loaded over silica gel CC to get compounds **3** (16.5 mg) and **5** (28.0 mg) using solvent system of 20% *n*-hexane/ethyl acetate. 

Ethyl methyl 2-bromobenzene 1,4-dioate (**1**): NMR data of compound **1**: colorless amorphous powder (4.5 mg); UV (MeOH)λ*_max_* 264 (4.51), 285 (4.22); IR (solid) υ*_max_* 2969 (CH), 1739, 1725 (esters group), 1602 (C=C), 1433, 1283, 1148, 1040, 854, 745 cm^−1^; ^1^HNMR (CDCl_3,_ 600 MHz): δ 8.28 (1H, d, *J* = 1.2 Hz, H-3), 7.99 (1H, dd, *J* = 8.4, 1.2 Hz, H-5), 7.79 (1H, d, *J* = 8.4 Hz, H-6), 4.39–4.39 (2H, m), 3.94 (3H, s, OCH_3_), 1.40 (3H, t = 7.2 Hz); ^13^C-NMR (CDCl_3_, 125 MHz): δ 166.1 (C-7), 164.5 (C-8), 136.0 (C-4), 135.1 (C-3), 134.0 (C-1), 130.9 (C-6), 128.0 (C-5), 121.4 (C-2), 61.7 (C-10), 52.7 (C-9), 14.2 (C-11); HR-ESI-MS (*m/z)*: 308.9748 [^79^BrM + Na]^+^, 310.9728 ([^81^BrM + Na]^+^, C_11_H_11_BrNaO_4_).

Diethyl 2-bromobenzene 1,4-dioate (**2**): colorless amorphous powder (6.0 mg); UV (MeOH)λ*_max_* 262 (4.20), 254 (4.20); IR (solid) υ*_max_* 2950 (CH), 1738, 1730 (esters group), 1604 (C=C), 1464, 1236, 1122, 1040, 943, 715 cm^−1^; ^1^H-NMR (CDCl_3,_ 600 MHz): δ 8.30 (1H, d, *J* = 1.2 Hz, H-3), 8.01 (1H, dd, *J* = 8.4, 1.2 Hz, H-5), 7.80 (1H, d, *J* = 8.4 Hz, H-6), 4.44–4.39 (4H, m), 1.43–1.40 (6H, m); ^13^C-NMR (CDCl_3_, 125 MHz): δ 165.8 (C-7), 164.5 (C-8), 136.4 (C-4), 135.0 (C-3), 133.9 (C-1), 130.8 (C-6), 128.0 (C-5), 121.2 (C-2), 62.0 (C-9), 61.7 (C-11), 14.2 (C-10), 14.1 (C-12); HR-ESI-MS (*m/z)*: 300.9937 [^79^BrM + H]^+^, 302.9916 ([^81^BrM + H]^+^, C_12_H_14_BrO_4_) [31,32,33].

Fucosterol (**3**): HR-ESI-MS (*m/z)*: 395.3651 [M −H_2_O + H]; ^1^H-NMR (CDCl_3,_ 600 MHz): δ 5.33 (1H, br. d, *J* = 2.4 Hz, H-6), 5.17 (1H, q, *J* = 6.6 Hz, H-28), 3.52 (1H, m, H-3), 1.56 (3H, br s, H-21), 1.03 (3H, s, H-19), 0.97 (3H, br s, H-21), 0.96 (3H, d, *J* = 1.2 Hz, H-27), 0.90 (3H, d, *J* = 1.2 Hz, H-26), 0.66 (3H, s, H-18); ^13^C-NMR (CDCl_3_, 125 MHz): see references for NMR data [34,35,36].

*n*-Hexadecanoic acid, methyl ester (**4**): HR-ESI-MS (*m/z)*: 271.2631 [M + H]; ^1^H-NMR (CDCl_3,_ 600 MHz): δ 3.61 (OCH_3_), 2.26 (2H, t, *J* = 7.2 Hz, H-2), 1.57 (2H, m, H-3), 0.84 (3H, t, *J* = 6.6 Hz, C-16); ^13^C-NMR (CDCl_3_, 125 MHz): δ 174.19, 51.3, 34.0, 31.9, 29.7-29.0, 24.9, 22.6, 14.0 [37,38].

*β*-Sitosterol (**5**): HR-ESI-MS (*m/z)*: 397.3831 [M −H_2_O + H]; 5.32 (1H, br.s, H-6), 3.51 (1H, m, H-3), 1.03 (3H, s, H-19), 0.94 (3H, d, *J* = 6.2 Hz, H-21), 0.84 (3H, d, *J* = 6.6 Hz, H-26), 0.82 (overlapping, t, H-29), 0.86 (3H, d, *J* = 6.6 Hz, H-27) 0.70 (3H, s, H-18); ^13^C-NMR (CDCl_3_, 125 MHz): see references for NMR data [35,39]. 

Cerotic acid (**6**): HR-ESI-MS (*m/z)*: 419.3078 [M + Na]; ^1^H-NMR (CDCl_3,_ 600 MHz): δ 2.33 (2H, t, *J* = 7.2 Hz, H-2), 1.62 (2H, m, H-3), 0.84 (3H, t, *J* = 6.6 Hz, C-16); ^13^C-NMR (CDCl_3_, 125 MHz): δ 180.1, 34.0, 31.9, 29.6–29.0, 24.6, 22.6 [40,41]. 

*n*-Octacos-9-enoic acid (**7**): HR-ESI-MS (*m/z)*: 445.3661 [M + Na]; 447.3814 [M + 2H + Na]; ^1^H-NMR (CDCl_3,_ 600 MHz): δ 5.32 (4H, br.s, H-11/12), 2.33 (2H, t, *J* = 7.2 Hz, H-2), 1.99 (2H, br.s, H-10/13), 1.61 (2H, m, H-3), 0.85 (3H, t, *J* = 6.6 Hz, H-28); ^13^C-NMR (CDCl_3_, 125 MHz): δ 179.2, 130.0, 129.7, 34.0, 31.9, 29.7–29.1, 24.7, 22.7, 14.1 [42,43].

11-Eicosenoic acid (**8**): HR-ESI-MS (*m/z)*: 333.2470 [M + Na]; ^1^H-NMR (CDCl_3,_ 600 MHz): δ 5.32 (4H, br.s, H-9/10), 2.32 (2H, t, *J* = 7.2 Hz, H-2), 1.99 (2H, br.s, H-8/11), 1.62 (2H, m, H-3), 0.85 (3H, t, *J* = 6.6 Hz, H-18); ); ^13^C-NMR (CDCl_3_, 125 MHz): δ 180.4, 129.98, 129.69, 34.1, 31.9, 29.7–29.0, 24.6, 22.6, 14.0 [44,45].

### 3.4. In Vitro α-Glucosidase Inhibition 

*α*-Glucosidase (E.C.3.2.1.20) enzyme (*Saccharomyces cerevisiae*) inhibition activity was carried out by using previously described method [2]. The enzyme (0.2 μ/mL) solution in phosphate buffered saline (PBS) (0.1 M, pH 6.8) with different concentrations of tested compounds (1, 0.5, 0.25, 0.125, 0.0625 and 0.0312 mM) at 37 °C were incubated for 15 min. After incubation, 0.7 mM *p*-nitrophenyl-*α*-d-glucopyranoside (a chromogenic substrate) was added and the change in absorbance was recorded at 400 nm for 30 min by microplate spectrophotometer (xMark™, BIO-RAD, California, LA, United States) per production of *p*-nitrophenyl. Al the isolated compounds were dissolve in DMSO (7.5% final) along with control. Acarbose with IC_50_ of 942 ± 0.74 μM was used as standard inhibitor. The IC_50_ values were calculated by using different concentrations of tested compounds. The results were processed by MS-Excel and Ez-fit software programs. The % inhibition was calculated by using the following formula:

% Inhibition = 100 −(OD test well/OD control) × 100.

### 3.5. Molecular Docking and Pharmacokinetic Prediction

Compounds **1**–**3** and **6**–**8** exhibited inhibitory activity against α-glucosidase enzyme. Their mode of action was predicted by the computational molecular docking approach. Docking was carried out by molecular operating environment (MOE) docking suite [49]. We developed homology model of *Saccharomyces cerevisiae* α-glucosidase enzyme in our previous studies [50]. For modeling, protein sequence of *S. cerevisiae* α-glucosidase was retrieved from UniprotKB (accession number P53341) (https://www.uniprot.org/uniprot/P53341) and directly uploaded on SwissModel server (https://swissmodel.expasy.org/) which predicted 3A47 and 3AXH as good templates with more than 72% percent identity. These templates were selected, automatically aligned with target sequence and subjected to model building by SwissModel. At the end, five models were obtained and their stereochemical properties were assessed by Procheck-Ramachandran plot (http://services.mbi.ucla.edu/PROCHECK/), and geometrical properties were scrutinized by ERRAT (http://servicesn.mbi.ucla.edu/ERRAT/) and verify3D (http://servicesn.mbi.ucla.edu/Verify3D/). The best model was selected for docking studies. 

For docking, hydrogen atoms were added on protein structure and partial charges were calculated by Protonate 3D command of MOE. Finally each atom of protein was minimized by AMBER:EHT force field until the RMS gradient of 0.1 kcal/mol/Å was attained. The 3D coordinates of ligands were generated by MOE builder, hydrogen atoms were added, partial charges were calculated, and each molecule was minimized using MMF94x force field until the RMS gradient of 0.1 kcal/mol/Å was obtained. For docking, MOE alpha triangle placement method was chosen, along with London dG scoring function. Forcefield based GBVI/WSA dG method was applied for refinement and rescoring. After docking, thirty docked solutions of each ligand were saved for interaction analysis. For docking on human *Homo sapiens* α-glucosidase, X-ray coordinates of human α-glucosidase in complex with Acarbose (PDB code: 5NN8, resolution = 2.45Å) was retrieved and treated as discussed above for file preparation.

Additionally, the physicochemical and pharmacological profile of the compounds were predicted in silico by admetSAR (http://lmmd.ecust.edu.cn/admetsar2/) and SwissADME (http://www.swissadme.ch/)

### 3.6. Pharmacophore Modeling

Two receptor-based pharmacophore models were generated by MOE [46]. Initially *S. cerevisiae* α-glucosidase enzyme in complex with substrate molecule and an inhibitor molecule was uploaded separately on MOE in pdb form to generate models. For modeling, unified annotation scheme was used, which is the most comprehensive annotation types and includes all the terms like H-bond donor, H-bond acceptor, metal ligator, cation, anion, aromatic, and hydrophobic, etc. The pharmacophore models are shown in supporting information.

## 4. Conclusions

The bioassay-guided isolation approach resulted in the isolation of one new (ethyl methyl 2-bromobenzene 1,4-dioate, **1**), a new natural product (diethyl-2-bromobenzene 1,4-dioate, **2**) along with six known metabolites (**3**–**8**) from the methanolic extract of *D. hoytii.* To the best of our knowledge, this is the first comprehensive report on the isolation of biologically active secondary metabolites from *D. hoytii*. All the isolated compounds were reported for first time from this source. The results of the current investigation may suggest the potential of the selected marine seaweed as a source of novel bioactive natural products. This will provide a basis for the search for an alternative source of therapeutic agents, which can in turn be used as lead candidates for future drug development against diabetes mellitus.

## Figures and Tables

**Table 1:** α-Glucosidase inhibitory activities of compounds **1**–**8**.

Compounds	IC_50_ (μM)
**1**	522.0 ± 0.51
**2**	234.2 ± 4.18
**3**	289.4 ± 4.91
**4**	NA
**5**	ND
**6**	659.78 ± 2.15
**7**	30.5 ± 0.41
**8**	480.1 ± 2.11
Acarbose	942 ± 0.74

ND = not determined; NA = not active.

**Table 2:** Docking results of compounds **1**–**3**, **6**–**8**.

***S. cerevisiae* α-glucosidase**						
**Compounds**	**IC_50_ (μM)**	**Docking score**	**Protein–ligand interactions**			
			**Ligand**	**Receptor**	**Interactions**	**Distances (Å)**
**1**	522.0	−7.87	O12	NE2-HIS111	HBA	2.43
**2**	234.2	−8.47	O21 O12	NH1-ARG439 O-WAT1174	HBA HBA	1.95 1.97
**3**	289.4	−8.23	O69 O69 O69	OD1-ASP214 OD2-ASP214 NE2-HIS111	HBD HBD HBA	2.15 1.83 2.30
**6**	659.78	−5.33	O1 O7	O-PHE157 N-TYR313	HBD HBA	1.88 2.33
**7**	30.5	−10.62	O8 O8	OD1-ASP214 NE2-HIS111	HBD HBA	2.62 2.14
**8**	480.1	−8.18	O22 O24	NH2-ARG212 O-WAT1026	HBA HBA	2.09 1.96
Acarbose	942	−1.24	O17 O19 O21 O40 O42 O87 O19 O21 O21 O40 O44	OE1-GLU276 OD1-ASP214 OD1-ASP349 O-HOH1228 OD1-ASP408 OE2-GLU304 NH2-ARG212 NE2-HIS348 O-HOH1026 O-HOH1056 O-HOH1174	HBD HBD HBD HBD HBD HBD HBA HBA HBA HBA HBA	2.43 2.79 3.27 2.53 2.99 2.96 3.47 3.02 2.71 2.42 2.56
***H. Sapiens* α-glucosidase**						
**Compounds**	**IC_50_ (μM)**	**Docking score**	**Protein–ligand interactions**			
			**Ligand**	**Receptor**	**Interactions**	**Distances (Å)**
**1**	ND	−6.41	O12 O21 O23	NH1-ARG600 NE2-HIS674 NE2-HIS674	HBA HBA HBA	1.95 2.44 1.91
**2**	ND	−6.44	O12 O21	NH1-ARG600 NE2-HIS674	HBA HBA	2.03 1.95
**3**	ND	−7.07	O69 O69 O69	OD1-ASP616 OD2-ASP616 NH1-ARG600	HBD HBD HBA	1.94 2.22 2.84
**6**	ND	−6.26	O1	OD2-ASP518	HBD	1.85
**7**	ND	−9.08	O8 O10	OD1-ASP404 O-Wat1106	HBD HBA	2.00 3.01
**8**	ND	−7.23	O22 O24	OD2-ASP518 NE2-HIS674	HBD HBA	1.94 3.14
Acarbose	ND	−9.65	O15 O17 O19 O21 N37 O40 O42 O42 O15 O19 O42 O64 O87 N37 N37	OD1-ASP616 O-HOH1164 OD2-ASP404 OD1-ASP404 OD2-ASP616 OD1-ASP282 OD2-ASP282 SD-MET519 NH1-ARG600 NE2-HIS674 NH1-ARG600 N-ALA284 O-HOH1276 OD1-ASP616 OD2-ASP616	HBD HBD HBD HBD HBD HBD HBD HBD HBA HBA HBA HBA HBA IONIC IONIC	2.60 2.84 2.90 2.86 2.75 3.12 2.78 3.21 3.05 3.38 3.08 3.06 3.14 3.36 2.75

**Table 3:** Predicted ADMET properties of compounds **1**–**3** and **6**–**8**.

Compounds	Pharmacokinetic Properties
**1**	HIA = High, Caco-2 = Good, BBB = Yes, HOB = Yes, P-glycoprotein inh/subs = No, Carcinogenicity = No, Ames mutagenesis = No, Hepatotoxicity = No, Acute Oral Toxicity = III (2.28 kg/mol), Biodegradation = Yes, Log *K*_p_ = −6.14 cm/s, CYP3A4 subs = No, CYP2C9 subs = No, CYP2D6 subs = No, CYP3A4 inh = No, CYP2C9 inh = No, CYP2C19 inh = Yes, CYP2D6 inh = Yes, CYP1A2 inh = Yes, CYP inhibitory promiscuity = No
**2**	HIA = High, Caco-2 = Good, BBB = Yes, HOB = Yes, P-glycoprotein inh/subs = No, Carcinogenicity = No, Ames mutagenesis = No, Hepatotoxicity = No, Acute Oral Toxicity = III (1.695 kg/mol), Biodegradation = No, Log *K*_p_ = −5.97 cm/s, CYP3A4 subs = No, CYP2C9 subs = No, CYP2D6 subs = No, CYP3A4 inh = No, CYP2C9 inh = No, CYP2C19 inh = No, CYP2D6 inh = No, CYP1A2 inh = Yes, CYP inhibitory promiscuity = No
**3**	HIA = Low, Caco-2 = Good, BBB = No, HOB = No, P-glycoprotein inh/subs = No, Carcinogenicity = No, Ames mutagenesis = No, Hepatotoxicity = No, Acute Oral Toxicity = I (3.471 kg/mol), Biodegradation = No, Log *K*_p_ = −2.53 cm/s, CYP3A4 subs = Yes, CYP2C9 subs = No, CYP2D6 subs = No, CYP3A4 inh = No, CYP2C9 inh = No, CYP2C19 inh = No, CYP2D6 inh = No, CYP1A2 inh = No, CYP inhibitory promiscuity = No
**6**	HIA = Low, Caco-2 = No, BBB = No, HOB = No, P-glycoprotein inh/subs = No, Carcinogenicity = No, Ames mutagenesis = No, Hepatotoxicity = No, Acute Oral Toxicity = IV (1.499kg/mol), Biodegradation = Yes, Log *K*_p_ = −0.39cm/s, CYP3A4 subs = Yes, CYP2C9 subs = No, CYP2D6 subs = No, CYP3A4 inh = No, CYP2C9 inh = No, CYP2C19 inh = No, CYP2D6 inh = No, CYP1A2 inh = No, CYP inhibitory promiscuity = No
**7**	HIA = Low, Caco-2 = No, BBB = No, HOB = No, P-glycoprotein inh/subs = No, Carcinogenicity = No, Ames mutagenesis = No, Hepatotoxicity = No, Acute Oral Toxicity = IV (1.078kg/mol), Biodegradation = Yes, Log *K*_p_ = −0.11cm/s, CYP3A4 subs = No, CYP2C9 subs = Yes, CYP2D6 subs = No, CYP3A4 inh = No, CYP2C9 inh = No, CYP2C19 inh = No, CYP2D6 inh = No, CYP1A2 inh = Yes, CYP inhibitory promiscuity = No
**8**	HIA = High, Caco-2 = Good, BBB = Yes, HOB = No, P-glycoprotein inh/subs = No, Carcinogenicity = No, Ames mutagenesis = No, Hepatotoxicity = No, Acute Oral Toxicity = IV (1.699kg/mol), Biodegradation = Yes, Log *K*_p_ = −2.90cm/s, CYP3A4 subs = No, CYP2C9 subs = Yes, CYP2D6 subs = No, CYP3A4 inh = No, CYP2C9 inh = No, CYP2C19 inh = No, CYP2D6 inh = No, CYP1A2 inh = Yes, CYP inhibitory promiscuity = No

HIA = Human Intestinal Absorption, Caco-2 = absorption in human epithelial colorectal adenocarcinoma cell lines, BBB = Blood brain barrier, HOB = Human oral bioavailability, Inh = inhibitor, Subs = substrate, Log *K*_p_ = Skin permeation, CYP = Cytochrome p450.

## Supplementary Materials

The following are available online at https://www.mdpi.com/1660-3397/17/12/666/s1. NMR and MS data for compounds **1**–**8** is included. This material is available free of charge via MDPI website.
