# Supplementary material for: α-Glucosidase Inhibition and Molecular Docking Studies of Natural Brominated Metabolites from Marine Macro Brown Alga Dictyopteris hoytii
Source: Mar Drugs. 2019 Nov 26;17(12):666. doi: 10.3390/md17120666 (PMC6949951; doi:10.3390/md17120666)
Supplement: Supplementary file 1 [file marinedrugs-17-00666-s001.pdf]

Supplementary

α-Glucosidase Inhibition and Molecular Docking Studies of Natural Brominated Metabolites from Marine Macro Brown Alga *Dictyopteris hoytii*

Najeeb Ur Rehman [1], Kashif Rafiq [1,2], Ajmal Khan [1], Sobia Ahsan Halim [1], Liaqat Ali [1,3], Nadiya Al-Saady [4], Abdullah Hilal Al-Balushi [4], Haitham Khamis Al-Busaidi [4] and Ahmed Al-Harrasi [1,*]

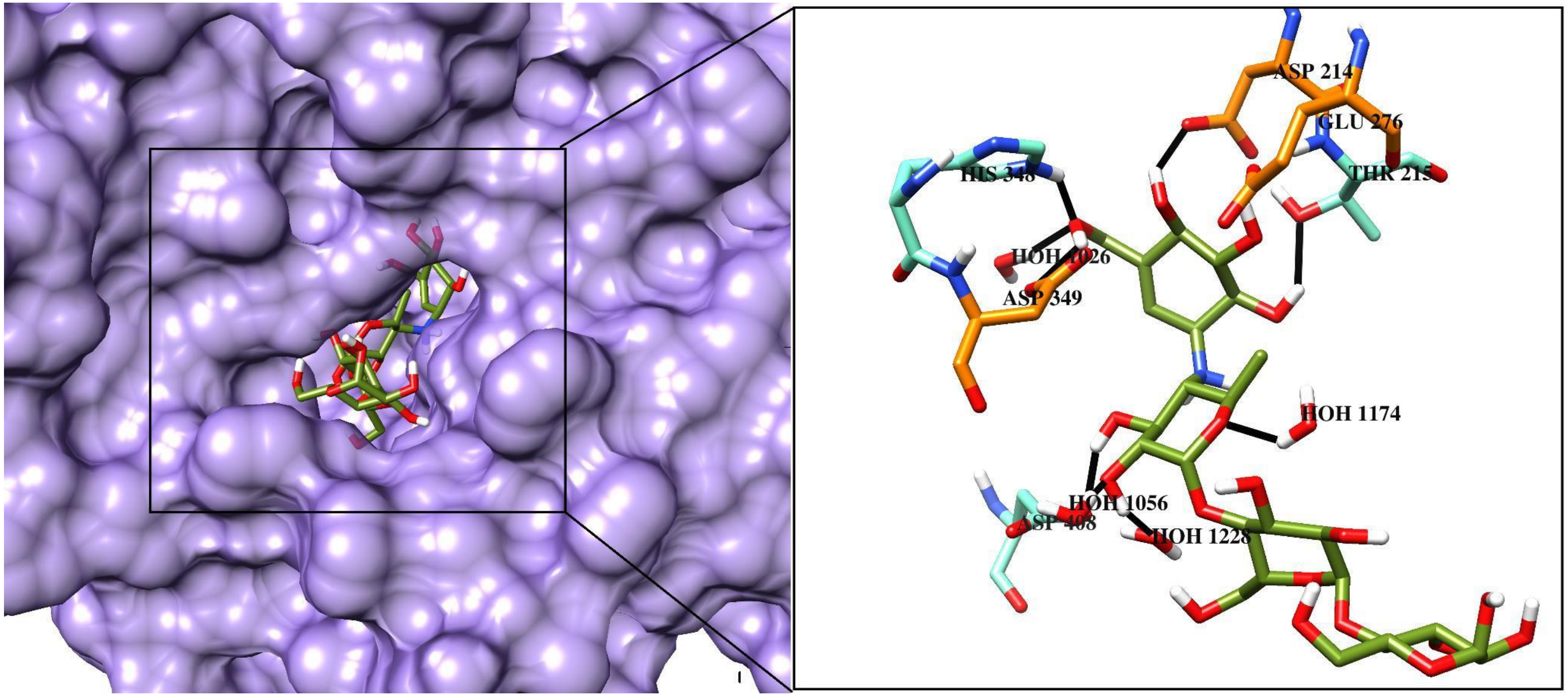

Figure S1. Docked view of known inhibitor Acarbose in the active site of *S. cerevisiae* α-glucosidase enzyme. The binding interactions are highlighted in box. Catalytic residues are shown in orange stick model, Hydrogen bonds are depicted in black lines. Acarbose is shown in olive green stick model.

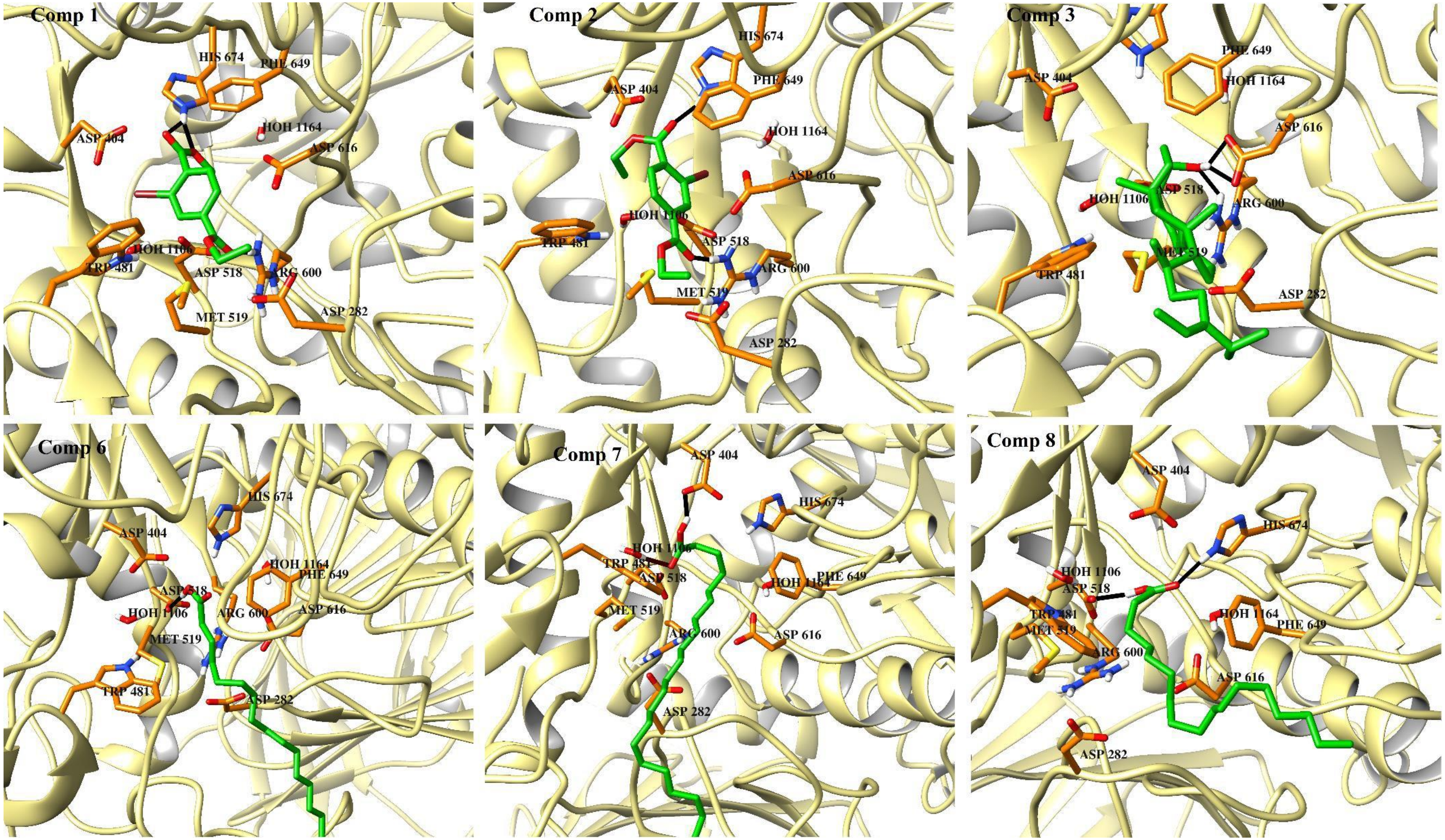

Figure S2. Docked view of compounds 1-3 and 6-8 in the active site of human α-glucosidase enzyme. The ligands are shown in green stick model, interacting residues are shown in orange stick model, and Hydrogen bonds are depicted in black lines.

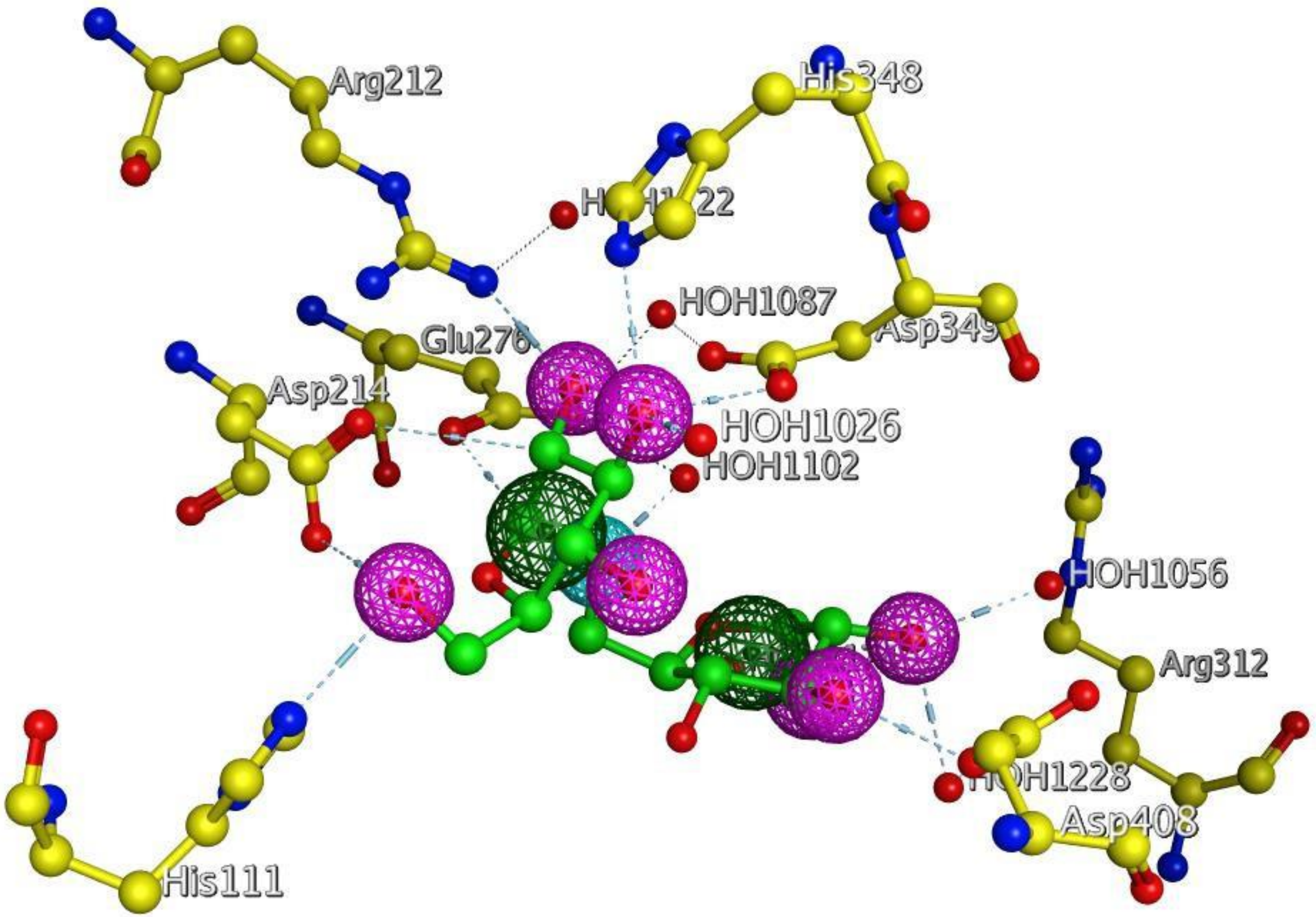

Figure S3. Structure-based pharmacophore model (called Ph-1) generated by S. cerevisiae α-glucosidase enzyme in complex with isomaltose (substrate). Substrate in shown in green stick model, interacting residues are shown in yellow stick model. Water molecules are shown in red ball. Hydrogen bonds are depicted in cyan dotted lines. Seven H-bond donor/Acceptor (Don/Acc) features are presented in magenta spheres. Hydrophobic features are shown in dark green spheres. One H-bond Acceptor feature is presented in cyan (Acc) sphere.

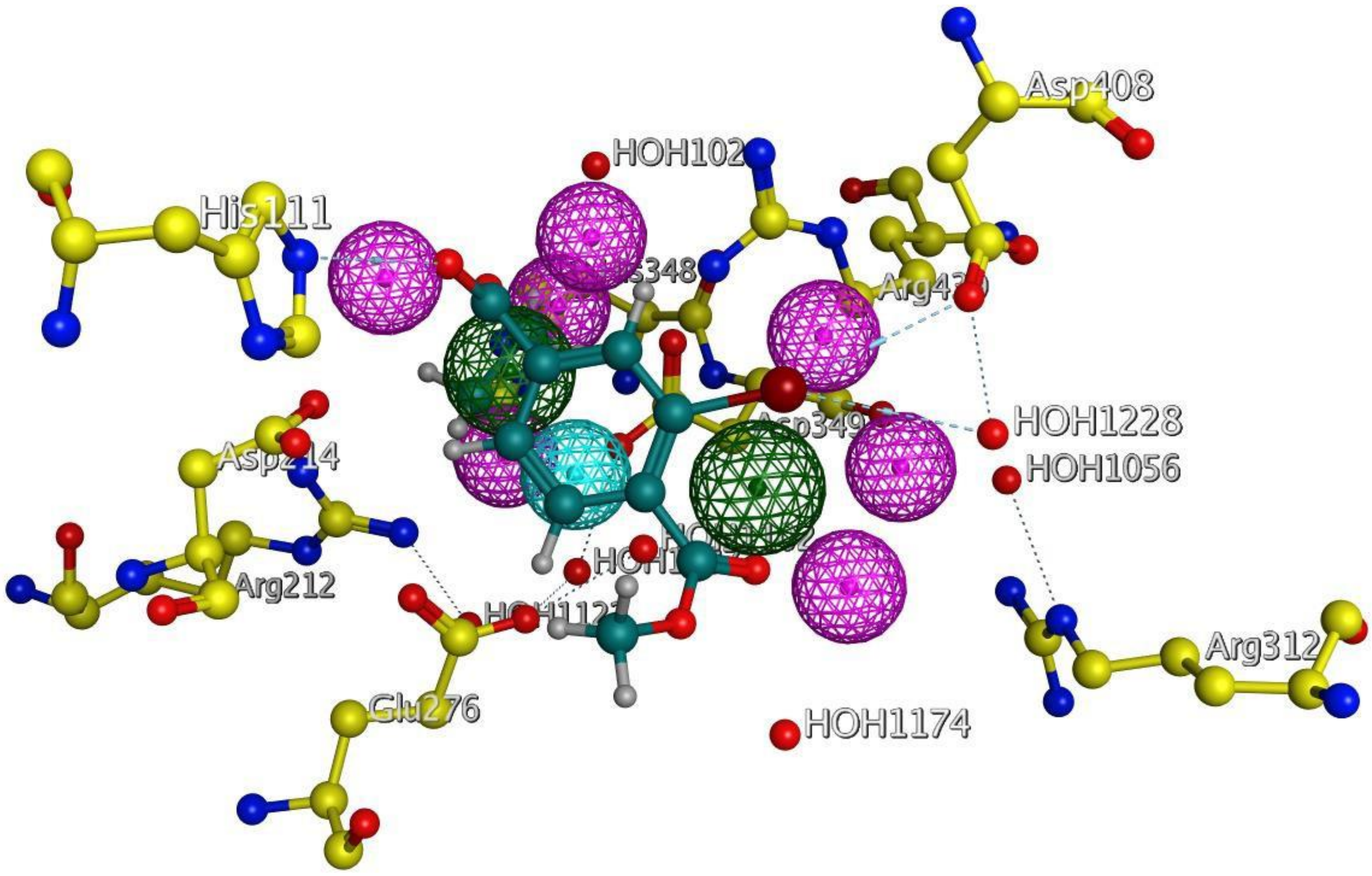

Figure S4. Superimposed view of Compound **1** on Ph-1. Ligand is shown in green stick model, interacting residues are shown in yellow stick model. Water molecules are shown in red ball. Hydrogen bonds are depicted in cyan dotted lines. H-bond Don/Acc Hydrophobic features, H-bond Acceptor feature are presented in magenta spheres, dark green spheres and cyan sphere, respectively

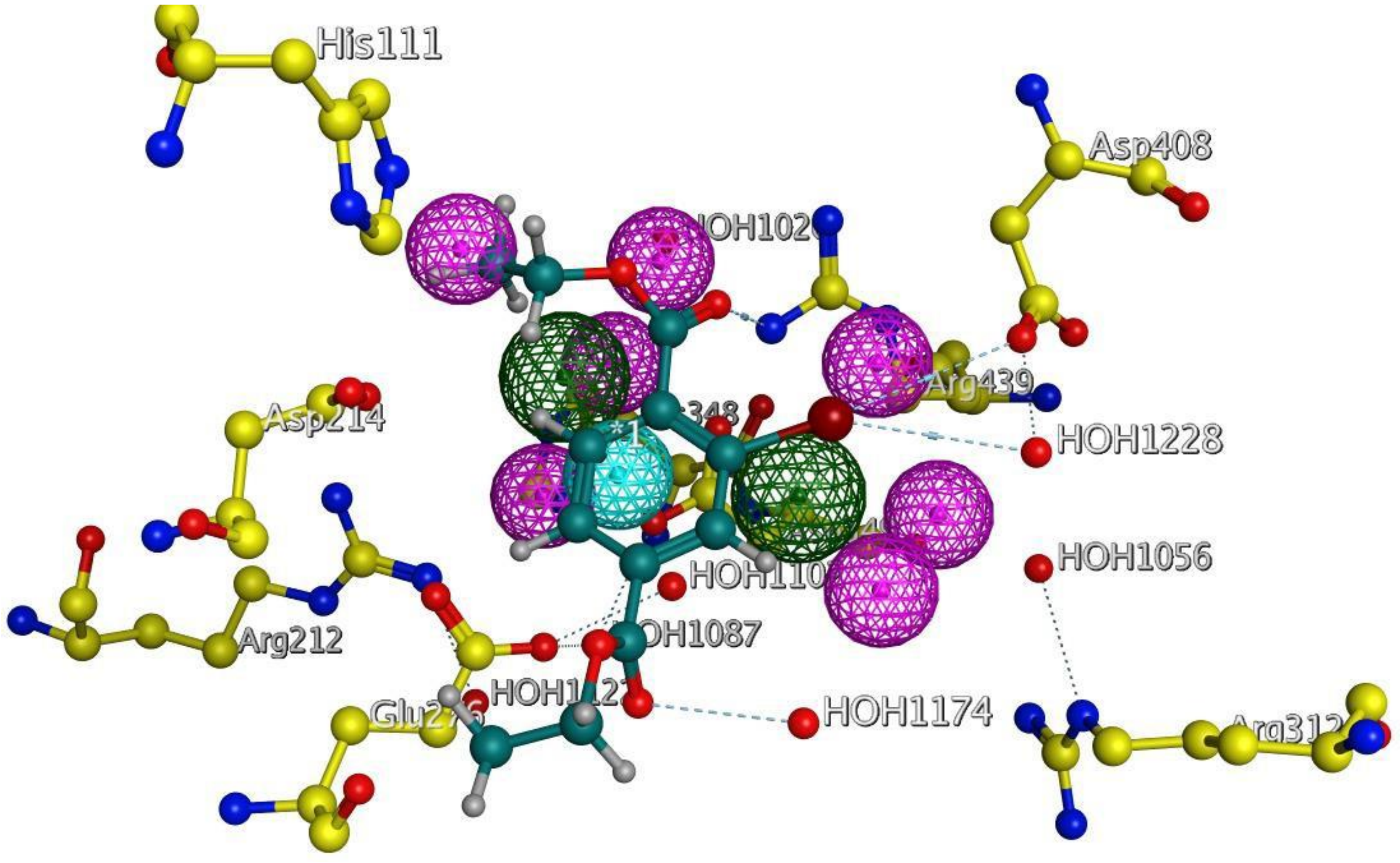

Figure S5. Superimposed view of Compound **2** on Ph-1. Ligand is shown in green stick model, interacting residues are shown in yellow stick model. Water molecules are shown in red ball. Hydrogen bonds are depicted in cyan dotted lines. H-bond Don/Acc Hydrophobic features, H-bond Acceptor feature are presented in magenta spheres, dark green spheres and cyan sphere, respectively

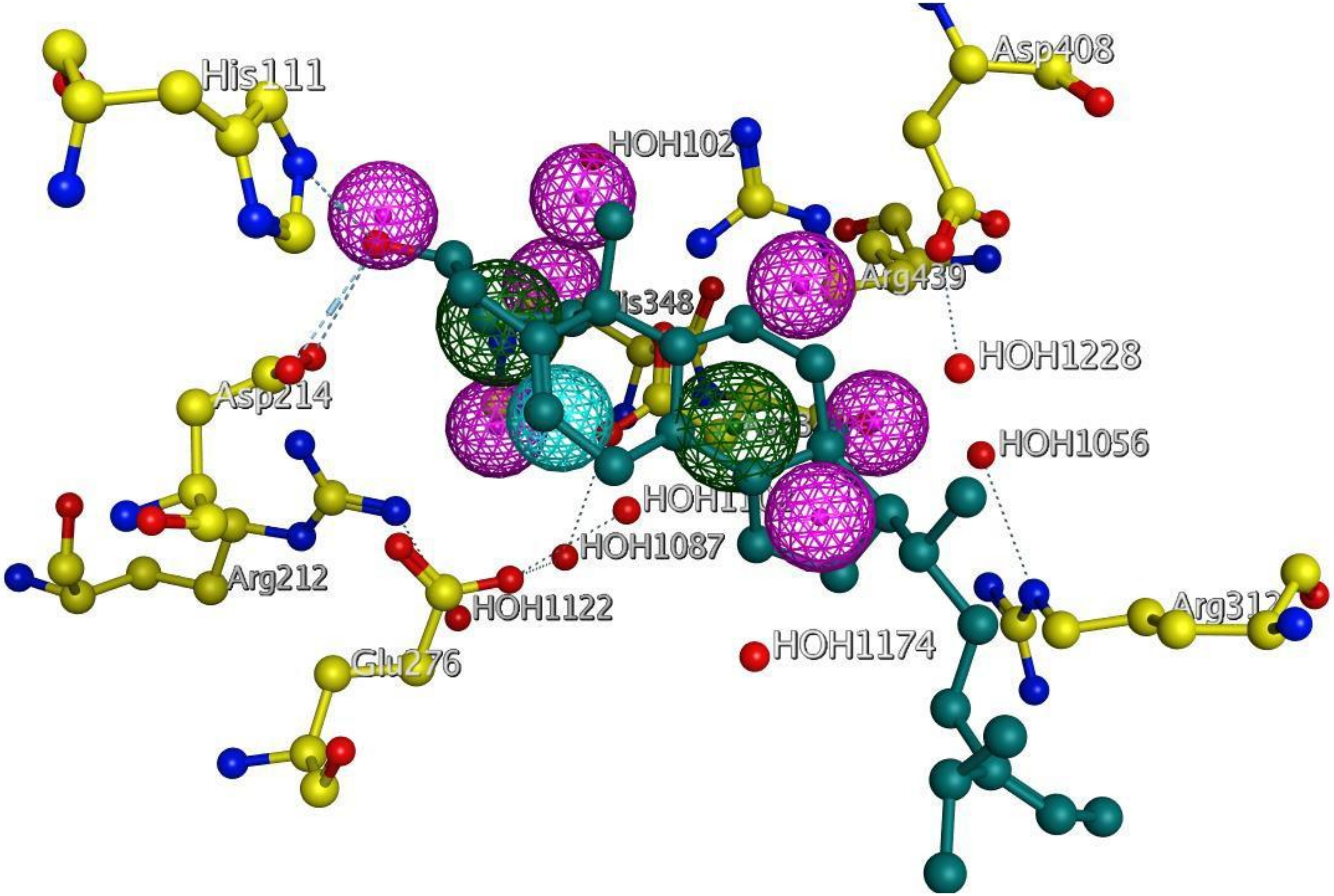

Figure S6. Superimposed view of Compound **3** on Ph-1. Ligand is shown in green stick model, interacting residues are shown in yellow stick model. Water molecules are shown in red ball. Hydrogen bonds are depicted in cyan dotted lines. H-bond Don/Acc Hydrophobic features, H-bond Acceptor feature are presented in magenta spheres, dark green spheres and cyan sphere, respectively

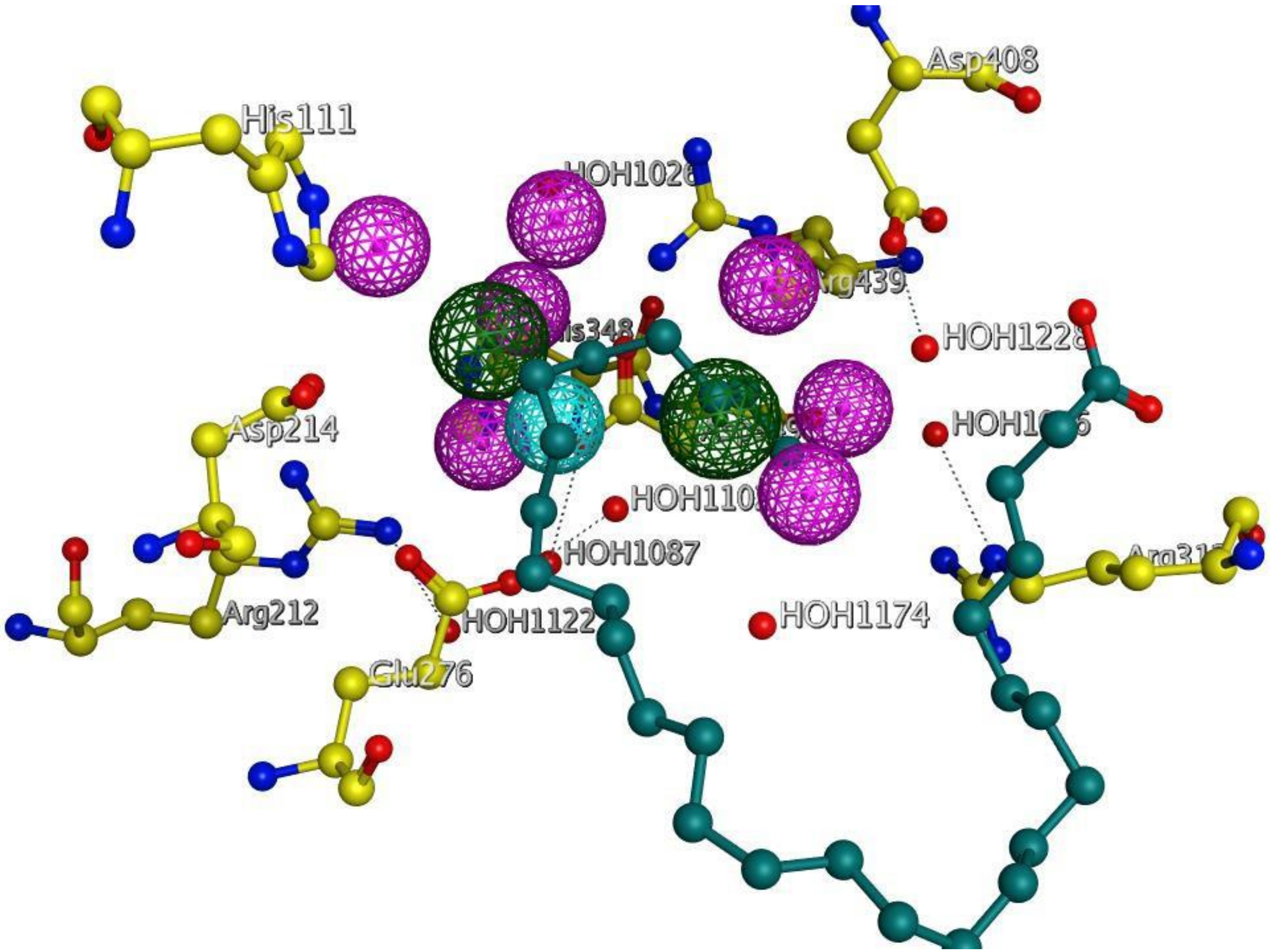

Figure S7. Superimposed view of Compound **6** on Ph-1. Ligand is shown in green stick model, interacting residues are shown in yellow stick model. Water molecules are shown in red ball. Hydrogen bonds are depicted in cyan dotted lines. H-bond Don/Acc Hydrophobic features, H-bond Acceptor feature are presented in magenta spheres, dark green spheres and cyan sphere, respectively

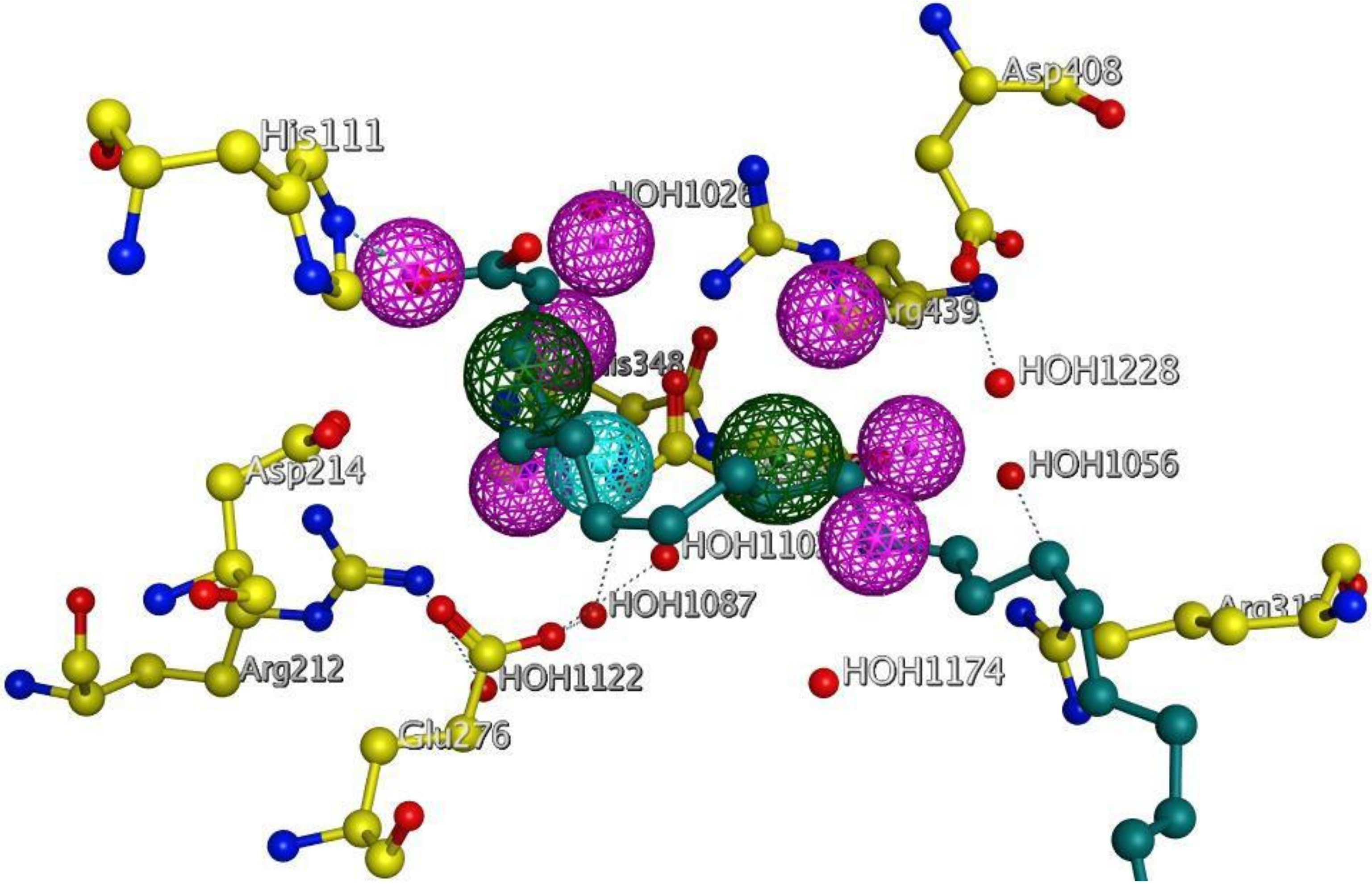

Figure S8. Superimposed view of Compound **7** on Ph-1. Ligand is shown in green stick model, interacting residues are shown in yellow stick model. Water molecules are shown in red ball. Hydrogen bonds are depicted in cyan dotted lines. H-bond Don/Acc Hydrophobic features, H-bond Acceptor feature are presented in magenta spheres, dark green spheres and cyan sphere, respectively

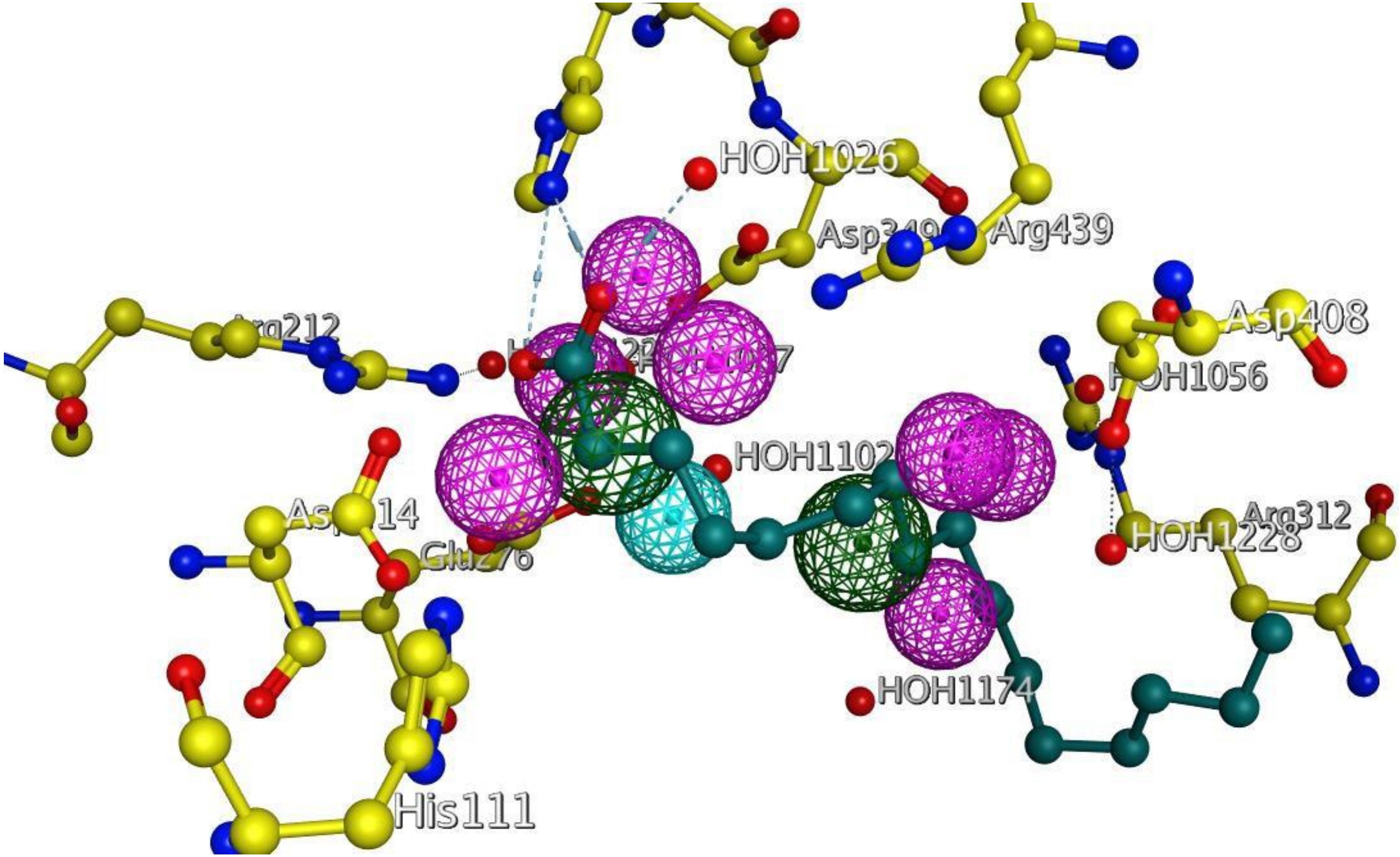

Figure S9. Superimposed view of Compound **8** on Ph-1. Ligand is shown in green stick model, interacting residues are shown in yellow stick model. Water molecules are shown in red ball. Hydrogen bonds are depicted in cyan dotted lines. H-bond Don/Acc Hydrophobic features, H-bond Acceptor feature are presented in magenta spheres, dark green spheres and cyan sphere, respectively

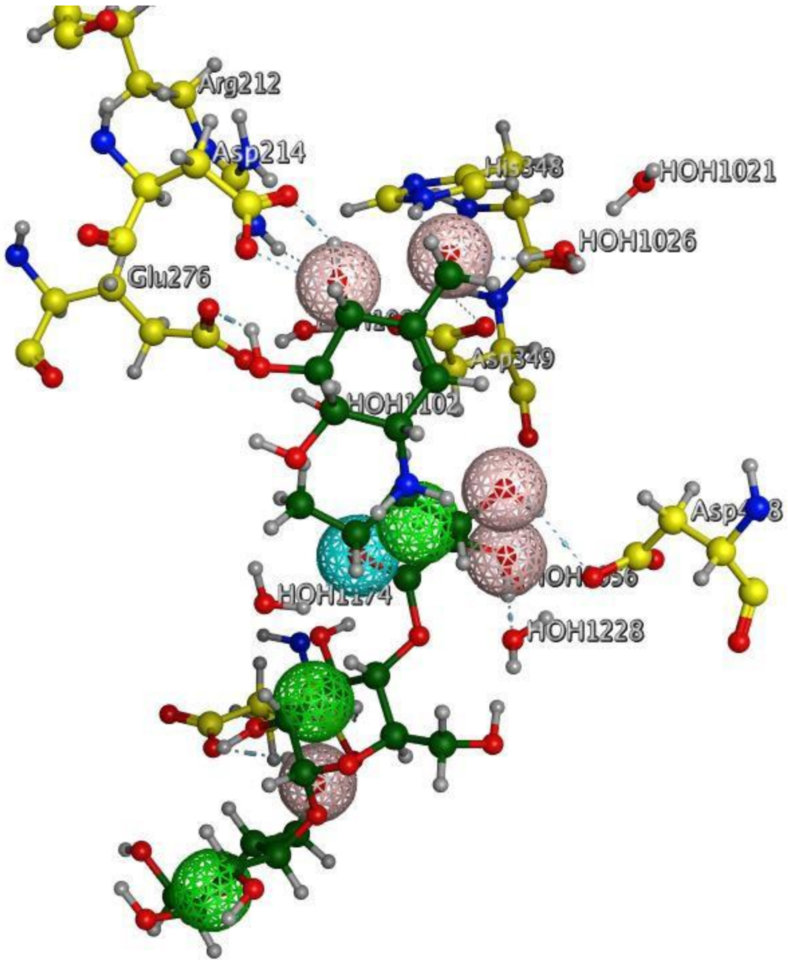

Figure S10. Structure-based pharmacophore model (called Ph-2) generated by S. cerevisiae α-glucosidase enzyme in complex with Acarbose (inhibitor). Acarbose is shown in green stick model, interacting residues are shown in yellow stick model. Water molecules are shown in red ball. Hydrogen bonds are depicted in cyan dotted lines. Five H-bond donor/Acceptor (Don/Acc) features are presented in light pink spheres. Hydrophobic features are shown in dark green spheres. One H-bond Acceptor (Acc) feature is presented in cyan sphere.

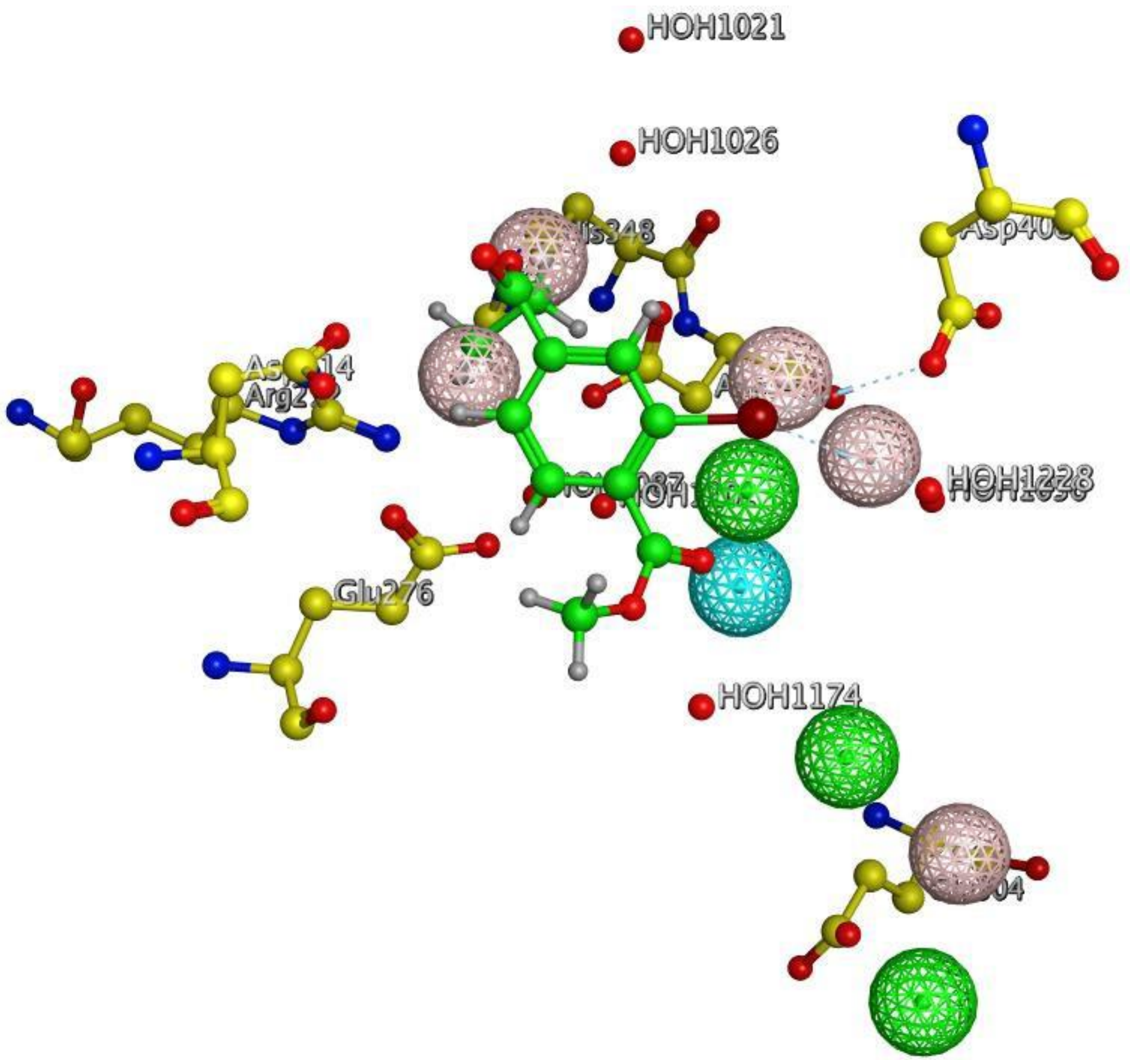

Figure S11. Superimposed view of Compound **1** on Ph-2. Ligand is shown in green stick model, interacting residues are shown in yellow stick model. Water molecules are shown in red ball. Hydrogen bonds are depicted in cyan dotted lines. H-bond Don/Acc Hydrophobic features, H-bond Acceptor feature are presented in light pink spheres, dark green spheres and cyan sphere, respectively

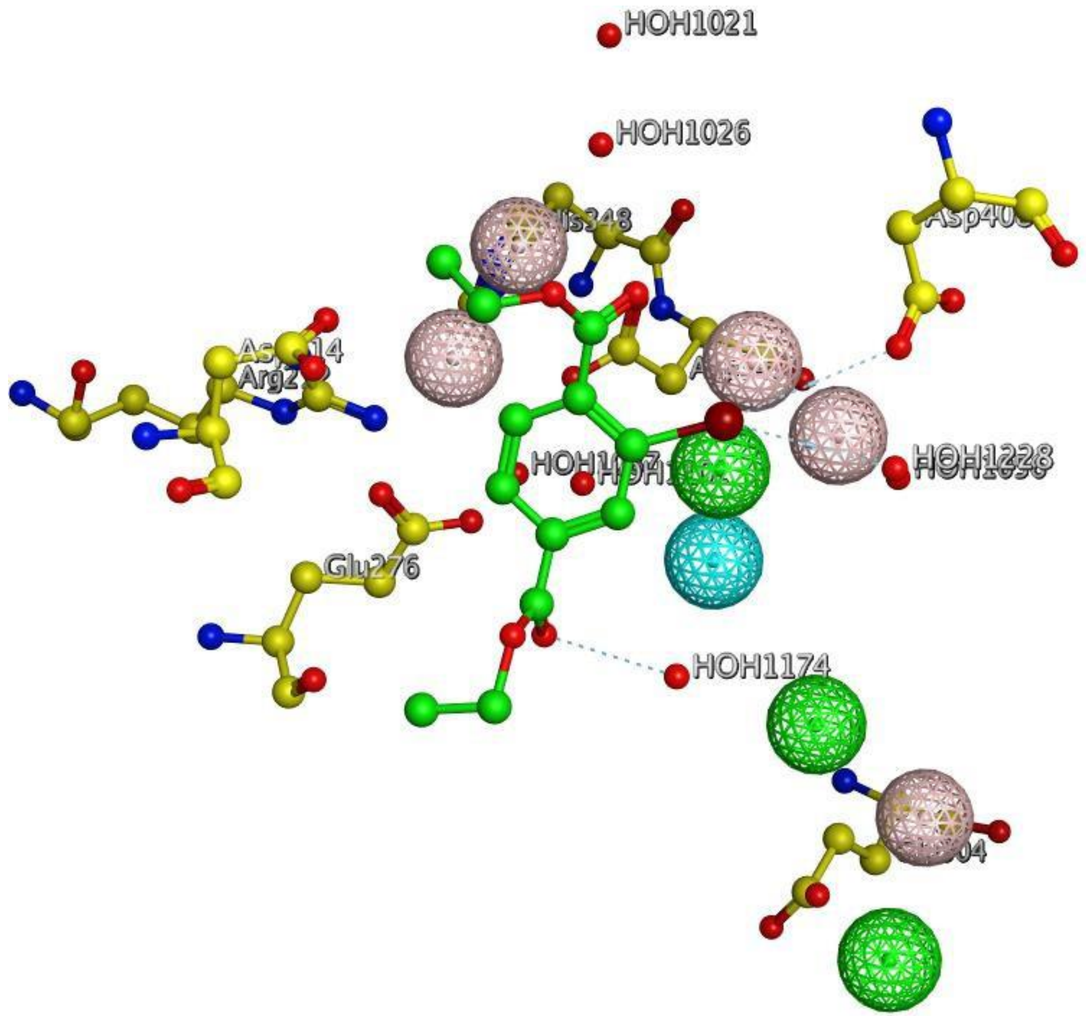

Figure S12. Superimposed view of Compound **2** on Ph-2. Ligand is shown in green stick model, interacting residues are shown in yellow stick model. Water molecules are shown in red ball. Hydrogen bonds are depicted in cyan dotted lines. H-bond Don/Acc Hydrophobic features, H-bond Acceptor feature are presented in light pink spheres, dark green spheres and cyan sphere, respectively

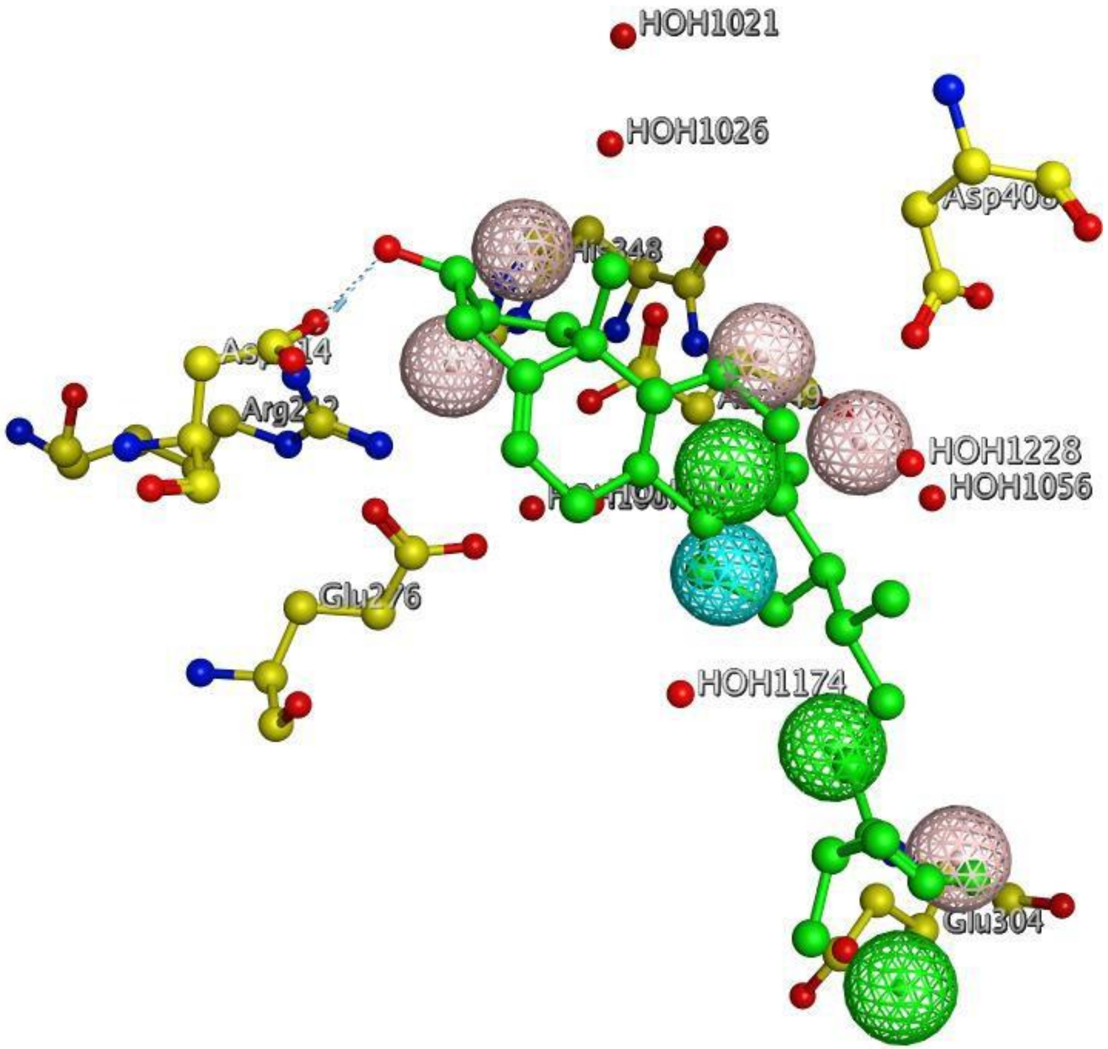

Figure S13 Superimposed view of Compound **3** on Ph-2. Ligand is shown in green stick model, interacting residues are shown in yellow stick model. Water molecules are shown in red ball. Hydrogen bonds are depicted in cyan dotted lines. H-bond Don/Acc Hydrophobic features, H-bond Acceptor feature are presented in light pink spheres, dark green spheres and cyan sphere, respectively

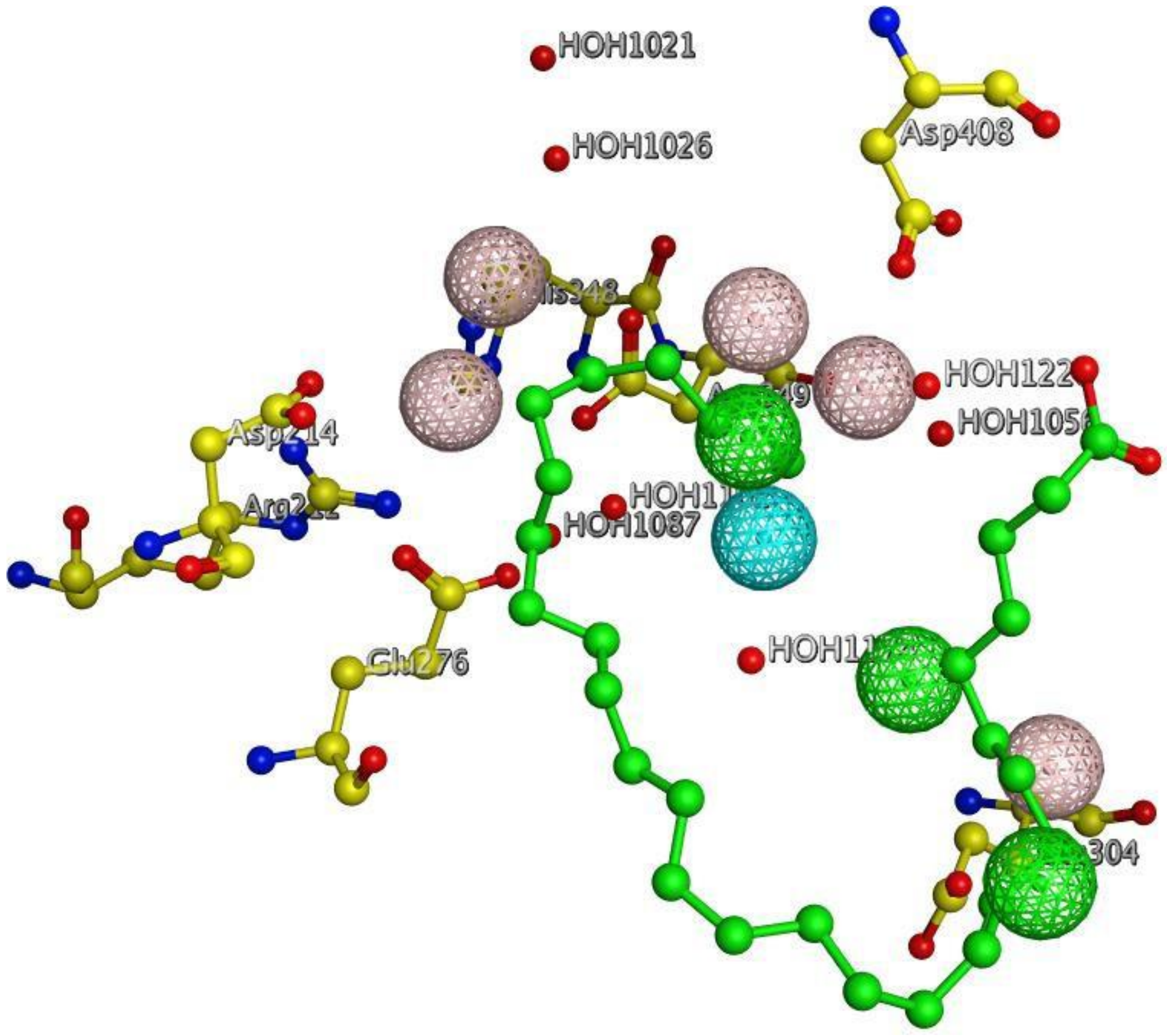

Figure S14. Superimposed view of Compound **6** on Ph-2. Ligand is shown in green stick model, interacting residues are shown in yellow stick model. Water molecules are shown in red ball. Hydrogen bonds are depicted in cyan dotted lines. H-bond Don/Acc Hydrophobic features, H-bond Acceptor feature are presented in light pink spheres, dark green spheres and cyan sphere, respectively

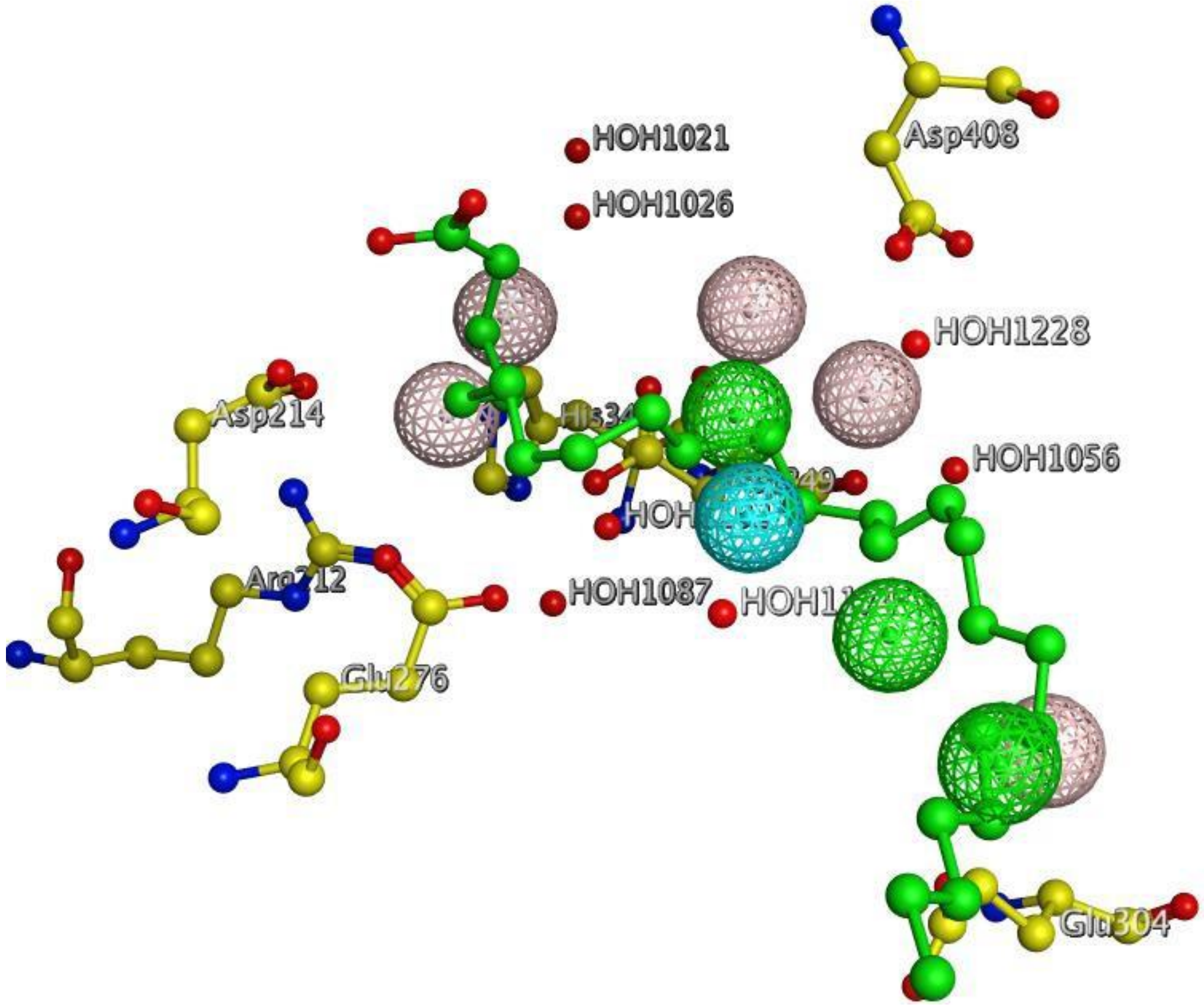

Figure S15. Superimposed view of Compound **7** on Ph-2. Ligand is shown in green stick model, interacting residues are shown in yellow stick model. Water molecules are shown in red ball. Hydrogen bonds are depicted in cyan dotted lines. H-bond Don/Acc Hydrophobic features, H-bond Acceptor feature are presented in light pink spheres, dark green spheres and cyan sphere, respectively

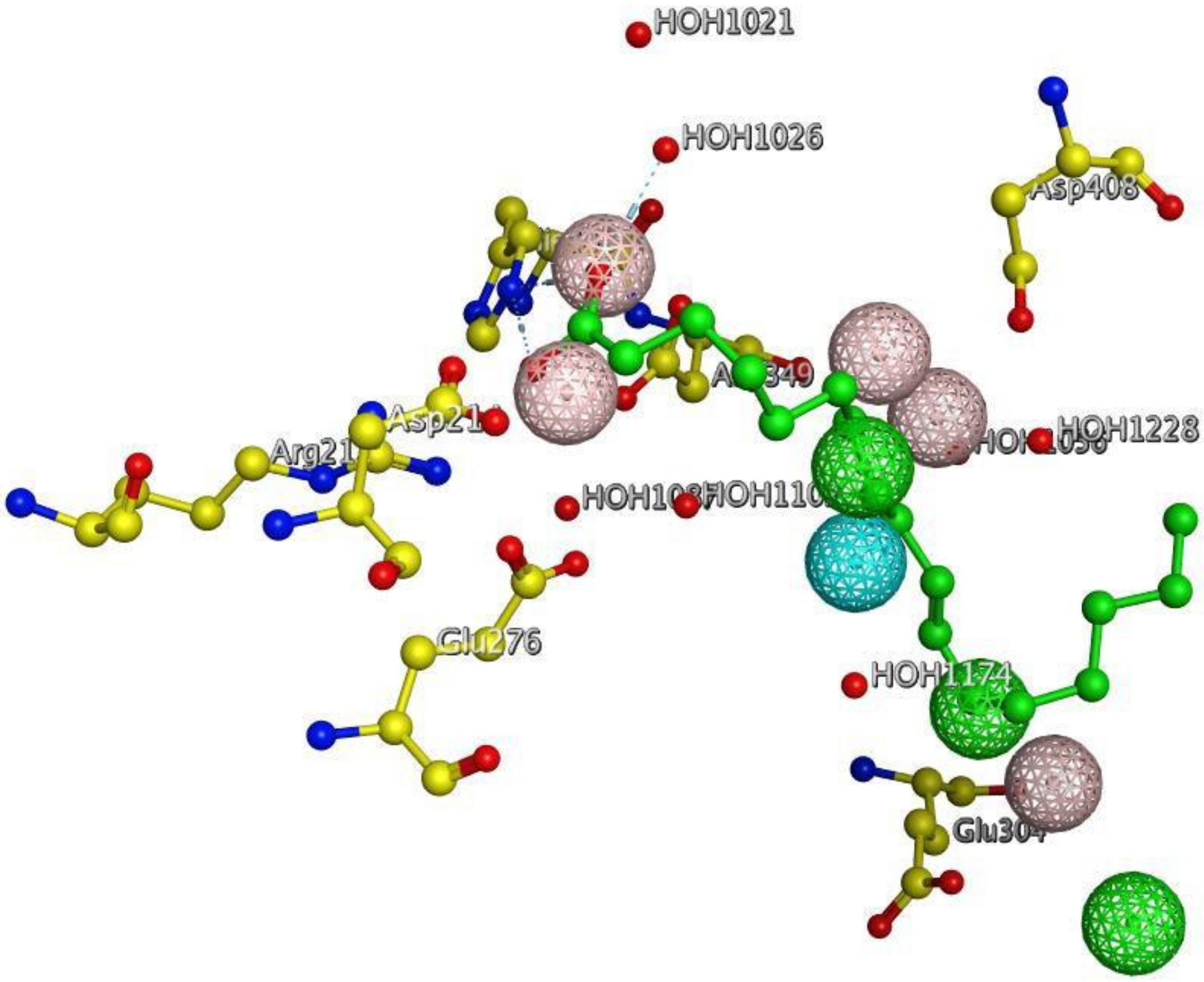

Figure S16. Superimposed view of Compound **8** on Ph-2. Ligand is shown in green stick model, interacting residues are shown in yellow stick model. Water molecules are shown in red ball. Hydrogen bonds are depicted in cyan dotted lines. H-bond Don/Acc Hydrophobic features, H-bond Acceptor feature are presented in light pink spheres, dark green spheres and cyan sphere, respectively.

NMR Spectra and HRMS of the Isolated Compounds

Figure S17. 1H-NMR (600 MHz, CDCl3) of compound **1**

Figure S18. 13C-NMR (150 MHz, CDCl3) Spectrum of Compound **1**

Figure S19. DEPT 135 of compound **1**

Figure S20. DEPT 90 of compound **1**

Figure S20. HRMS (ESI+) of compound **1**

Figure S22. 1H-NMR (600 MHz, CDCl3) of compound **2**

Figure S23. 13C-NMR (150 MHz, CDCl3) of compound **2**

Figure S24. DEPT 135 of compound **2**

Figure S25. DEPT 90 of compound **2**

Figure S26. HRMS (ESI+) of compound **2**

Figure S27. 1H-NMR (600 MHz, CDCl3) of compound **3**

Figure S28. 13C-NMR (150 MHz, CDCl3) of compound **3**

Figure S29. HRMS (ESI+) of compound **3**

Figure S30. 1H-NMR (600 MHz, CDCl3) of compound **4**

Figure S31. 13C-NMR (150 MHz, CDCl3) of compound **4**

Figure S32. HRMS (ESI+) of compound **4**

Figure S33. 1H-NMR (600 MHz, CDCl3) of compound **5**

Figure S34. 13C-NMR (150 MHz, CDCl3) of compound **5**

Figure S35. HRMS (ESI+) of compound **5**

Figure S36. 1H-NMR (600 MHz, CDCl3) of compound **6**

Figure S37. 13C-NMR (150 MHz, CDCl3) of compound **6**

Figure S38. HRMS (ESI+) of compound **6**

Figure S39. 1H-NMR (600 MHz, CDCl3) of compound **7**

Figure S40. 13C-NMR (150 MHz, CDCl3) of compound **7**

Figure S41. HRMS (ESI+) of compound **7**

Figure S42. 1H-NMR (600 MHz, CDCl3) of compound **8**

Figure S43. 13C-NMR (150 MHz, CDCl3) of compound **8**

Figure S44. HRMS (ESI+) of compound **8**

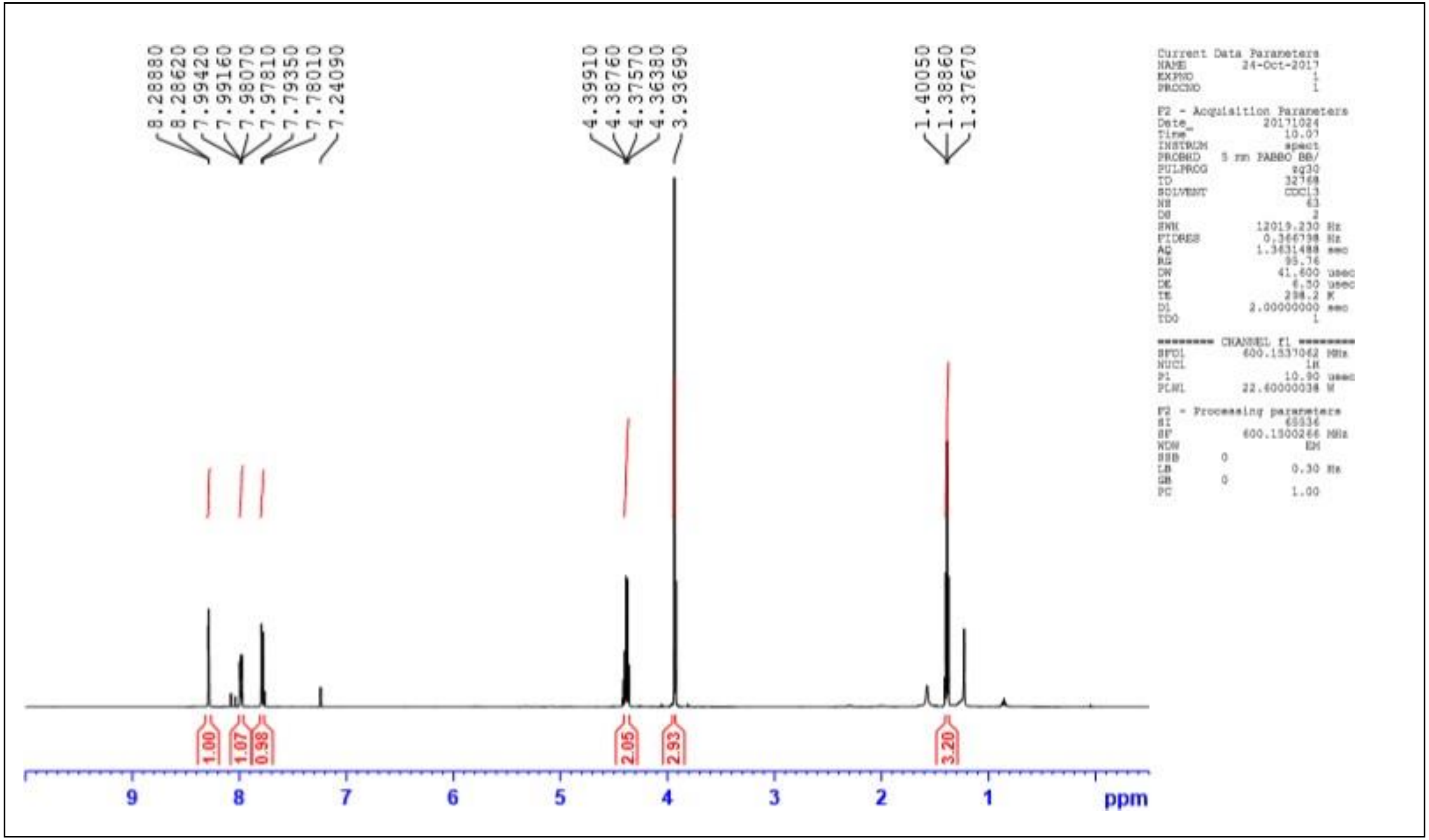

Figure S17. ^{1}H-NMR (600 MHz, $CDCl_3$) of compound **1**

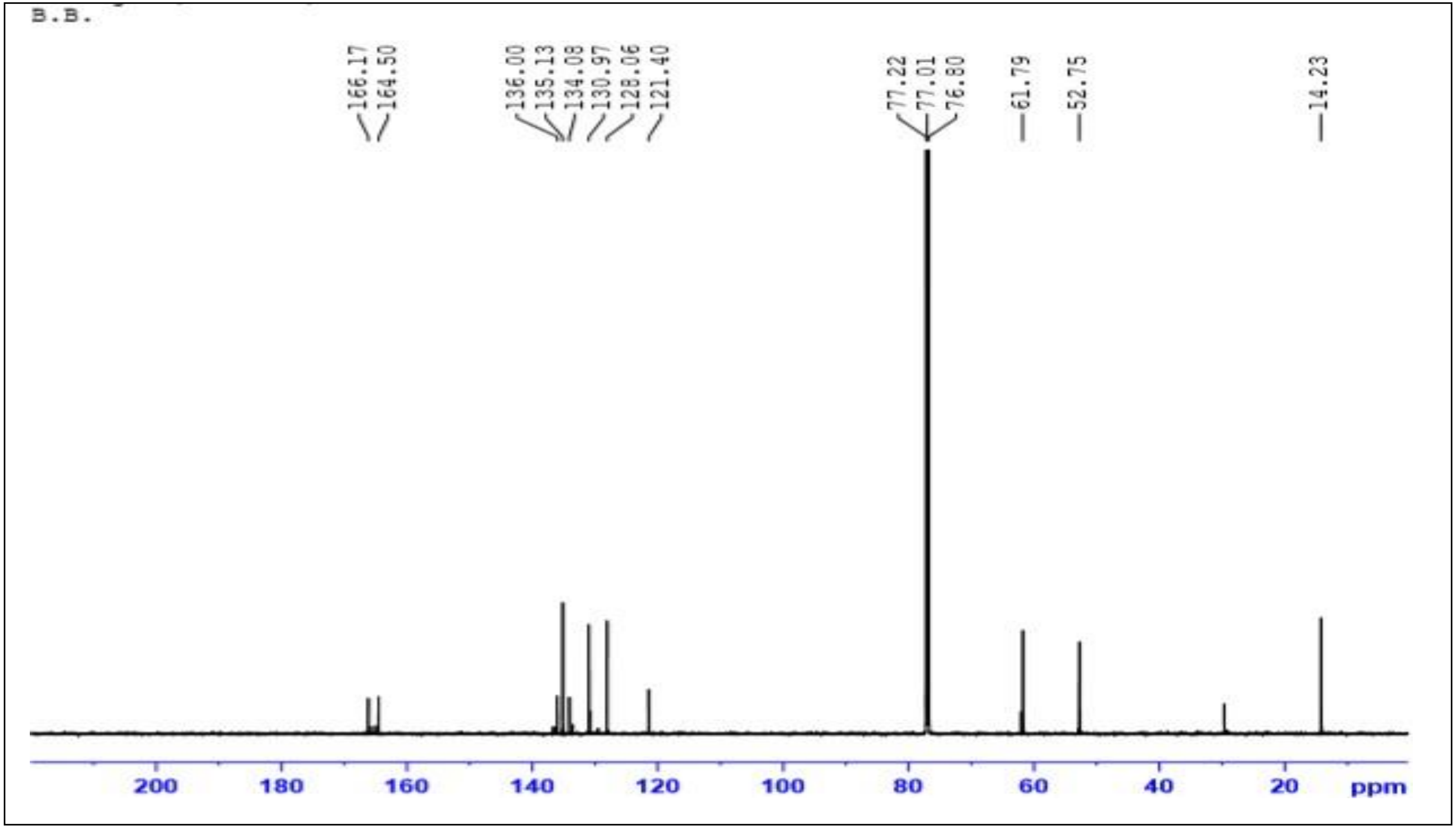

Figure S18. ^{13}C-NMR (150 MHz, $CDCl_3$) of compound **1**

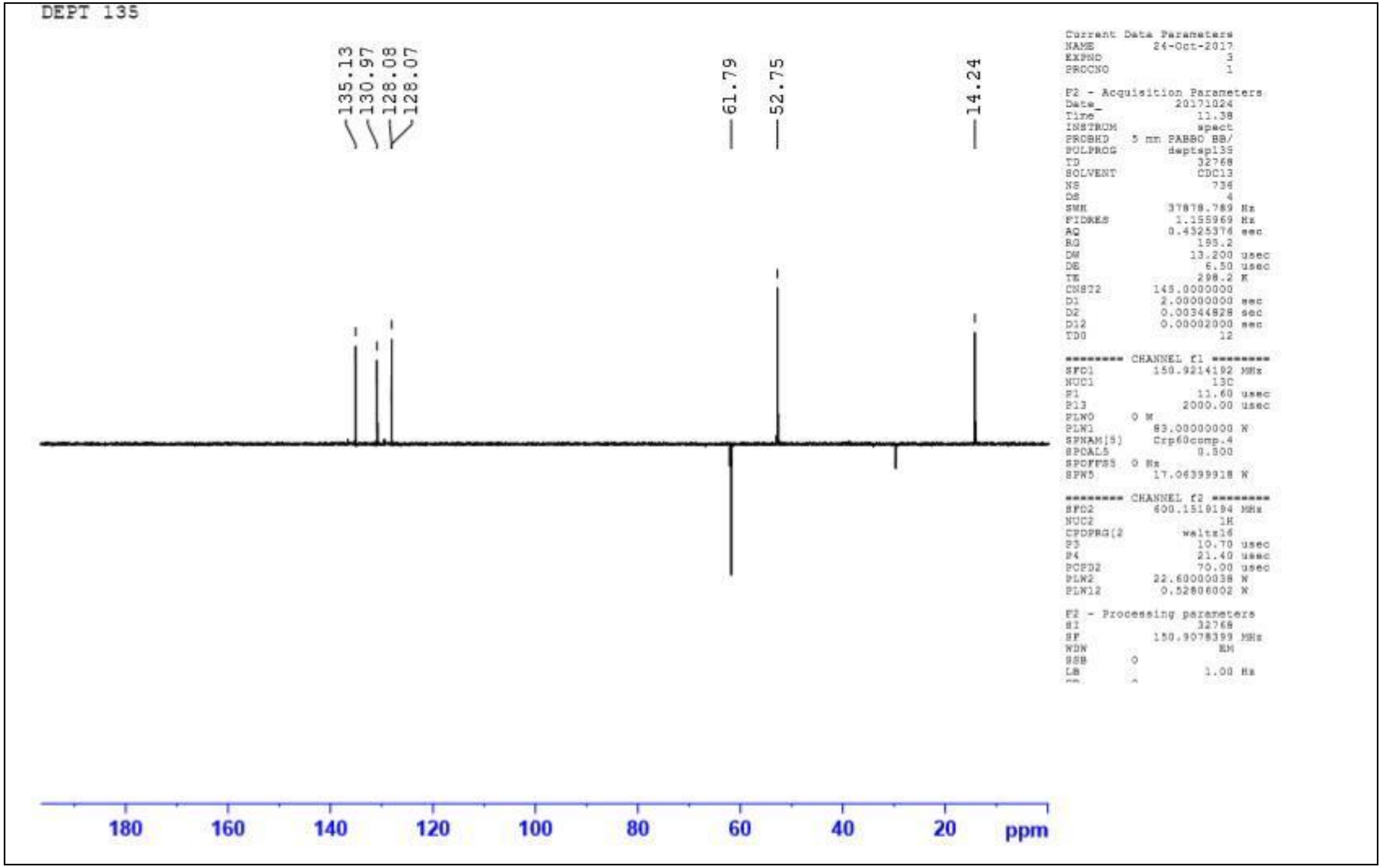

Figure S19. DEPT 135 of compound **1**

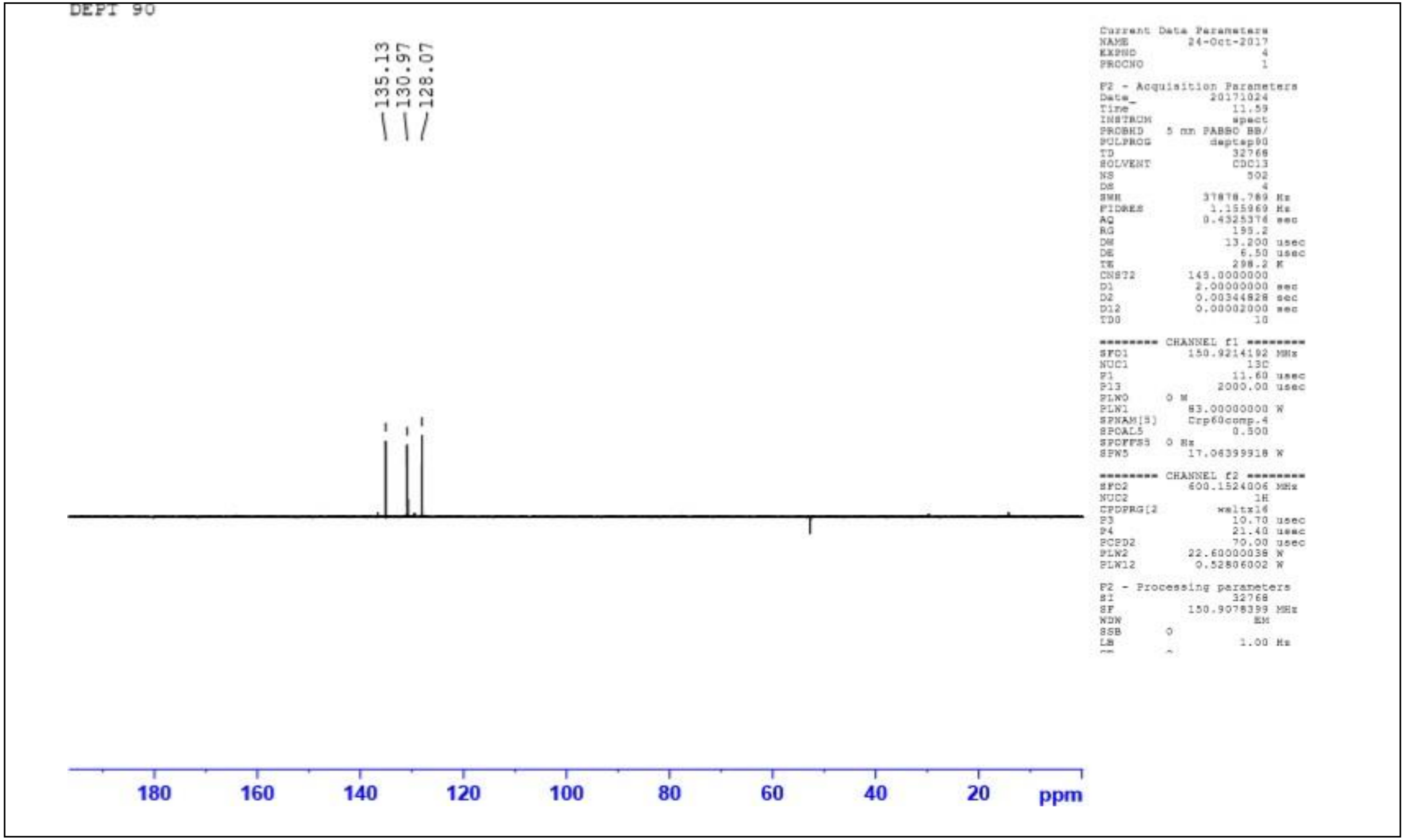

Figure S20. DEPT 90 of compound **1**

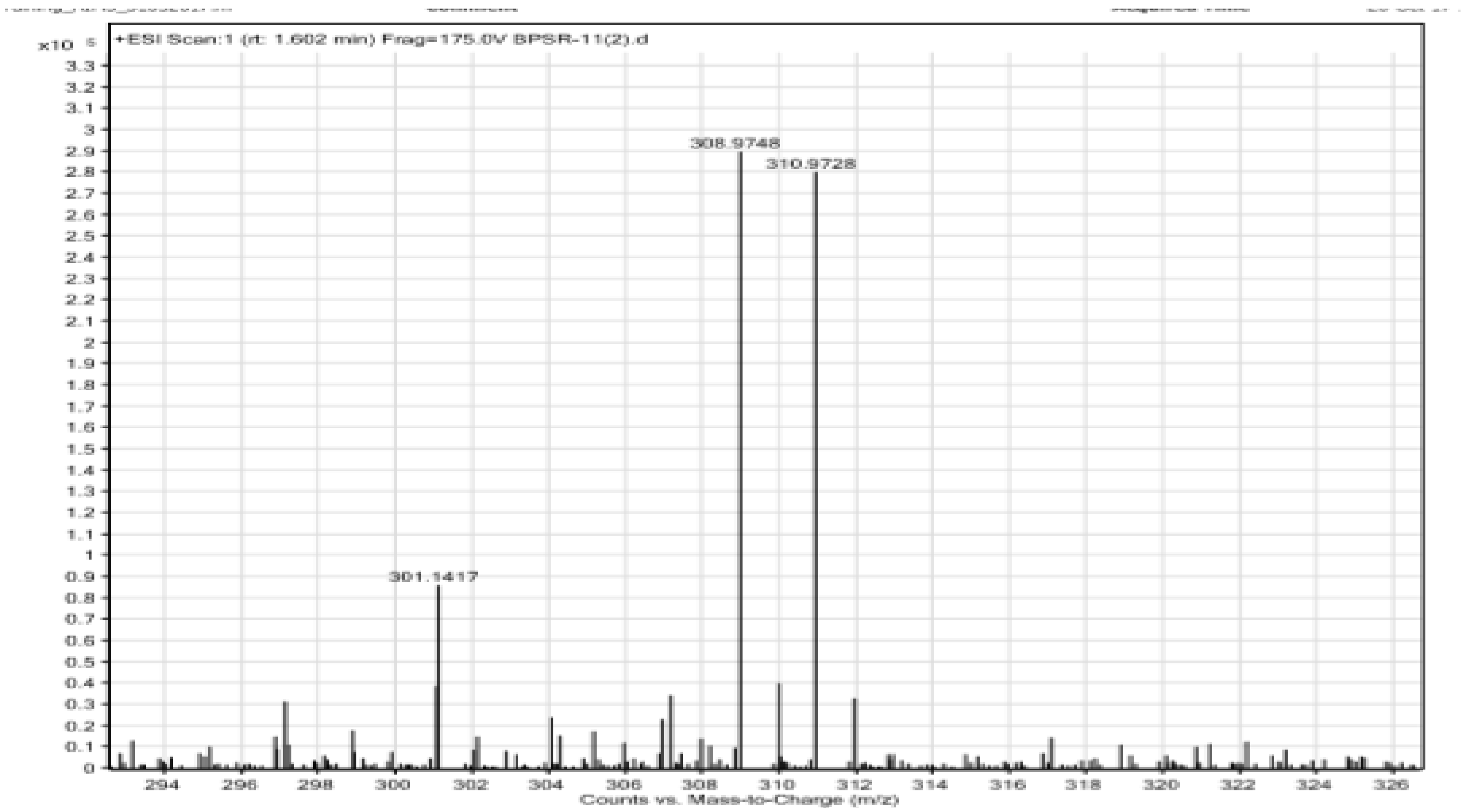

Figure S21. HRMS (ESI+) of compound **1**

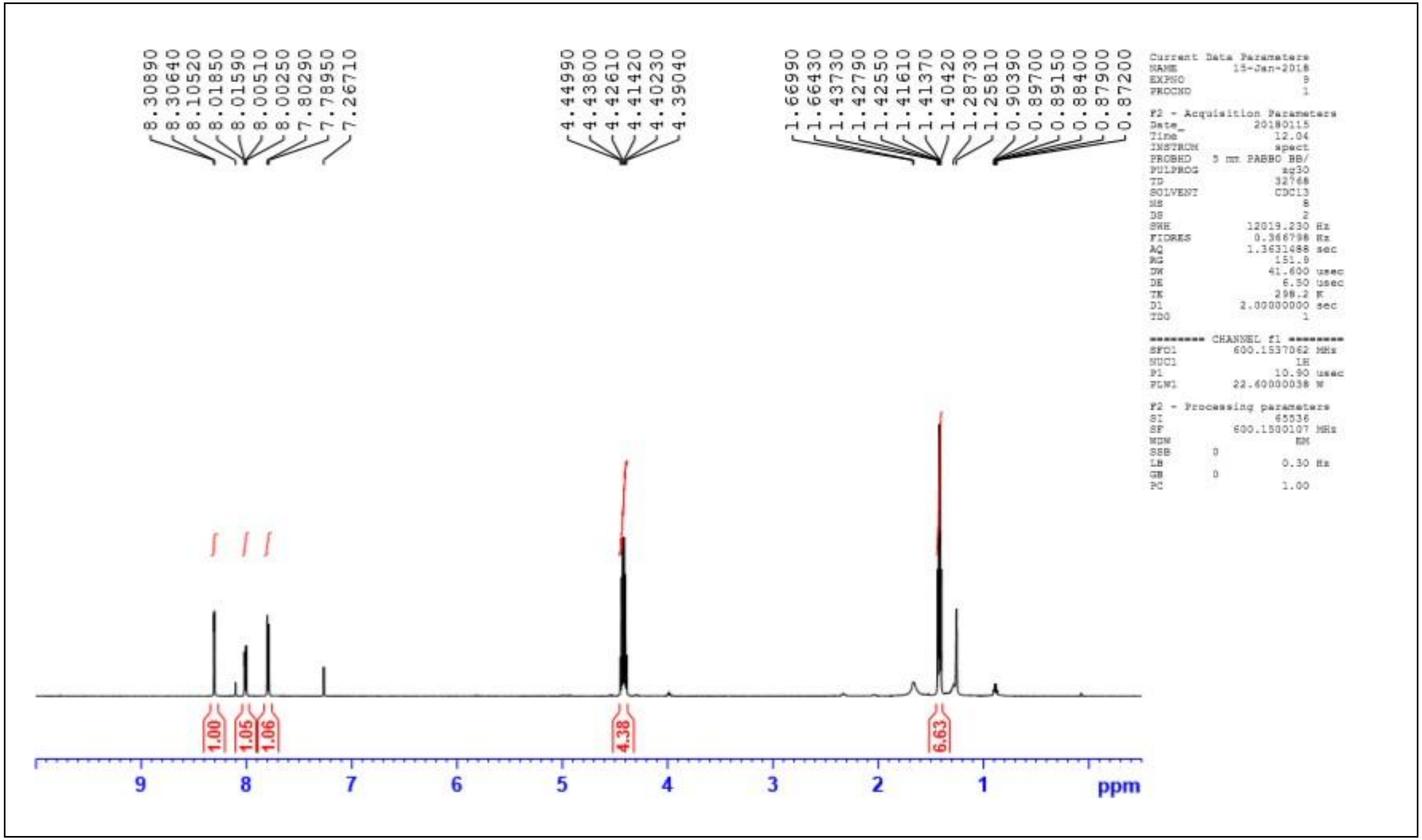

Figure S22. ¹H-NMR (600 MHz, $CDCl_3$) of compound **2**

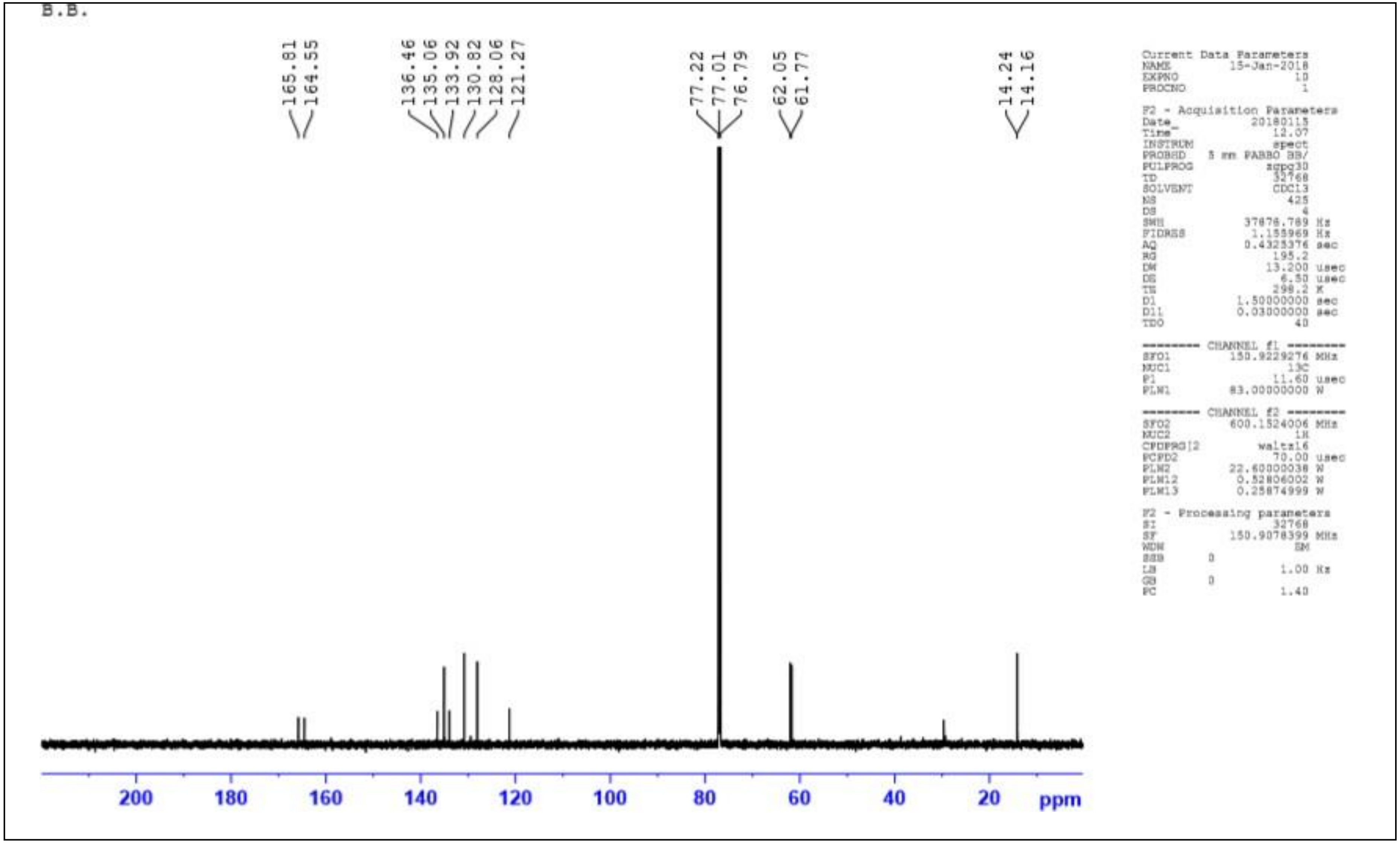

Figure S23. ^{13}C-NMR (150 MHz, $CDCl_3$) of compound **2**

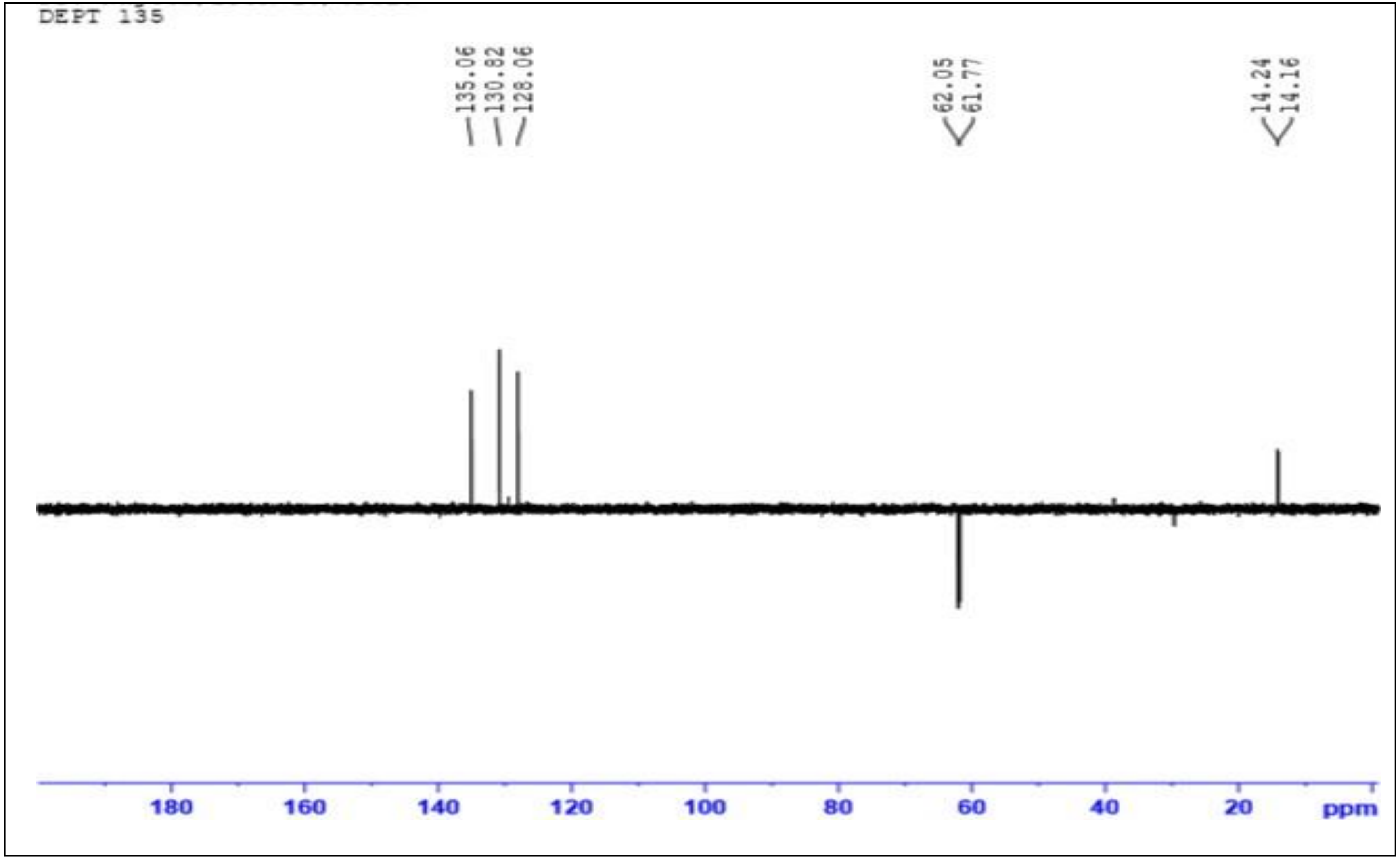

Figure S24. DEPT 135 of compound **2**

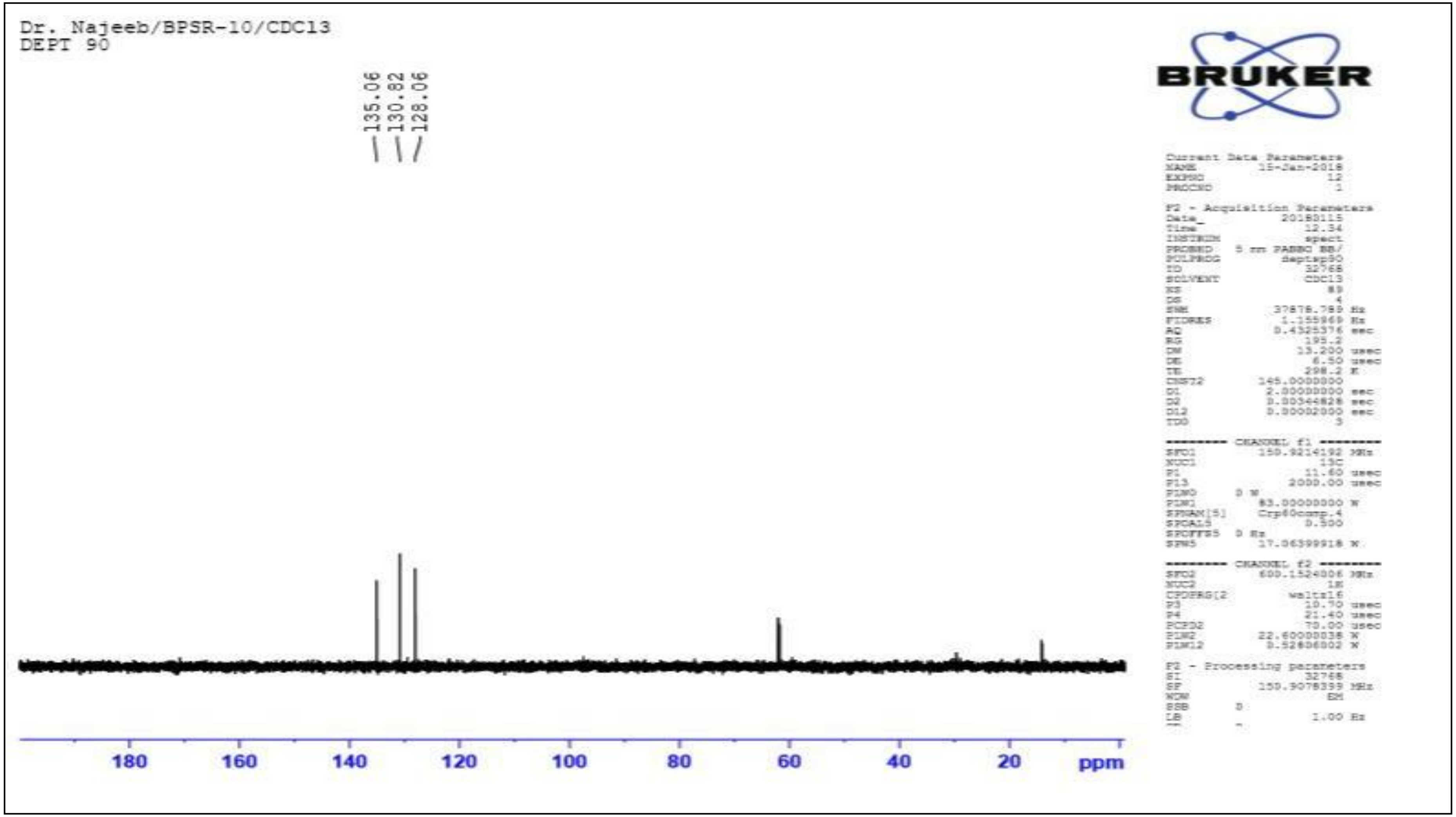

Figure S25. DEPT 90 of compound **2**

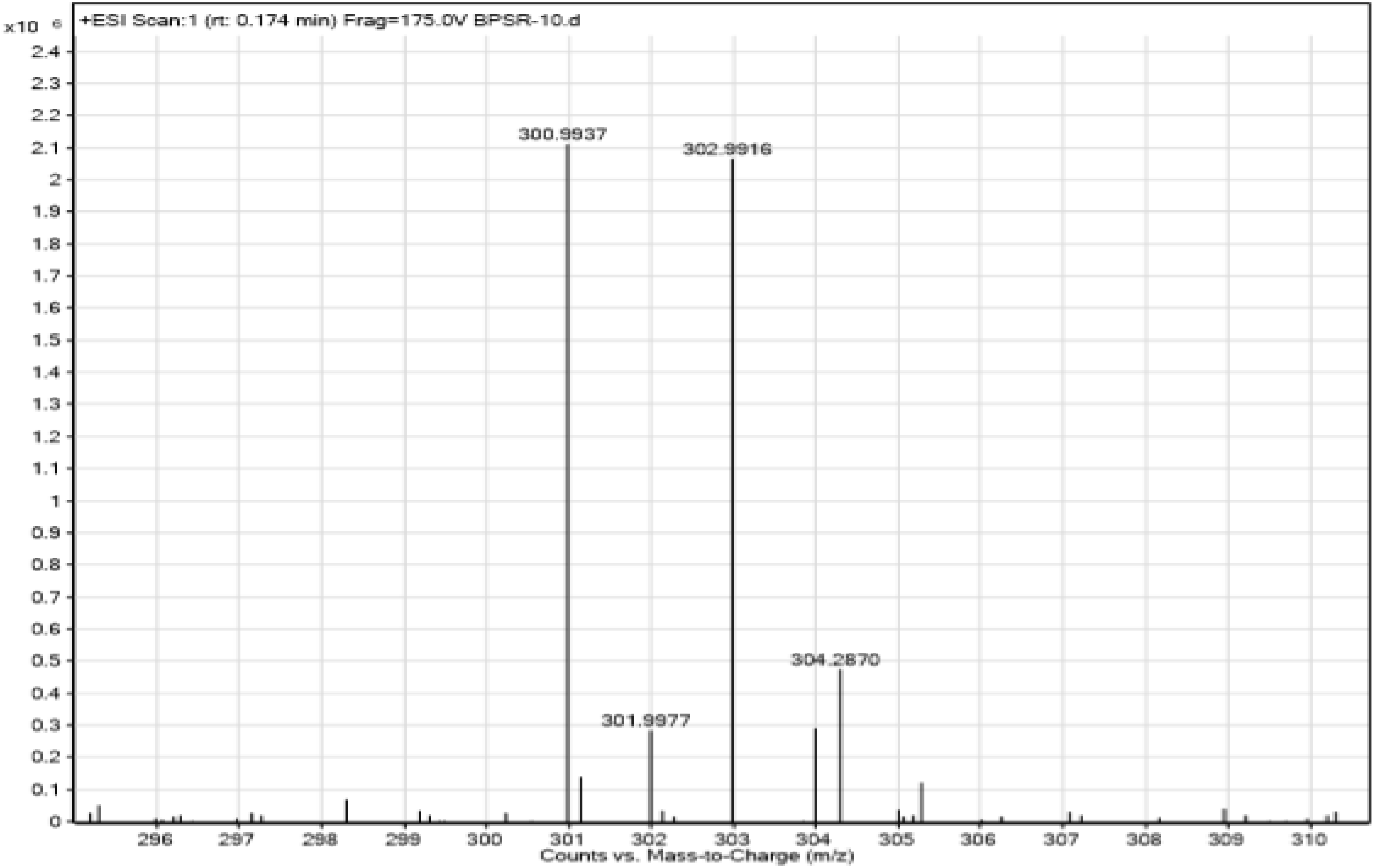

Figure S26. HRMS (ESI+) of compound **2**

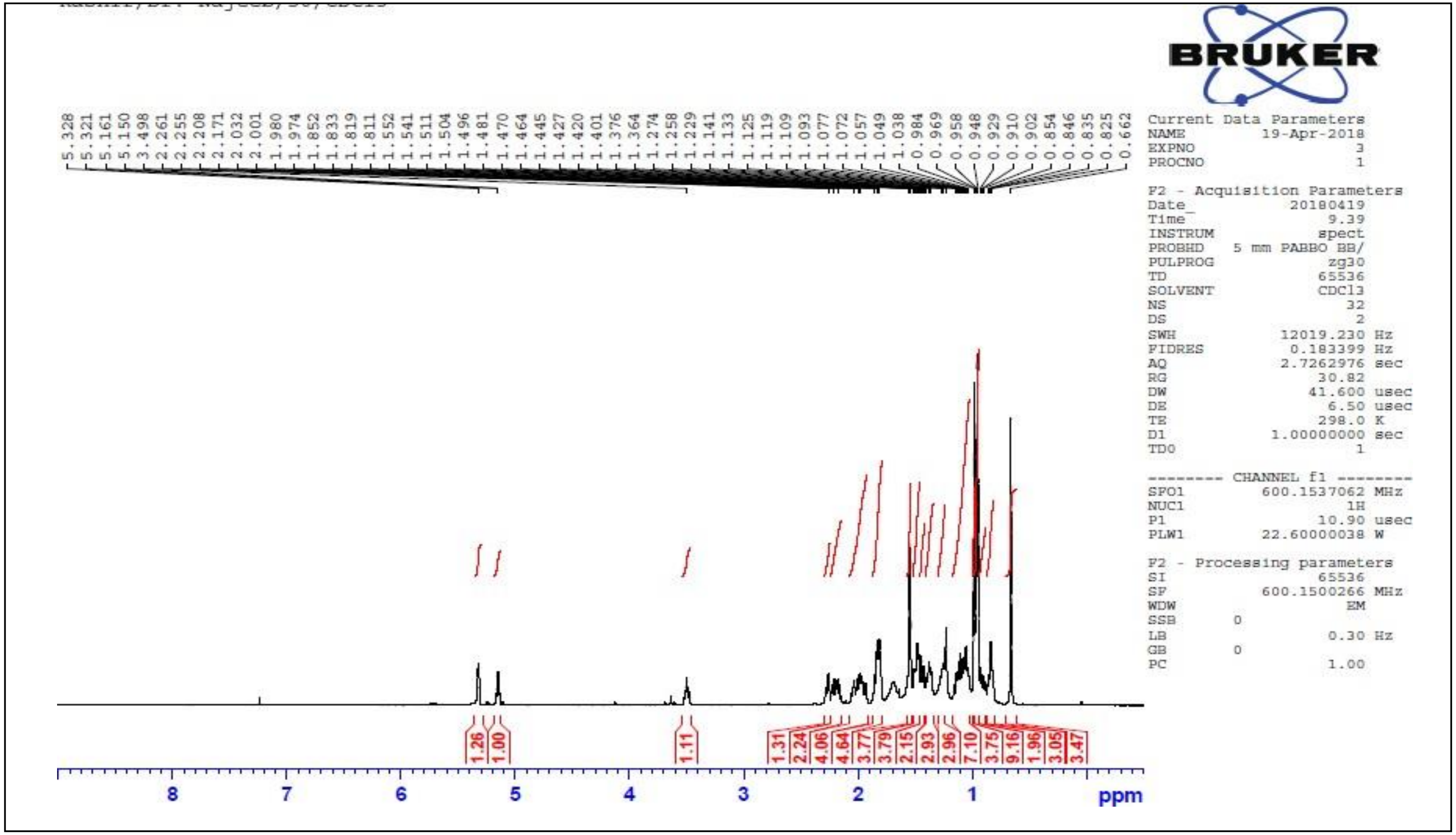

Figure S30. ^1H-NMR (600 MHz, $CDCl_3$) of compound **3**

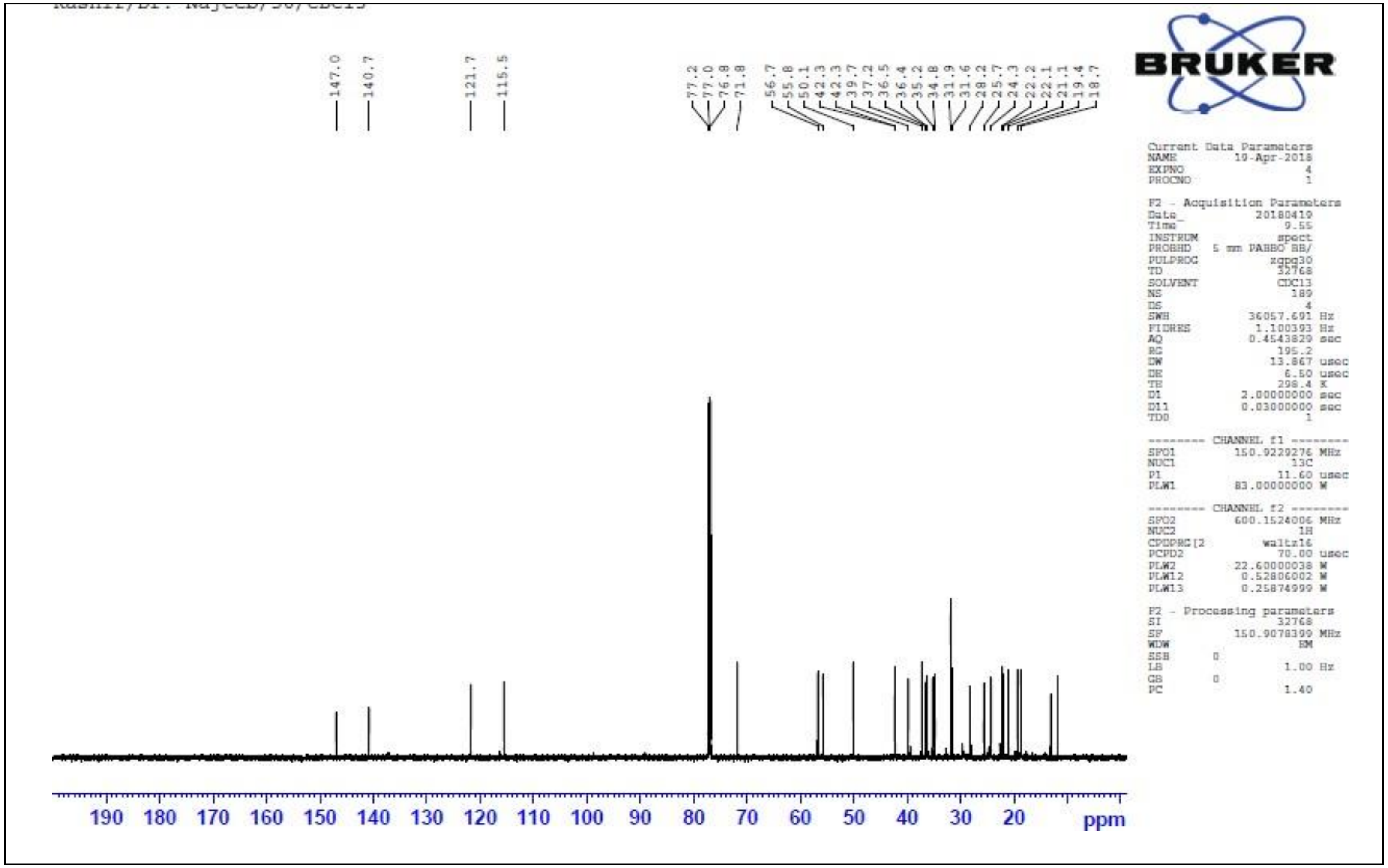

Figure S31. ^{13}C-NMR (150 MHz, $CDCl_3$) of compound **3**

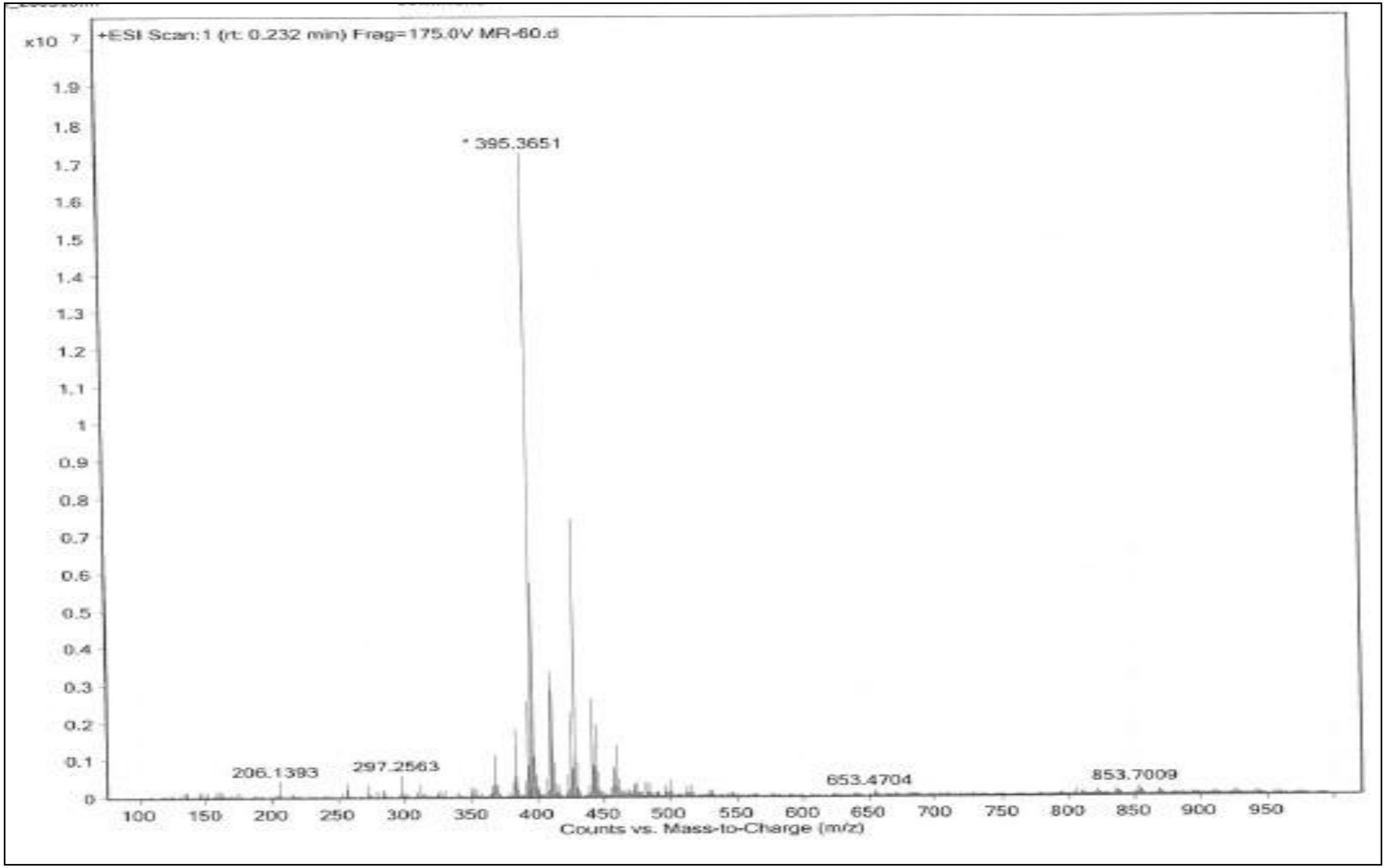

Figure S32. HRMS (ESI+) of compound **3**

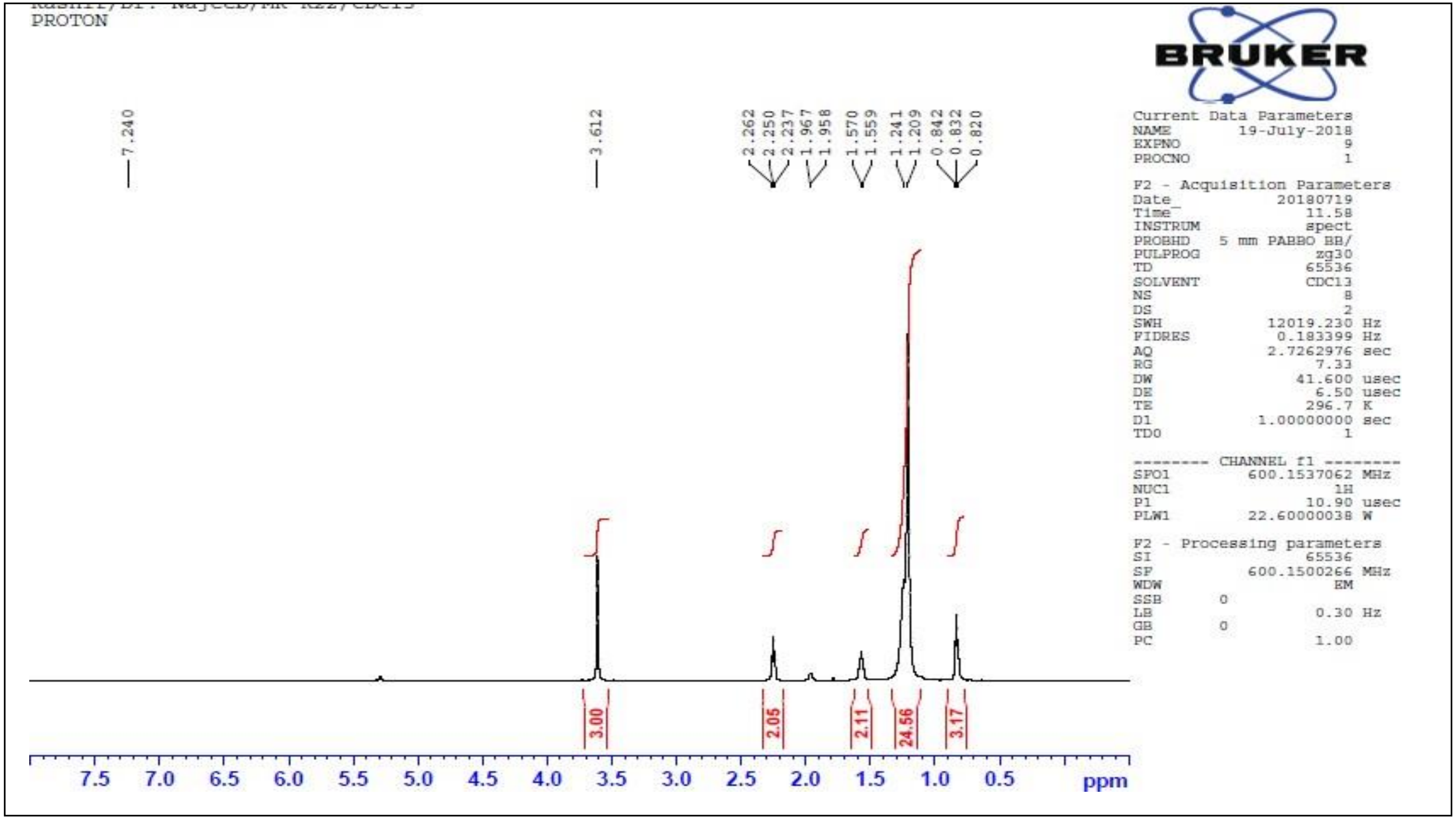

Figure S33. ^{1}H-NMR (600 MHz, $CDCl_3$) of compound **4**

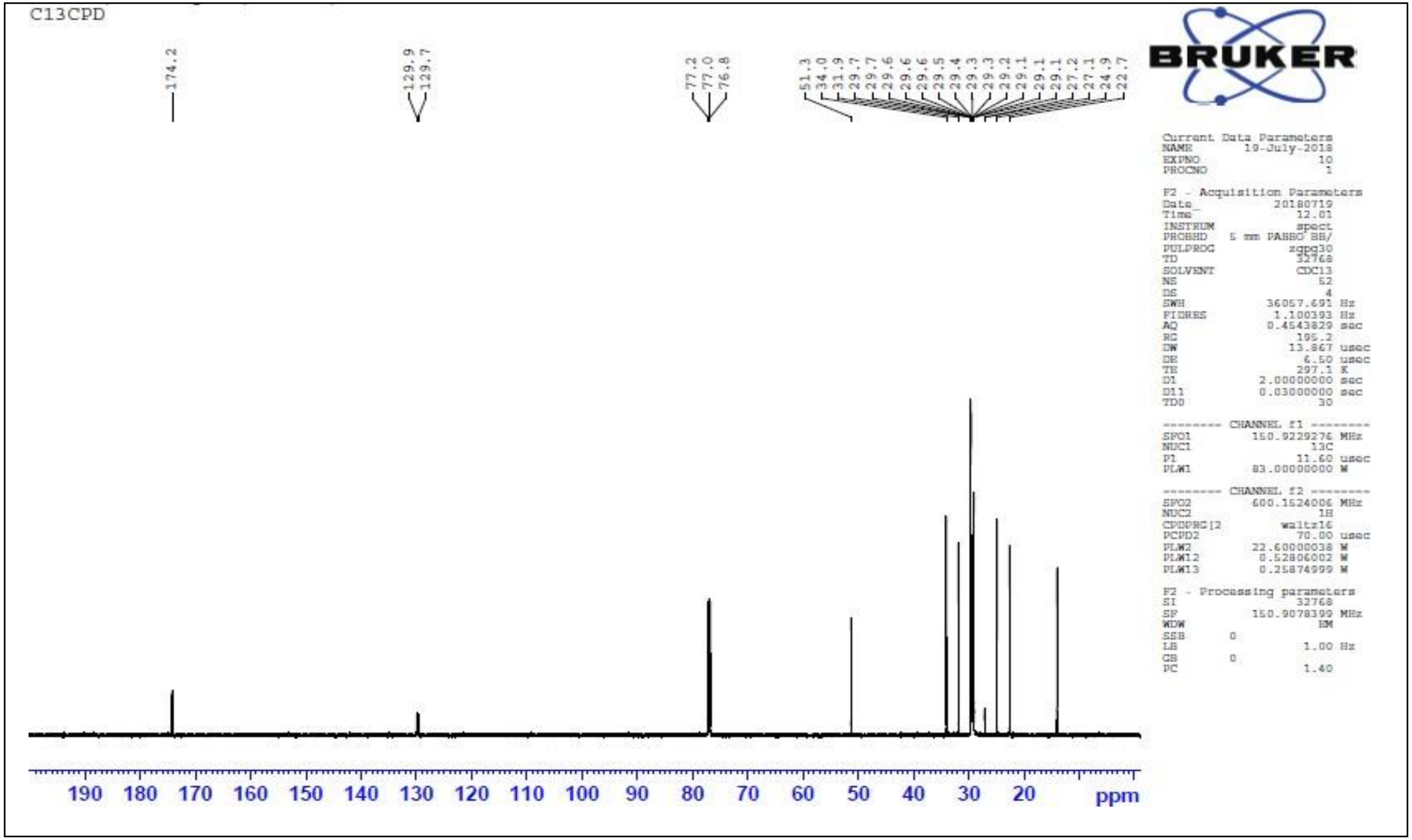

Figure S34. ^{13}C-NMR (150 MHz, $CDCl_3$) of compound **4**

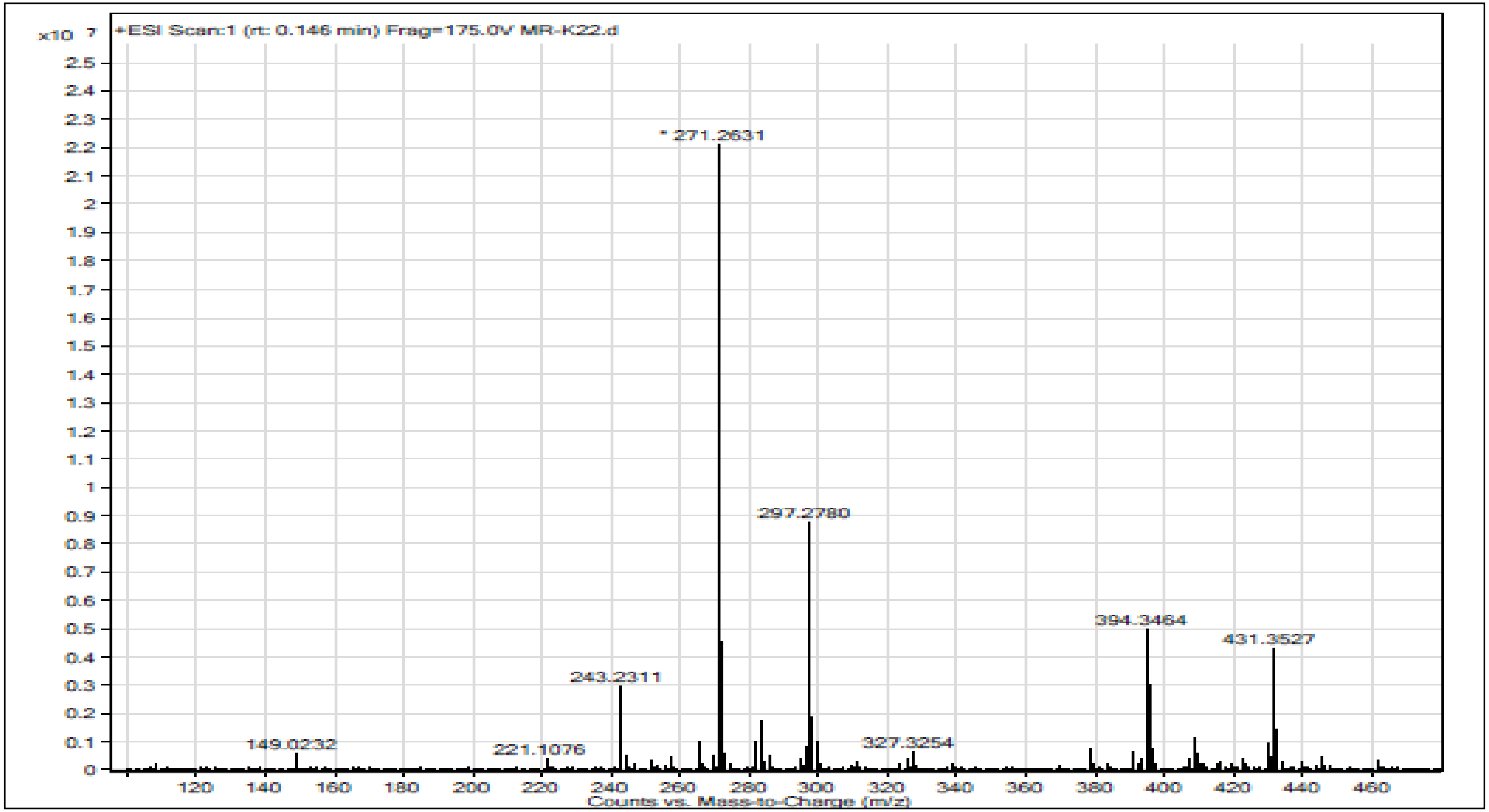

Figure S35. HRMS (ESI+) of compound **4**

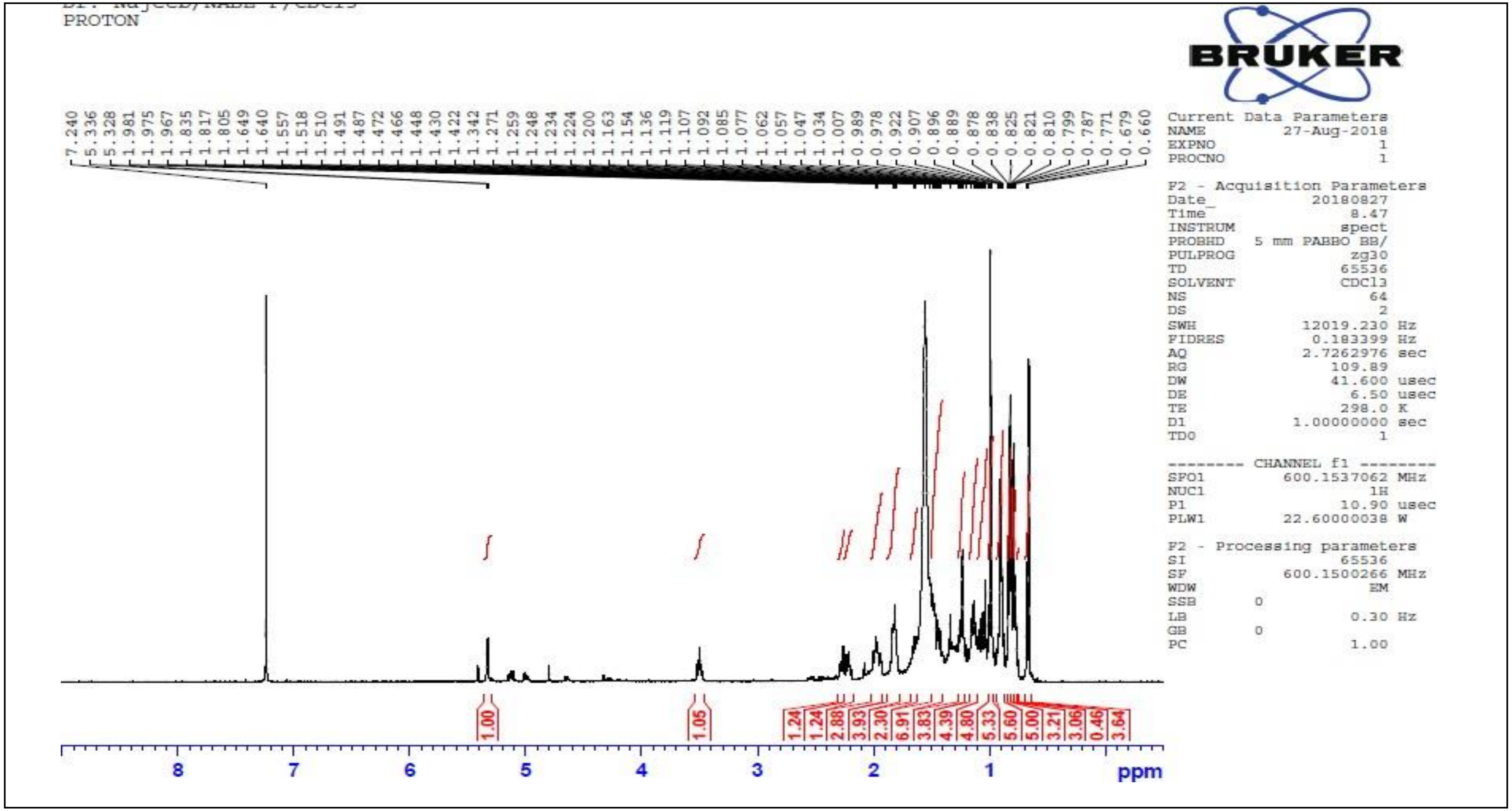

Figure S27. ^{1}H-NMR (600 MHz, $CDCl_3$) of compound **5**

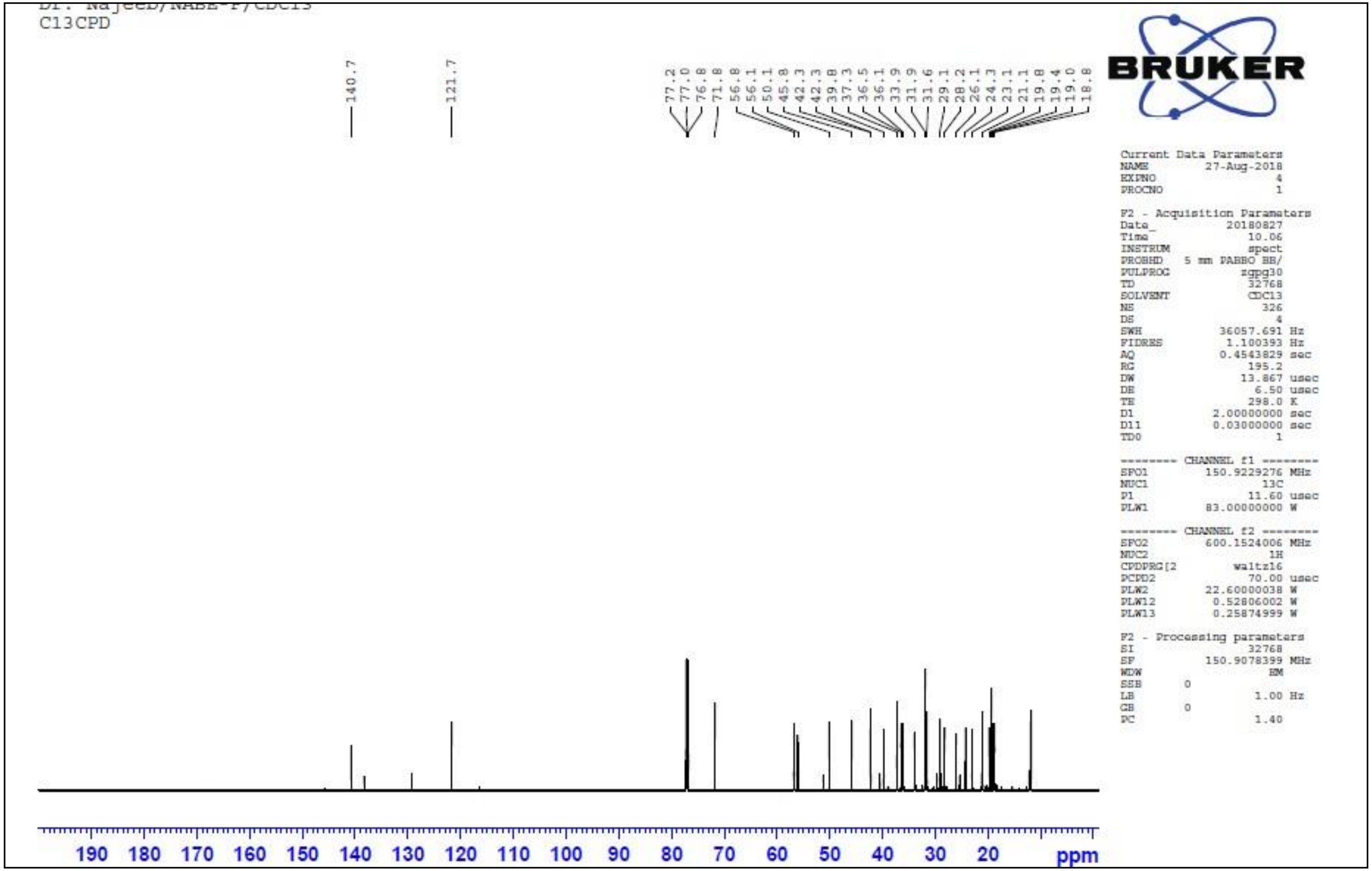

Figure S28. ^{13}C-NMR (150 MHz, $CDCl_3$) of compound **5**

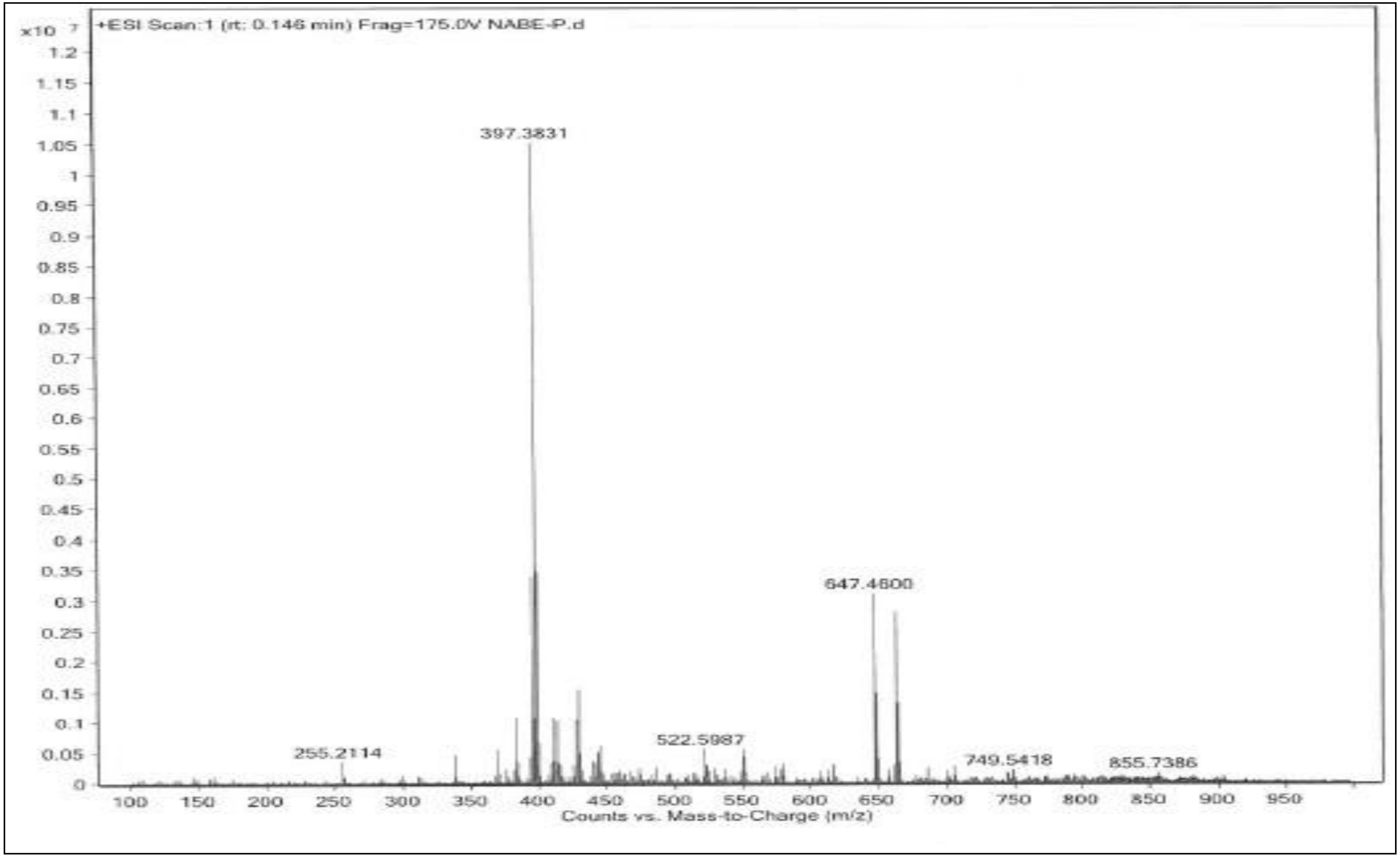

Figure S29. HRMS (ESI+) of compound **5**

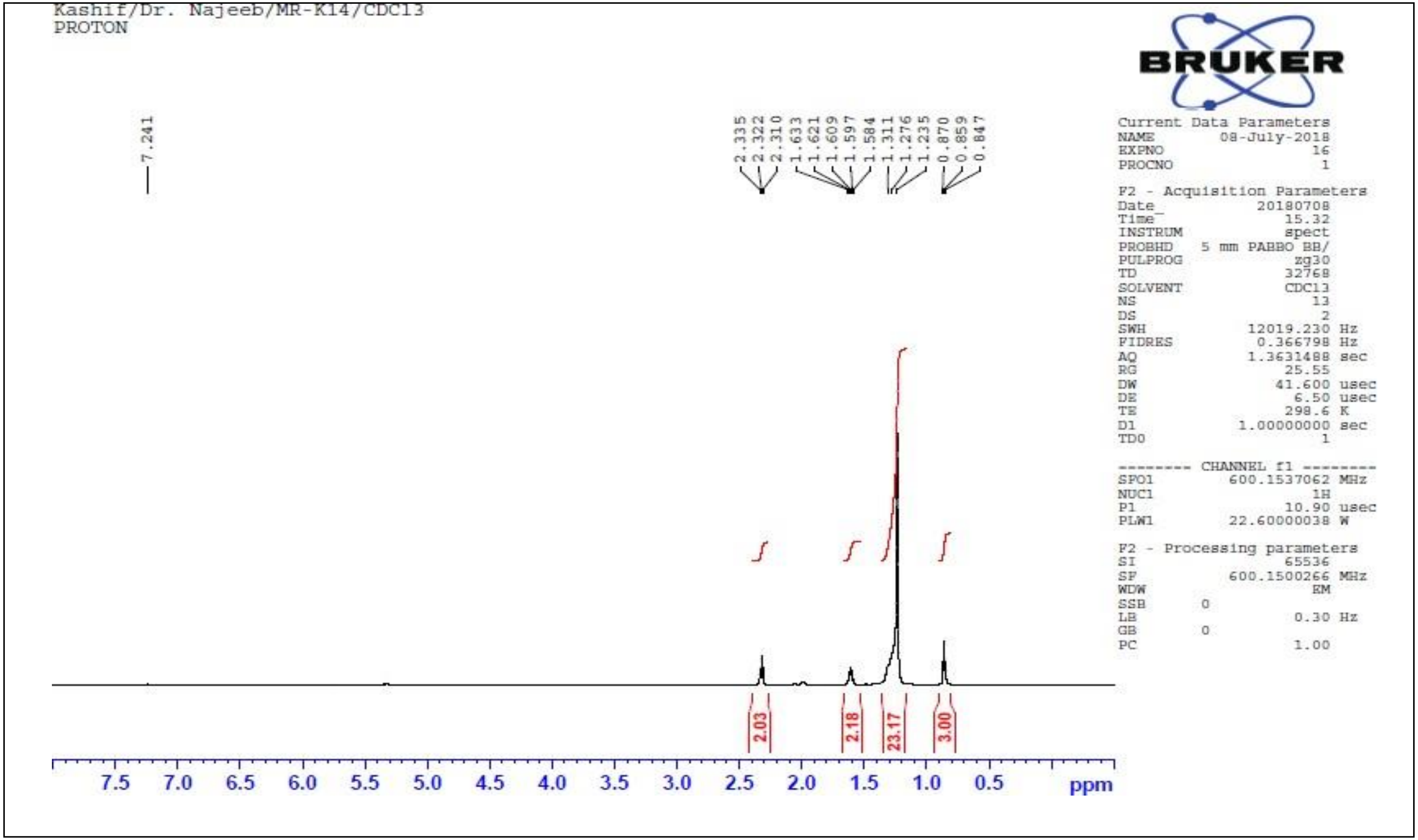

Figure S39. ^{1}H-NMR (600 MHz, $CDCl_3$) of compound **6**

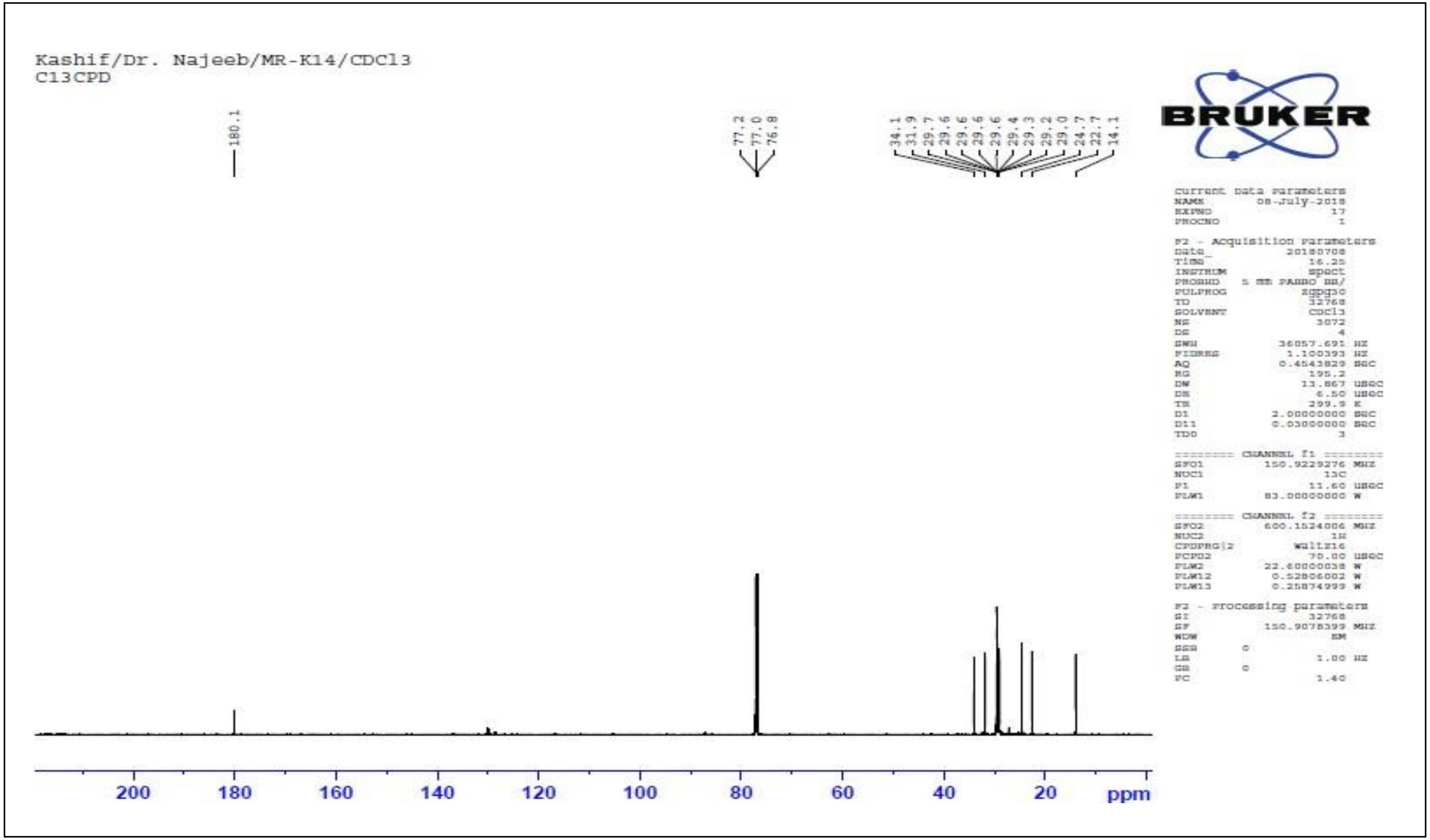

Figure S40. ^{13}C-NMR (150 MHz, $CDCl_3$) of compound **6**

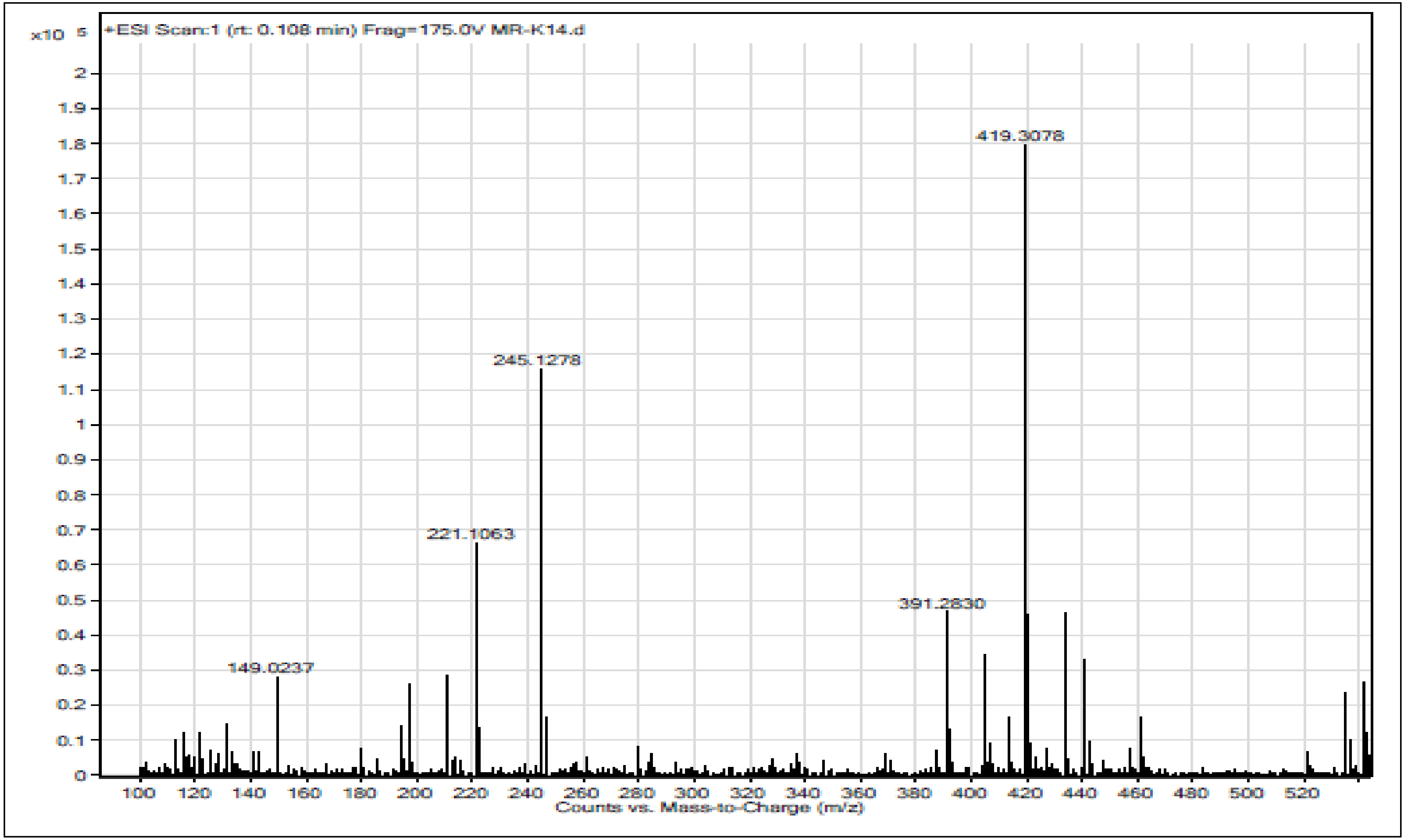

Figure S41. HRMS (ESI+) of compound **6**

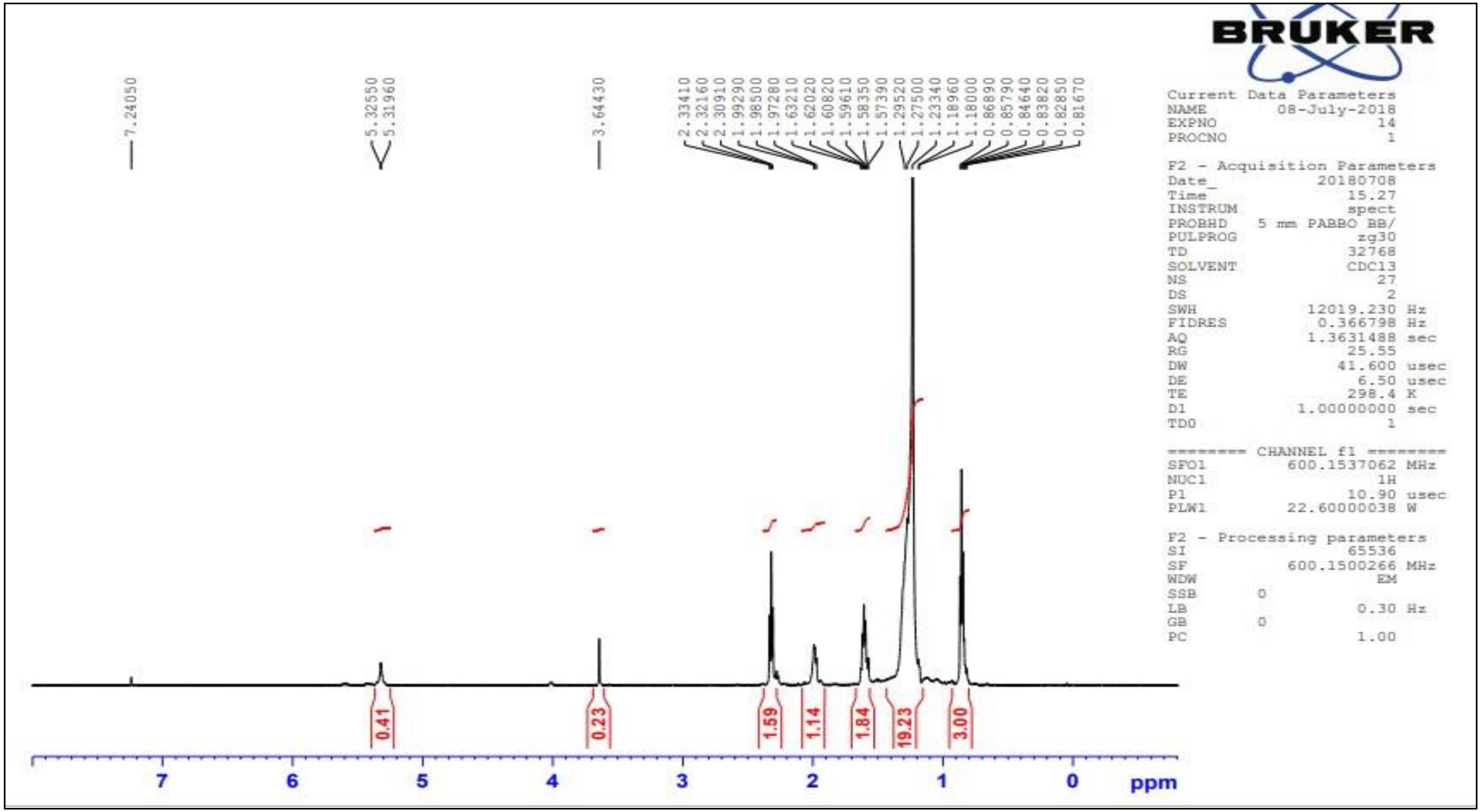

Figure S36. 1H-NMR (600 MHz, $CDCl_3$) of compound **7**

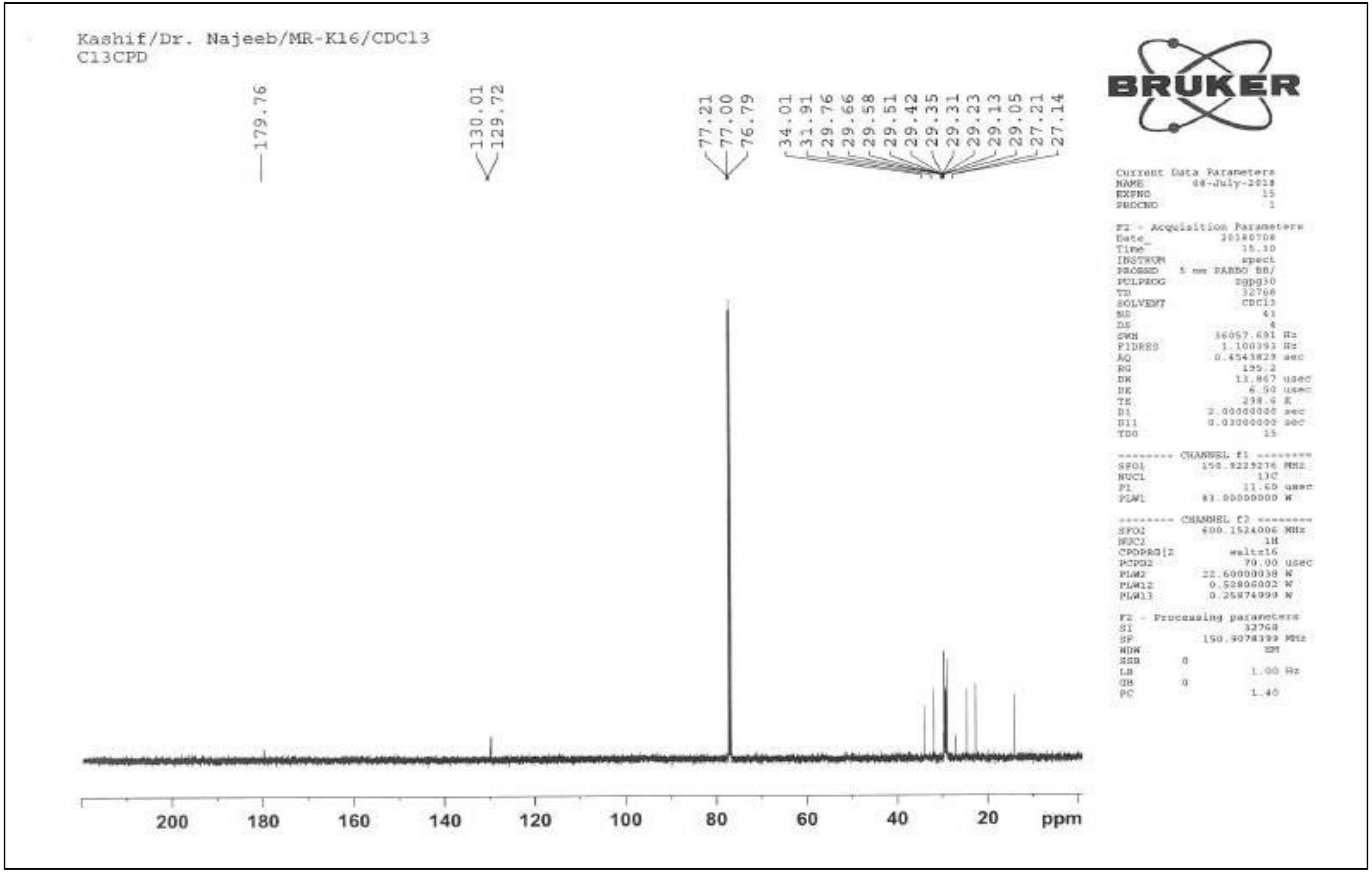

Figure S37. ^{13}C-NMR (150 MHz, $CDCl_3$) of compound **7**

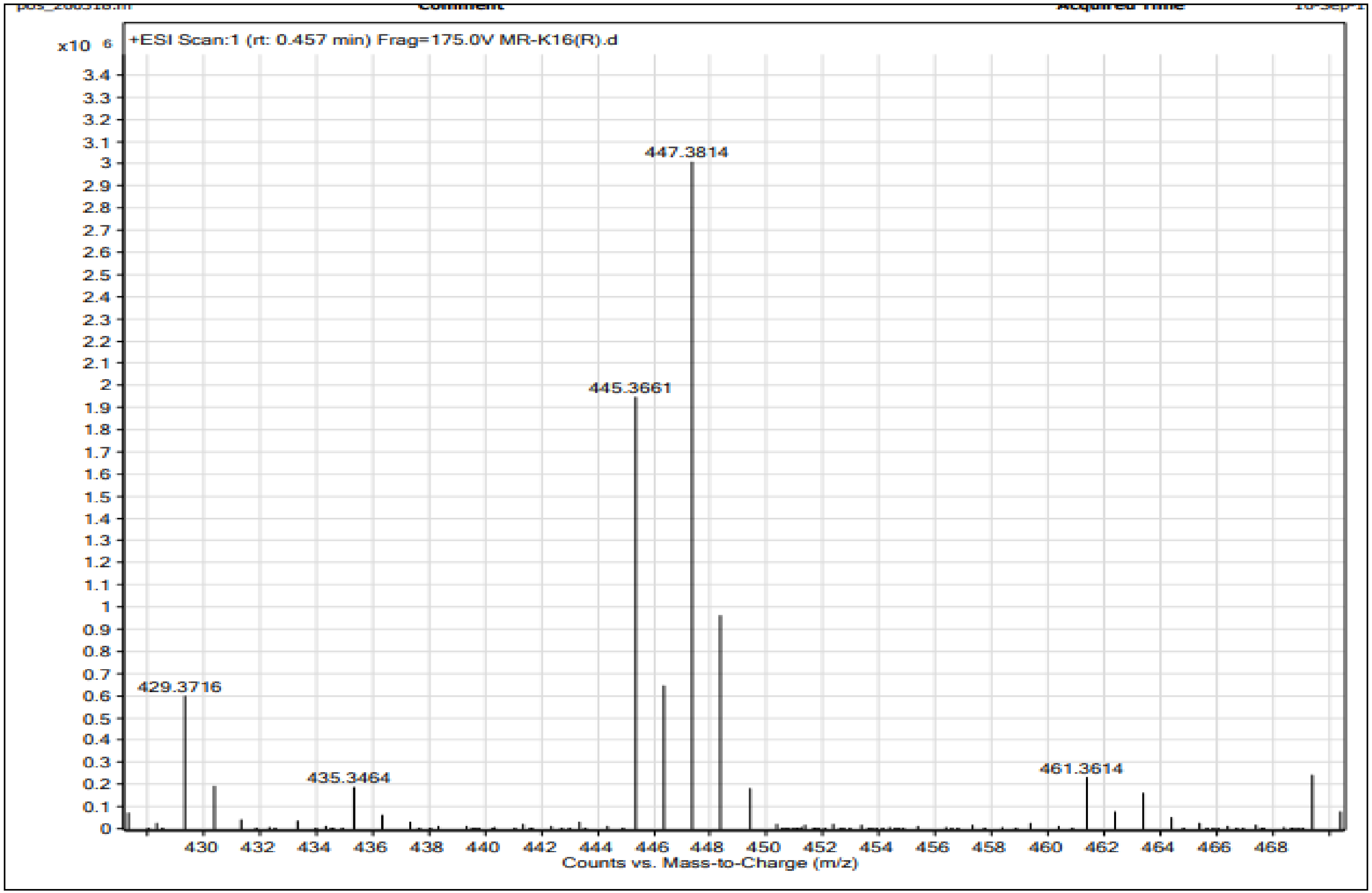

Figure S38. HRMS (ESI+) of compound **7**

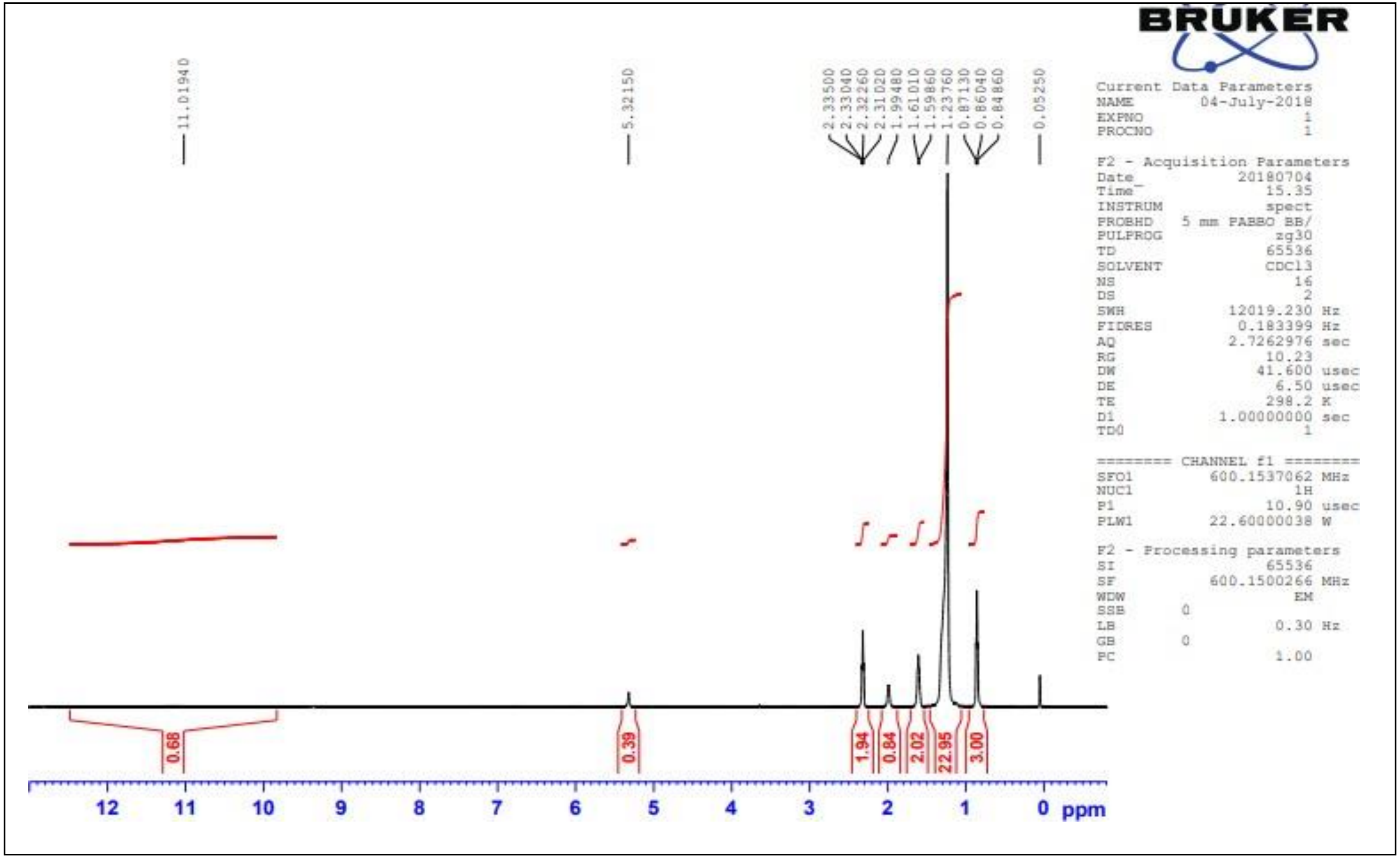

Figure S42. ^{1}H-NMR (600 MHz, $CDCl_3$) of compound **8**

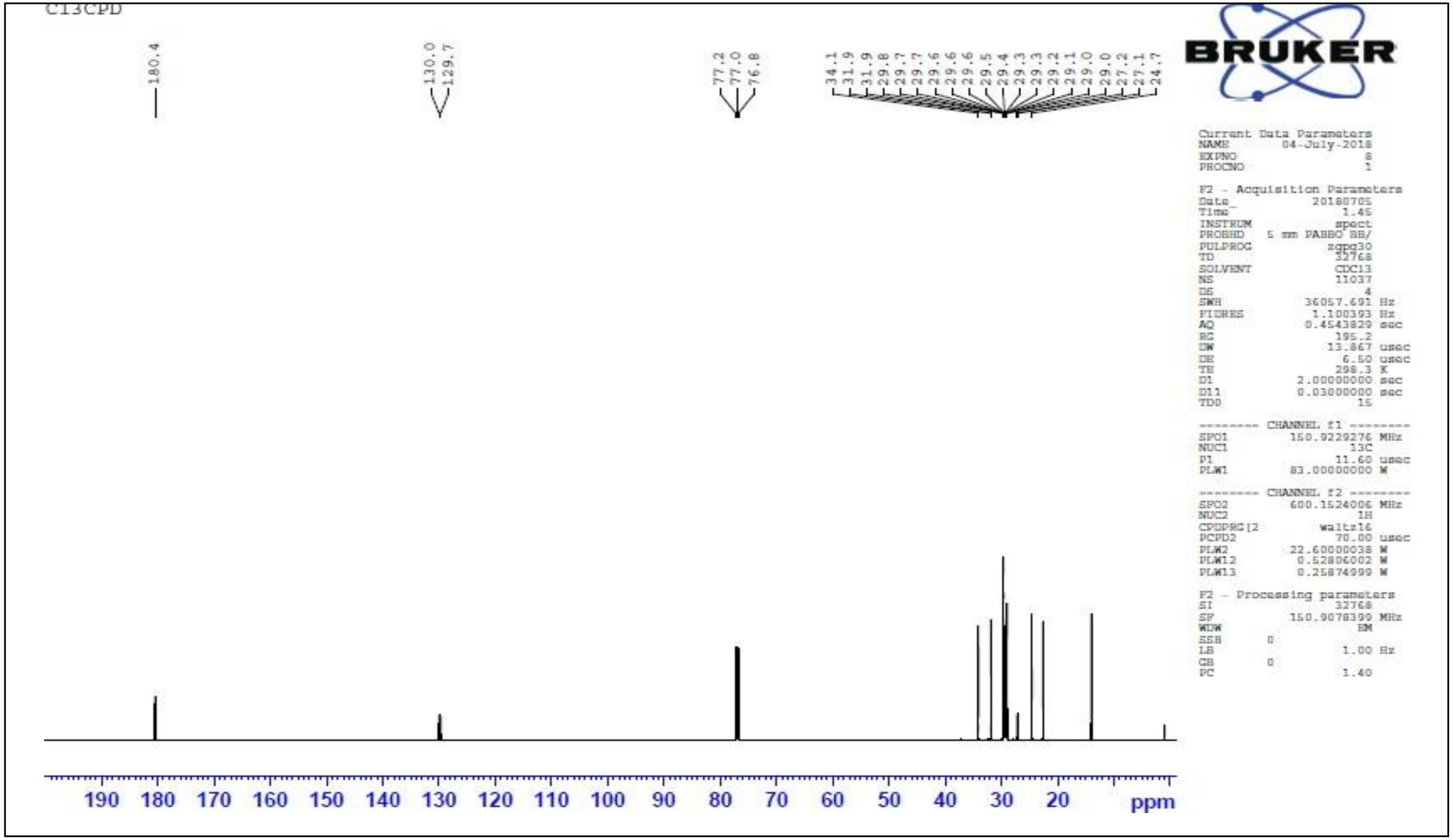

Figure S43. ^{13}C-NMR (150 MHz, $CDCl_3$) of compound **8**

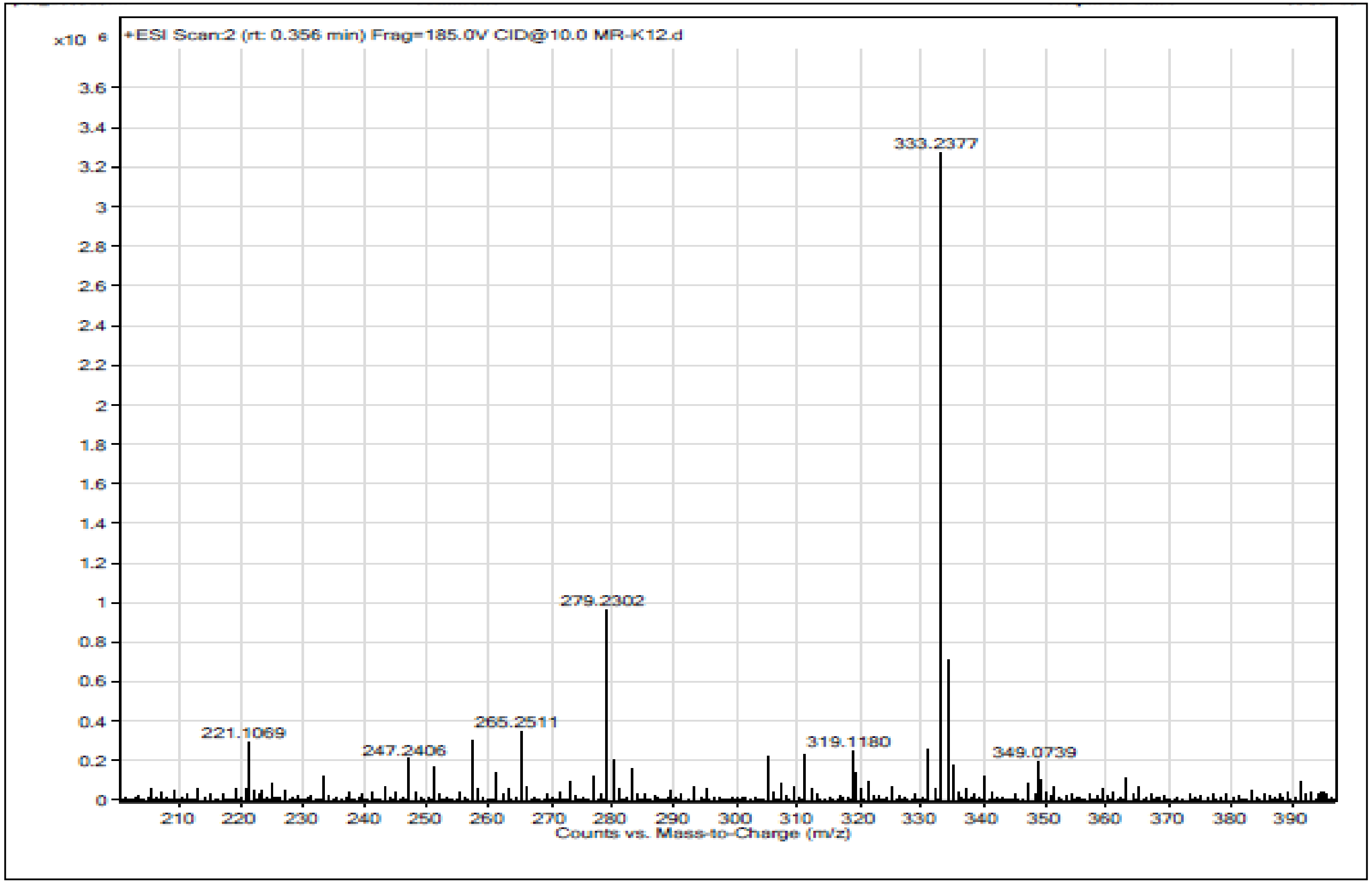

Figure S44. HRMS (ESI+) of compound **8**
